# Acute otitis externa: Consensus definition, diagnostic criteria and core outcome set development

**DOI:** 10.1371/journal.pone.0251395

**Published:** 2021-05-14

**Authors:** Matthew E. Smith, John C. Hardman, Nishchay Mehta, Gareth H. Jones, Rishi Mandavia, Caroline Anderson, Maha Khan, Aula Abdelaziz, Bakir Al-Dulaimy, Nikul Amin, Rajesh Anmolsingh, Bilal Anwar, Manohar Bance, Katherine Belfield, Mahmood Bhutta, Ruaridh Buchanan, Deepak Chandrasekharan, Michael Chu, Srikanth Chundu, Katherine Conroy, Gemma Crundwell, Mat Daniel, Jessica Daniels, Sujata De, Sian Dobbs, Jayesh Doshi, Matthew Farr, Tanjinah Ferdous, Eleni Fragkouli, Simon Freeman, Samit Ghosh, Emma Gosnell, S. Alam Hannan, Elliot Heward, Faisal Javed, Deepa John, Helen Nicholls, Anand V. Kasbekar, Haroon Khan, Hammad Khan, Sadie Khwaja, Bhik Kotecha, Madhankumar Krishnan, Nirmal Kumar, Tamara Lamb, Hannah Lancer, Joseph G. Manjaly, Marcos Martinez Del Pero, Fiona McClenaghan, Kristijonas Milinis, Nina Mistry, Hassan Mohammed, Elizabeth Morris, Stephen Morris-Jones, Jessica Padee, Surojit Pal, Sanjay Patel, Agamemnon Pericleous, Asad Qayyum, Maral Rouhani, Haroon Saeed, Mirusanthan Santhiyapillai, Kay Seymour, Sunil Sharma, Richard Siau, Arvind Singh, Emma Stapleton, Kate Stephenson, Gill Stynes, Bharathi Subramanian, Neil Summerfield, Chloe Swords, Aaron Trinidade, Antonia Tse, Emmanuel Twumasi, Harmony Ubhi, Samit Unadkat, Ananth Vijendren, Joe Wasson, Glen Watson, Glennis Williams, Janet Wilson, Alexander Yao, Ahmed Youssef, Simon K. W. Lloyd, James R. Tysome, on behalf of INTEGRATE (The UK ENT Trainee Research Network)

**Affiliations:** 1 Cambridge Ear Institute, Cambridge, United Kingdom; 2 The Royal Marsden Hospital London, London, United Kingdom; 3 Royal National ENT Hospital London, London, United Kingdom; 4 Aintree University Hospitals Liverpool, Liverpool, United Kingdom; 5 University College London Hospitals NHS Foundation Trust, London, United Kingdom; 6 Wexham Park Hospital Slough, Slough, United Kingdom; 7 Manchester University NHS Foundation Trust, Manchester, United Kingdom; 8 Stockport Medical Group, Stockport, United Kingdom; 9 Wythenshawe Hospital, Wythenshawe, United Kingdom; 10 Guy’s and St Thomas’ NHS Foundation Trust, London, United Kingdom; 11 Salford Royal Hospital, Salford, United Kingdom; 12 Cambridge University Hospitals NHS Foundation Trust, Cambridge, United Kingdom; 13 School of Medicine, University of Nottingham, Nottingham, United Kingdom; 14 Brighton Sussex University Hospitals, Brighton, United Kingdom; 15 Barts Health NHS Trust, London, United Kingdom; 16 Health Education North West, Manchester, United Kingdom; 17 Anglia Ruskin University, Cambridge, United Kingdom; 18 Nottingham University Hospitals NHS Trust, Nottingham, United Kingdom; 19 Tameside and Glossop NHS Integrated Care Trust, Ashton-under-Lyne, United Kingdom; 20 Alder Hey Children’s Hospital NHS Foundation Trust, Liverpool, United Kingdom; 21 Heartlands Hospital Birmingham, Birmingham, United Kingdom; 22 University of Sheffield, Sheffield, United Kingdom; 23 Oxford University Hospitals Foundation Trust, Oxford, United Kingdom; 24 Pennine Acute Trust, Manchester, United Kingdom; 25 Royal Bolton Hospital, Farnworth, United Kingdom; 26 Devon General Practice, Exeter, Devon, United Kingdom; 27 Royal Preston Hospital, Fulwood, United Kingdom; 28 Nuffield Health Brentwood Hospital, Brentwood, United Kingdom; 29 Wrightington, Wigan and Leigh NHS Foundation Trust, Wigan, United Kingdom; 30 Queen Elizabeth Hospital Birmingham, Birmingham, United Kingdom; 31 West Suffolk Hospital, Bury St Edmunds, United Kingdom; 32 Worcestershire Acute Hospitals NHS Trust, Worcester, United Kingdom; 33 Newcastle Upon Tyne University Hospitals NHS Foundation Trust, Newcastle Upon Tyne, United Kingdom; 34 Nuffield Department of Primary Care Health Sciences, United Kingdom; 35 University of Manchester, Manchester, United Kingdom; 36 London North West University Healthcare NHS Trust, London, United Kingdom; 37 West Middlesex University Hospital, Isleworth, United Kingdom; 38 North West Anglia NHS Foundation Trust, Peterborough, United Kingdom; 39 Imperial College Healthcare NHS Trust, London, United Kingdom; 40 Liverpool University Hospitals NHS Foundation Trust, Liverpool, United Kingdom; 41 Birmingham Children’s Hospital, Birmingham, United Kingdom; 42 Ipswich Hospital, Ipswich, United Kingdom; 43 Southend University Hospital NHS Foundation Trust, Southend-on-Sea, United Kingdom; 44 Lister Hospital Stevenage, Stevenage, United Kingdom; 45 East Kent Hospitals NHS Foundation Trust, Canterbury, United Kingdom; 46 Sheffield Teaching Hospitals NHS Foundation Trust, Sheffield, United Kingdom; 47 Grove Park Terrace Surgery, London, United Kingdom; 48 Dudley NHS Foundation Trust, Dudley, United Kingdom; University of Maryland School of Medicine, UNITED STATES

## Abstract

**Objective:**

Evidence for the management of acute otitis externa (AOE) is limited, with unclear diagnostic criteria and variably reported outcome measures that may not reflect key stakeholder priorities. We aimed to develop 1) a definition, 2) diagnostic criteria and 3) a core outcome set (COS) for AOE.

**Study design:**

COS development according to Core Outcome Measures in Effectiveness Trials (COMET) methodology and parallel consensus selection of diagnostic criteria/definition.

**Setting:**

Stakeholders from the United Kingdom.

**Subjects and methods:**

Comprehensive literature review identified candidate items for the COS, definition and diagnostic criteria. Nine individuals with past AOE generated further patient-centred candidate items. Candidate items were rated for importance by patient and professional (ENT doctors, general practitioners, microbiologists, nurses, audiologists) stakeholders in a three-round online Delphi exercise. Consensus items were grouped to form the COS, diagnostic criteria, and definition.

**Results:**

Candidate COS items from patients (n = 28) and literature (n = 25) were deduplicated and amalgamated to a final candidate list (n = 46). Patients emphasised quality-of-life and the impact on daily activities/work. Via the Delphi process, stakeholders agreed on 31 candidate items. The final COS covered six outcomes: pain; disease severity; impact on quality-of-life and daily activities; patient satisfaction; treatment-related outcome; and microbiology. 14 candidate diagnostic criteria were identified, 8 reaching inclusion consensus. The final definition for AOE was ‘diffuse inflammation of the ear canal skin of less than 6 weeks duration’.

**Conclusion:**

The development and adoption of a consensus definition, diagnostic criteria and a COS will help to standardise future research in AOE, facilitating meta-analysis. Consulting former patients throughout development highlighted deficiencies in the outcomes adopted previously, in particular concerning the impact of AOE on daily life.

## Introduction

Acute otitis externa (AOE) is a common inflammatory condition of the ear canal which remains poorly defined, and has no widely accepted diagnostic criteria. In addition, there is no standardisation of the outcomes assessed in AOE interventional trials.

Core outcome sets (COS) are agreed standardised sets of outcomes that represent the minimum that should be measured and reported in all clinical studies of a specific condition [1]. The validity of a COS depends on its development, which must include working with key stakeholders to prioritise what may be a large number of candidate outcomes. The Core Outcome Measures in Effectiveness Trials (COMET) initiative has published guidance on COS development [1], which has been recognised internationally as best practice. The development of a validated COS improves consistency in outcome reporting [2], which facilitates evidence synthesis.

The lack of clear diagnostic criteria or a COS for AOE have been responsible for heterogeneity in published studies, and a subsequent weak evidence base for clinical practice. Evidence-based management for AOE is essential to improve treatment outcomes and the patient experience, and reduce inappropriate use of antibiotics that may contribute to microbial resistance [3].

To facilitate future research into AOE, we aimed to:

1. Follow COMET guidelines to develop a COS to be used for adults with AOE undergoing treatment.
2. Determine diagnostic criteria for AOE.
3. Establish a definition for AOE, including the timepoint at which to consider otitis externa chronic

## Methods

Development of the COS was based on methodology from the COMET handbook [1], the diagnostic criteria and definition were developed in parallel. A three-stage strategy was implemented: firstly, consulting with former patients for their perspectives; secondly, systematically evaluating the relevant literature; and thirdly, reaching consensus amongst key stakeholders. The protocol was registered on the COMET database (ID 1321).

### Ethical considerations

The Health Research Authority Decision Tool confirmed that NHS Research Ethics Committee approval was not required. At each stage informed consent was obtained from contributors.

### Stage 1: Outcomes derived from former patients

Former patients were surveyed, using semi-structured interviews, to understand their views and experiences regarding AOE and how to measure treatment success. They were asked five questions: ‘How did the ear infection affect you?’; ‘What were the difficult aspects of your infection?’; ‘What were the difficult aspects of your treatment?’; ‘How could the impact of the condition be reduced?’; and ‘How could treatment be improved?’. Former patients from two NHS Trusts in North West England contributed to this advisory group, representing a broad demographic in terms of age and gender. Candidate items for the COS were extracted from interviews by two investigators working independently.

### Stage 2: Comprehensive literature review

To capture all previously adopted definitions, diagnostic criteria and outcomes for AOE, a literature search was performed in August 2018 (MEDLINE 1946–2018, EMBASE 1974–2018) for all studies reporting the effectiveness of any intervention for AOE in adults. Subject strategies were combined with the Cochrane Collaboration search strategy [4], (example of our search strategy in S1 Fig). PsycInfo was searched (Ovid 1806–2018) for patient reported outcome measures for AOE. Additionally, a grey literature search was undertaken on EMCARE. Results were de-duplicated and abstracts uploaded to the Rayyan systematic review app (www.rayyan.qcri.org) [5]. Each abstract was screened by two reviewers working independently. Records were excluded if they did not pertain to treatments for adults with AOE, if they were case series/reports, or published in non-English language.

Two researchers then independently reviewed the full texts. Inclusion criteria were: reporting of inclusion criteria and/or outcome measures for clinical trials; and the reporting of diagnostic criteria for AOE.

Diagnostic criteria and outcomes were then extracted from included articles into separate documents by two researchers working independently. Given an anticipated high volume of search results, and to avoid extracting the same data from multiple articles, a staged approach was adopted for the extraction of diagnostic criteria and outcomes, based on publication date. The first extraction included studies published 2011–2019 and a second covered 2008–2010. The diagnostic criteria and outcomes from the second extraction were compared to those in the first. If either new diagnostic criteria or outcomes were found in the second extraction, subsequent extractions would be performed in 2-year intervals, stopping once no further new data were found.

#### Preparation of candidate items for the core outcome set and diagnostic criteria

The outcomes extracted from stages 1 and 2 were grouped according to a taxonomy [1] and, where appropriate, de-duplicated or combined by steering committee consensus. For the outcomes, language and terminology used by the patients was incorporated wherever possible. Similarly, related diagnostic criteria were combined where appropriate. All identified candidate items for the COS and diagnostic criteria were included in the Delphi process as part of stage 3.

### Stage 3: Stakeholder consensus process

A three-round online modified Delphi process was used to agree consensus amongst key stakeholders for the COS, diagnostic criteria and definition of duration for AOE [6]. Stakeholder groups were classified as either professionals or former patients. The professionals stakeholder group comprised the following sub-groups: Consultant otologists; Consultant non-otologist ENT surgeons; ENT registrars; junior doctors and specialist nurses in ENT; general practitioners with or without a specialist interest in ENT; microbiologists; and audiologists. The former patients group represented adult patients who had received treatment for AOE within the preceding four months; they were not involved in the consensus process for the diagnostic criteria or definition.

The Google Forms online survey platform was used to anonymously collect ratings for the candidate items for the COS and diagnostic criteria (Google LLC, CA, USA; available at docs.google.com/forms). Prior to each round, the surveys were piloted for readability, as well as face and content validity, by independent ENT registrars and consultants.

#### Outcome and diagnostic criteria consensus process

The stakeholder rating process for the identification of core outcomes and diagnostic criteria was conducted in parallel. While patient stakeholders only considered outcomes, the professional group had the online forms divided into two sections, one for core outcomes and the other for diagnostic criteria (example form S1 File). Participants from the professionals and former patients groups were asked to rate their agreement with each item on an interval scale of 1–9, where 1 indicated lowest importance and 9 indicated highest importance. An ‘unable to score’ option was also provided. Items scoring 1–3 were deemed of limited importance, items scoring 4–6 deemed as important but not critical, and those scoring 6–9 considered of critical importance. Consensus to include was pre-defined as ≥70% participants scoring as 7–9 AND <15% scoring as 1–3. Consensus to exclude was pre-defined as ≥70% participants scoring as 1–3 AND <15% scoring as 7–9.

**Fig 1:**
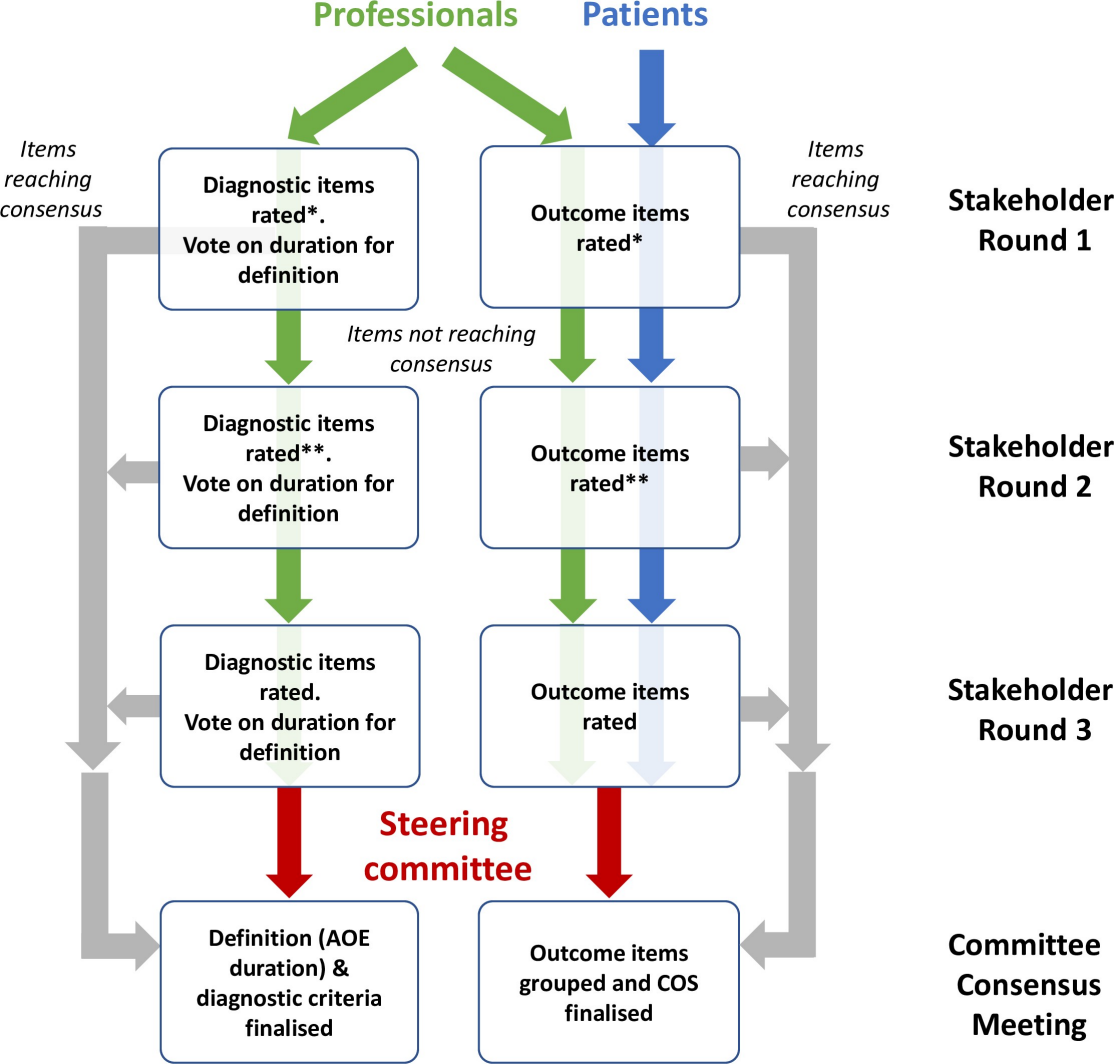
Flow diagram showing the three-stage Delphi consensus process.

The Delphi process (outlined in a flow diagram, Fig 1) consisted of three rounds of anonymised questioning, with consensus criteria being applied after each round and feedback provided for items not reaching consensus. In round 1, participants were invited to suggest both additional outcome and diagnostic criteria items they considered important which had not been included, and to suggest re-wording to improve clarity and reduce ambiguity. In round 2, items reaching consensus within each stakeholder group were not re-presented to that group. Participants were reminded that they could change their responses in subsequent round for items that had not yet reached consensus. To facilitate this, the pooled responses for items yet to reach consensus were displayed as charts immediately above the relevant items. New items suggested by participants were included in rounds 2. In round 3, to highlight any differences in responses between the two groups, the pooled responses from each group were presented separately to all participants.

Following the Delphi exercise, tables of items reaching and not-reaching consensus were compiled. Outcomes and diagnostic criteria were considered separately at a meeting of the steering committee.

Outcomes not meeting consensus criteria were scrutinised by the steering committee, who took a final decision on inclusion. This decision was based on several factors, including how near items were to reaching consensus, and gave particular weight to responses from the former patients group. Outcomes reaching consensus were then grouped by the steering committee to form the COS.

To establish the set of diagnostic criteria, candidate items reaching consensus through the Delphi process were summarised by the steering committee in a consensus process to form a clinically-relevant statement. Similarly a definition for AOE was formed by consensus.

#### Definition consensus process

The process to determine a definition, including the timepoint at which AOE should be considered chronic, required professional stakeholders to respond to an additional question, presented at each of the three Delphi rounds. This question took a different format to the item-rating questions, whereby an interval range of timepoints was provided (between 2–12 weeks). The professional stakeholders selected the timepoint they felt represented the limit of ‘acute’ otitis externa, with results of the previous round presented in round 2 and 3. Consensus was pre-defined as ≥70% of respondents selecting a single interval in any of the three rounds.

## Results

### Stage 1: Outcomes derived from former patients

**Fig 2:**
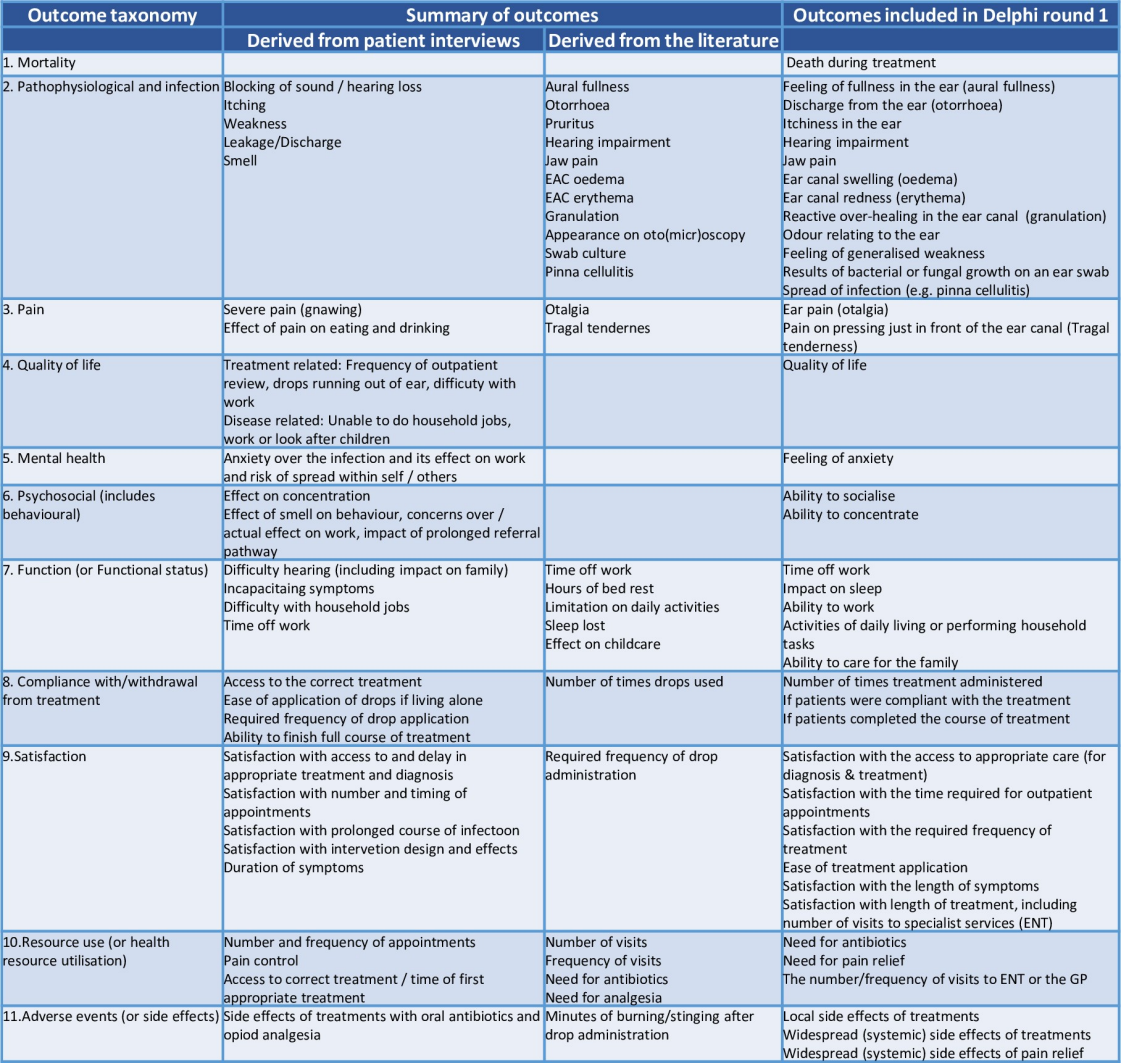
Outcomes taken from patient interviews and the literature combined and de-duplicated to form the candidate outcomes for the first round Delphi exercise.

To generate candidate items for the COS, nine former patients were consulted for their opinions. Their ages ranged from 17 to 79 years and four were male. For eight individuals, pain and discharge were prominent features of AOE e.g. “*There was a constant*, *severe*, *gnawing pain in the ear*”. The effect of AOE on work, family life and social interaction was also frequently described e.g. *“I didn’t even leave the house for the first week because of it*. *It smelt awful”*. AOE was also described as having an effect on anxiety and mental health *“I kept worrying I would be fired”*. Satisfaction with the treatments they had received also featured prominently, specifically: their experiences of the timing of treatments; the local and systemic effects of treatments (including side effects); the frequency of administration of treatments; and the ease of use of treatments, e.g. *“the frequency of ear drop application is frustrating”*. Twenty-eight candidate items were extracted for potential inclusion in the COS and mapped to the taxonomy (Fig 2).

### Stage 2: Comprehensive literature review

**Fig 3:**
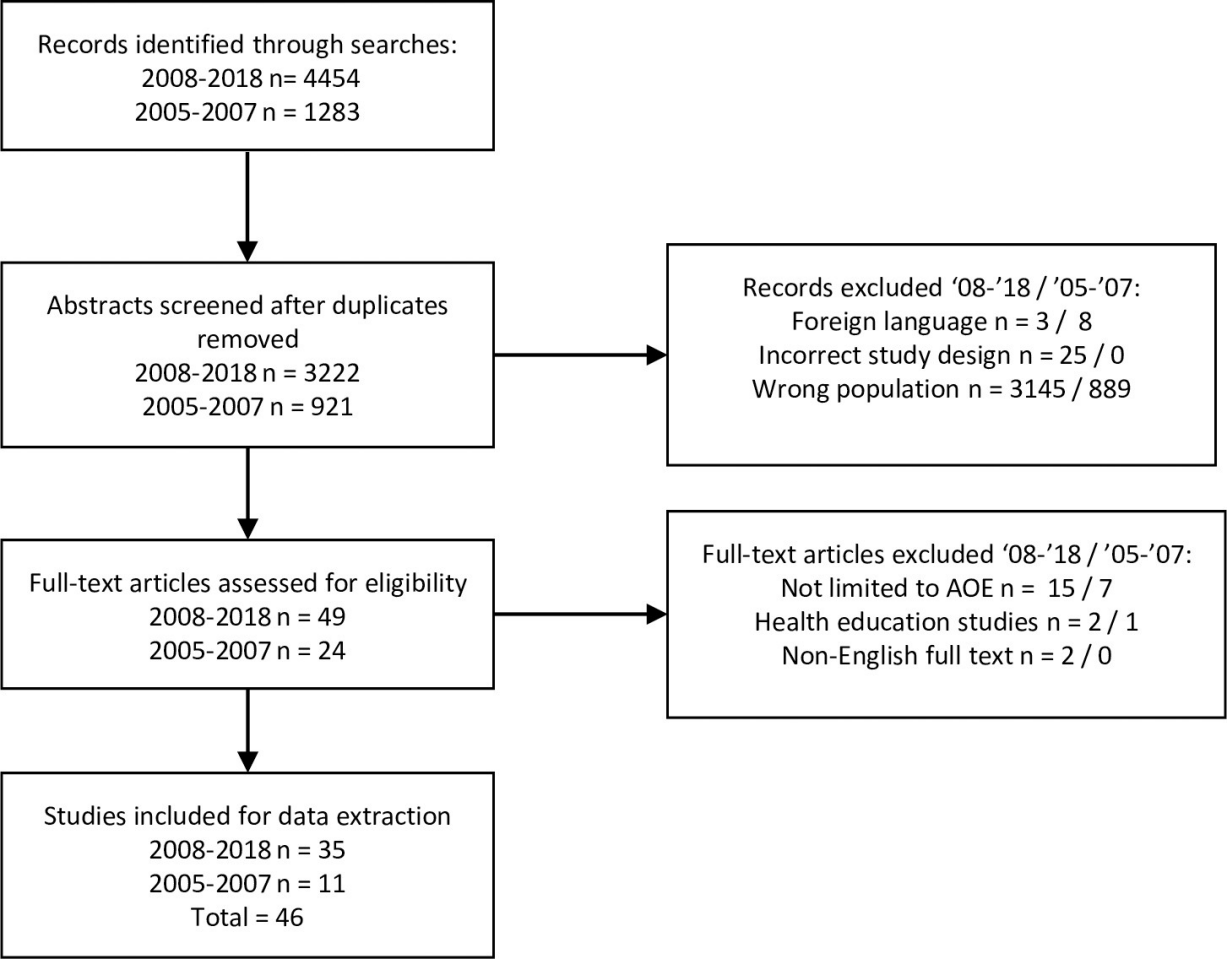
PRISMA diagram showing search results for both extraction periods.

The search strategy identified 3,222 unique articles within the period 2008–2018. Abstract then full text review led to the exclusion of 3,152 articles (Fig 3). 24 articles were identified published in the first extraction period and 11 in the second. Novel outcomes and diagnostic criteria were identified in the second extraction period, and so a third was conducted to include search results from 2005–2007. An additional 921 unique results led to 11 articles for inclusion in the third extraction (Fig 3), though no new outcomes or diagnostic criteria were identified in this period. In total therefore, 46 articles were included for final analysis. Over the three extractions, 25 outcomes and 16 diagnostic criteria were identified for inclusion in the list of candidate items and were mapped to the taxonomy (Fig 2 and S1 Table).

### Stage 3: Stakeholder consensus process

#### Preparation of candidate items for the core outcome set and diagnostic criteria

Lists of candidate items from stages 1 and 2 were combined and deduplicated (Fig 2). The steering committee reviewed the wording of these outcomes to ensure they were suitable for lay stakeholders.

#### Delphi results

Three rounds of the Delphi online questionnaire were completed. For those participants engaging in the round 1 of the Delphi, the overall retention rate to completion of round 3 was 82.5% (n = 99/120, range 57.1% for GPs and 100% for nurses and ENT registrars) (Table 1).

**Table 1:** Participating stakeholders in Delphi rounds 1 to 3.

Stakeholder groups	Invited (n)	Completed Round 1 (n)	Completed Round 2 (n)	Completed Round 3 (n)	Retention rate: round 1 to 3 (%)
Former patients	21	10	7	6	**60.0**
Consultant otologist	25	22	22	20	**90.9**
Consultant non-otologist	19	15	13	11	**73.3**
ENT Registrar	31	27	27	27	**100.0**
Junior doctors / Nurses	19 / 6	13 / 4	11 / 4	10 / 4	**76.9 / 100**
General practitioner	19	14	8	8	**57.1**
Microbiologist	7	5	4	4	**80.0**
Audiologist	15	10	10	9	**90.0**
**Total**	**157**	**120**	**106**	**99**	**82.5**

#### AOE core outcome set

The professionals and former patients stakeholder groups appraised 43 candidate items for inclusion in the COS for AOE. The former patients reached consensus for inclusion on 24 items in the first round, a further eight items in the second round, a further three in the final round. No items reached consensus for exclusion in any round. In total, 35 items met consensus for inclusion in the former patients stakeholder group. The professional stakeholders reached consensus for inclusion on 17 items in the first round, 10 in the second and four in the final round. No items met criteria for exclusion in the first round, but one item was excluded in the second round and a further item in the third. Therefore, 31 items met consensus for inclusion and two for exclusion in the professionals stakeholder group (S2 Table).

It was agreed by the steering committee that given the number of included outcomes, none of those reaching consensus in the patient or professional groups alone would be included in the final set.

**Fig 4:**
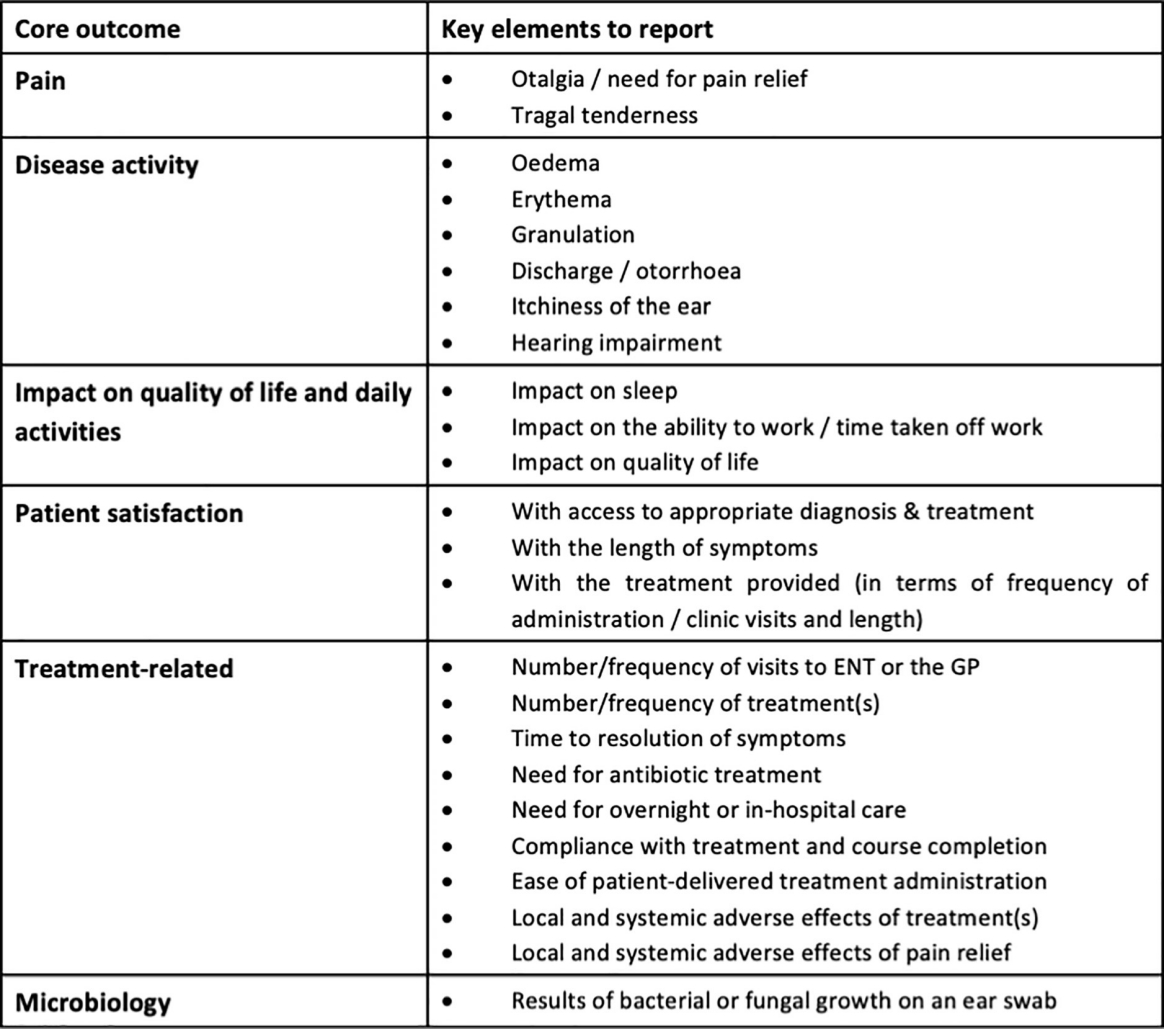
Core outcome set for acute otitis externa.

Owing to the large number of candidate items meeting inclusion in both stakeholder groups, the steering committee agreed by majority vote to group similar items to create the final COS. Additionally, to limit the number of candidate items considered for inclusion in the final COS, items reaching consensus in only either the professionals or the former patients stakeholder group were discounted. To further limit the candidate items for consideration for following items were combined: ‘time off work’ and ‘ability to work’; ‘satisfaction with the frequency of treatments’ and ‘required number of visits’; and ‘pain’ and ‘the need for pain relief’. The resulting 31 candidate items meeting consensus in both stakeholder groups were grouped to form six outcomes for the COS: pain; disease activity; impact on quality of life and daily activities; patient satisfaction; treatment-related outcome; and microbiology (Fig 4).

#### AOE diagnostic criteria

Fourteen candidate items for the diagnostic criteria for AOE were evaluated by the professional stakeholder group. In the first round, 13 criteria were presented: aural fullness; EAC erythema; EAC granulations; EAC oedema; generalised lethargy; hearing impairment; ear itchiness; jaw pain; microbiological identification of organism; odour related to the ear; otalgia; otorrhoea; and tragal tenderness. Following stakeholder feedback, squamous debris was added as a candidate item into round 2.

**Fig 5:**
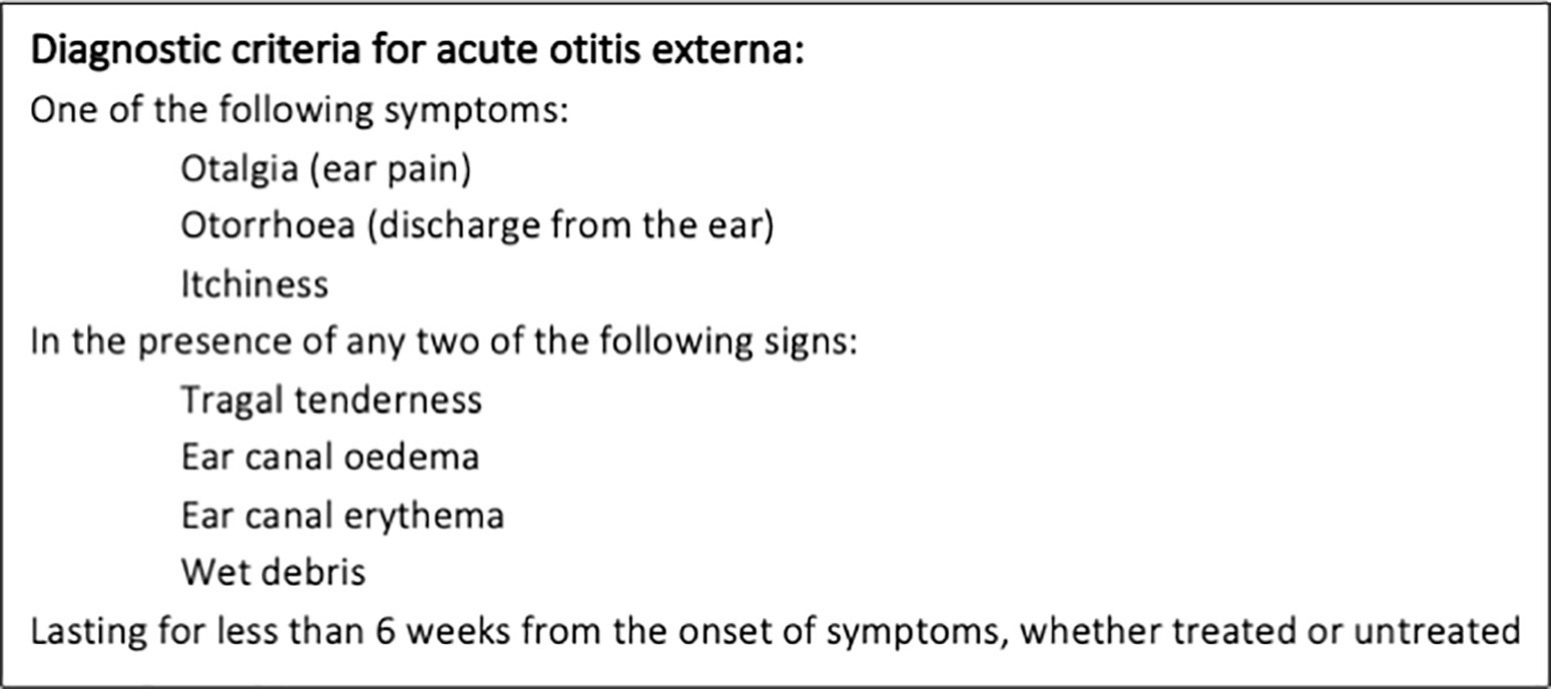
Consensus minimum diagnostic criteria for AOE.

The Delphi results for diagnostic criteria are shown in S3 Table, with three criteria meeting inclusion consensus in round 1, three more in round 2 and none in round 3. Only one criterion met exclusion consensus. Of the diagnostic criteria failing to reach consensus in the Delphi process, only ‘wet debris’ was additionally included as it was very close to the predefined consensus standard and, as a stakeholder-derived addition, was only presented over two rounds. The steering committee agreed diagnostic criteria by consensus (Fig 5).

#### AOE definition

Professional stakeholders were asked the timepoint beyond which otitis externa should no longer be considered acute. In the first round, two cut-off timepoints for acute disease were prominent with 40% of professionals indicating a preference for 6 weeks, and 28% indicating 12 weeks. In round 2, 60% selected the 6-week cut-off. In the final round, 70% supported 6 weeks to delineate acute from chronic otitis externa, which reached the threshold for consensus agreement.

Based on the stakeholder consensus diagnostic criteria and disease duration, six steering committee members independently formulated short definition statements for AOE. These were analysed for common features and a final definition was reached by consensus of the steering committee:

‘**Acute otitis externa is diffuse inflammation of the ear canal skin of less than 6 weeks duration**’

## Discussion

The characteristics of patients with AOE in interventional trials are poorly reported and vary between studies [7]. Previous attempts have been made to define the condition and set diagnostic criteria [8], however our work is novel by establishing these criteria via a three stage Delphi consensus process amongst professional stakeholders. Of note the 6 week period over which OE can be considered acute is longer than many trials have adopted [7], but stakeholder feedback suggested this was necessary and clinically relevant due to the delays in presentation and adequate treatment frequently seen with AOE.

The consensus diagnostic criteria for AOE have been developed primarily for use in interventional trials, to ensure consistency in inclusion criteria and to aid reporting, but may benefit observational studies, databases and clinical practice. Hearing loss and jaw pain feature in many previous diagnostic criteria [8], but were found not to rank highly with stakeholders, possibly due to their poor specificity for AOE. Similarly, the microbiological identification of an organism was not considered key to diagnosis by stakeholders. Much AOE is treated effectively in primary care [8, 9], without the cost and complexity associated with processing a microbiological sample, and it was thought by stakeholders that requirement for a confirmed organism within the criteria would delay formal diagnosis.

For interventional studies to be relevant to clinical practice and policy makers, the reported outcomes must be important to key stakeholders, most importantly patients with the condition and the healthcare professionals treating them. Furthermore these outcomes require widespread and consistent adoption to facilitate meta-analysis of outcomes, whereas to date, interventional studies in AOE have reported very varied outcomes [7].

Our work with former patients was central to developing candidate items for inclusion in the COS and this has led to the most significant differences between our proposed COS and the outcomes reported in previous work. Many of the items considered important by patients were not mirrored in those previously adopted in the literature, such as effect on daily activities and quality of life. These outcomes may have been overlooked by researchers in a condition seen as relatively minor, of short-duration and localised. The value of presenting the patient opinion to professionals could be seen in the Delphi responses, with former patients (but not clinicians) rating quality of life, effect on work and treatment satisfaction highly in the first round, and professionals then changing their responses to support inclusion of these in the second round. It has been noted that compliance with treatment for AOE is often poor and the reasons for this not yet explored [7]. The COS has the potential to identify interventions that patients find unappealing or difficult, which is important when considering treatment compliance on transfer to routine clinical practice.

Only approximately 3% of patients with AOE attending general practice in the UK need referral to an ENT specialist, and yet the vast majority of trials to date have been set in secondary care [7]. This may have affected the generalisability of outcomes presented in the literature, and it is important that the results of future studies conducted in primary and secondary care are comparable. Importantly this work recorded the views primary care physicians and the presented COS and diagnostic criteria are applicable to research in both primary and secondary care.

Developing a COS is the first step towards determining how we should measure the effectiveness of an intervention, defining only the type of outcome important to stakeholders, but not the specific metric or tool used to characterise each participant’s result [1]. As with the COS, outcome instrument selection and interpretation is best done via consensus of multiple stakeholder groups.

This work is the first to achieve wide stakeholder consensus, yet the comparatively small number of former patients completing the three rounds of Delphi represents a limitation. This might be partly mitigated by the fact that patient opinion was influential to professionals’ responses. The loss of former patient numbers participating between recruitment and completion of round 1 may be due to the length of the online Delphi, or a perceived lack of benefit from participation. Further, the severity of AOE experienced by the former patients who participated in the project was not controlled, and so a disproportionate representation may have biased the patient input towards severe of mild forms of the disease. Finally, this work was limited to adults as it was thought unlikely that a COS could be developed to reflect the key outcomes in both children and adults.

Involvement of former patients throughout the project has highlighted areas where current clinical practice may be improved, specifically in the delay to appropriate management and the control of pain. Better patient education may reduce the stress and anxiety associated with AOE, which has perhaps been underappreciated to date. Patient-derived priorities for research also did not fully align with those typically addressed. While most research to date has focussed on resolution of infection and inflammation, improving symptom control and the ease of use and tolerability of treatments were important to patients. This reinforces the increasingly acknowledged role of patient and public involvement in research agenda setting and study design.

## Conclusion

A COS for AOE has been developed and a definition and diagnostic criteria agreed. AOE should be defined as a condition of less than six weeks duration, and diagnosed via a combination of at least one characteristic symptom and two signs.

A stakeholder consensus process has highlighted deficiencies in the outcomes used for AOE in previous studies, in particular concerning its impact on daily life. The identification and/or development of tools to help implement the COS is now a priority.

## Supporting information

S1 Fig Example literature search strategy. (JPG)

S1 File Example professional stakeholder participant form for the Delphi process. This combined form shows the 3 rounds.
(PDF)

S1 Table Included articles and AOE definitions, diagnostic criteria and outcomes extracted from each. (XLSX)

S2 Table Delphi output from the professional and patient groups for outcomes. White = no consensus, grey = consensus (+ indicates to include,—to exclude), black = omitted from round having met consensus.
(PDF)

S3 Table Delphi output from the professional group for diagnostic criteria. *based on stakeholder feedback this criterion was adapted to ‘wet debris’. White = no consensus, grey = consensus (+ indicates to include,—to exclude), black = omitted from round having met consensus.
(PDF)

S1 Data Full dataset from the 3 Delphi rounds. (XLSX)
