# Supplementary figures and images for: Acute otitis externa: Consensus definition, diagnostic criteria and core outcome set development
Source: PLoS One. 2021 May 14;16(5):e0251395. doi: 10.1371/journal.pone.0251395 (PMC8121300; doi:10.1371/journal.pone.0251395)

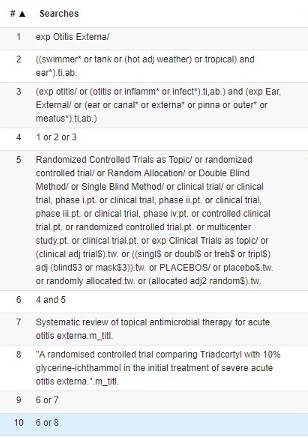

Supplement: S1 Fig — (JPG) [file pone.0251395.s001.jpg]
