# Supplementary material for: Acute otitis externa: Consensus definition, diagnostic criteria and core outcome set development
Source: PLoS One. 2021 May 14;16(5):e0251395. doi: 10.1371/journal.pone.0251395 (PMC8121300; doi:10.1371/journal.pone.0251395)
Supplement: S1 File — This combined form shows the 3 rounds. (PDF) [file pone.0251395.s002.pdf]

The DECODE Project: Delphi round 1

*Required

1. Email address *

Your email address is used to track your participation and allow you to review your responses. By entering your email you agree to the DECODE team using it only for the purposes of conducting this survey and communicating the results to you. You can withdraw your consent at anytime.

DEveloping consensus Core Outcomes and Diagnostic criteria for acute otitis Externa

Welcome

Thank you for participating as a stakeholder in the first round of a Delphi process to develop a diagnostic criteria and core outcome set (COS) for research into acute otitis externa.

Your answers to these survey questions are anonymous to everyone outside of the project steering committee, so please answer freely.

Participation in all three stages of the Delphi will mean you are recognised as a collaborator (identifiable on PubMed) in any subsequent presentations and publications resulting from this work.

You may have been provided with a Participant Information Sheet. A copy of this, should you wish to read it, may be found here: https://ENTintegrate.org/decode. Here you will find additional information in Frequently Asked Questions about how this Delphi process was developed, and also information on what will happen once you have completed this questionnaire.

Rationale

Research into otitis externa is heterogenous, with researchers using a wide variety of different diagnostic criteria and outcomes. This makes direct comparisons between previous studies difficult.

Our project aims to develop diagnostic criteria and a core outcome set for otitis externa to help standardise future research. We aim to survey key patient and professional stakeholders in an attempt to reach consensus.

Survey methods

For the diagnosis, you will be asked which features you think are important to be included in a minimum diagnostic criteria.

For the Core Outcome Set, the Delphi process involves answering questions on three separate occasions to gather your opinions relating to the importance of different outcome measures for otitis externa:

- ROUND 1: You will be asked to rate all the possible outcomes and suggest any additional outcomes you think may be important.

- ROUND 2: You will be shown summaries of how YOU AND YOUR PEERS responded to items that did not reach consensus in round 1. You will be asked to rate these items again in light of this information.

- ROUND 3: You will be shown summaries of how EVERYONE responded to items that did not reach consensus in round 2. You will be asked to rate these items again in light of this information.

The identity of all panelists will remain confidential at all times.

Final consensus

After these 3 rounds, the outcomes reaching consensus (using criteria explained in the protocol) will either be included or excluded from the final Core Outcome Set. Outcomes not reaching a consensus will be reviewed by the DECODE steering committee and a final consensus will be reached as to whether the item should be included or not, based on the information from the Delphi process from each stakeholder group. The resulting Core Outcome Set will be submitted for publication in a peer reviewed journal.

Thank you

Thank you once again for taking part. If you have any problems at any stage, please email info@entintegrate.org

INTEGRATE DECODE steering committee

2. Please select you stakeholder group *

Mark only one oval.

- Audiologist
- ENT registrar
- ENT consultant - Otologist
- ENT consultant - General / Non-otology
- GP
- Junior doctor (below ST3)
- Microbiologist
- Nurse

Section 1: Diagnosis of acute otitis externa

There are currently no accepted diagnostic criteria for acute otitis externa (AOE), which complicates patient selection for trials. For the purposes of these diagnostic criteria, localised infection in the external auditory canal (EAC)(e.g. furuncles) is excluded.

Firstly, We would like to work out when otitis externa changes from being 'acute' to being 'chronic'.

3. Otitis externa should be considered as 'chronic' after... *

Mark only one oval.

- 2 weeks
- 3 weeks
- 4 weeks
- 5 weeks
- 6 weeks
- 7 weeks
- 8 weeks
- 9 weeks
- 10 weeks
- 11 weeks
- 12 weeks

4. Comments

Signs and symptoms

Secondly, we'd like to establish the key features in the diagnosis of acute otitis externa. In a future round you will be able to state if these are essential criteria in all cases.

Please rate the importance of including the following features in the minimum DIAGNOSTIC criteria for acute otitis externa [13 items]

5. Aural fullness

Mark only one oval.

	1	2	3	4	5	6	7	8	9	
Lowest importance										Highest importance

6. Unable to score/comments

7. External auditory canal erythema

Mark only one oval.

	1	2	3	4	5	6	7	8	9	
Lowest importance										Highest importance

8. Unable to score/comments

9. External auditory canal granulations

Mark only one oval.

	1	2	3	4	5	6	7	8	9	
Lowest importance										Highest importance

10. Unable to score/comments

11. External auditory canal oedema

Mark only one oval.

	1	2	3	4	5	6	7	8	9	
Lowest importance										Highest importance

12. Unable to score/comments

13. Generalised lethargy

Mark only one oval.

	1	2	3	4	5	6	7	8	9	
Lowest importance										Highest importance

14. Unable to score/comments

15. Hearing impairment

Mark only one oval.

	1	2	3	4	5	6	7	8	9	
Lowest importance	( )	( )	( )	( )	( )	( )	( )	( )	( )	Highest importance

16. Unable to score/comments

17. Itchiness

Mark only one oval.

	1	2	3	4	5	6	7	8	9	
Lowest importance	( )	( )	( )	( )	( )	( )	( )	( )	( )	Highest importance

18. Unable to score/comments

19. Jaw pain

Mark only one oval.

	1	2	3	4	5	6	7	8	9	
Lowest importance	( )	( )	( )	( )	( )	( )	( )	( )	( )	Highest importance

20. Unable to score/comments

21. Microbiological identification of an organism

Mark only one oval.

	1	2	3	4	5	6	7	8	9	
Lowest importance										Highest importance

22. Unable to score/comments

23. Odour related to the ear

Mark only one oval.

	1	2	3	4	5	6	7	8	9	
Lowest importance										Highest importance

24. Unable to score/comments

25. Otalgia (ear pain)

Mark only one oval.

	1	2	3	4	5	6	7	8	9	
Lowest importance	◯	◯	◯	◯	◯	◯	◯	◯	◯	Highest importance

26. Unable to score/comments

27. Otorrhoea (discharge from the ear)

Mark only one oval.

	1	2	3	4	5	6	7	8	9	
Lowest importance	◯	◯	◯	◯	◯	◯	◯	◯	◯	Highest importance

28. Unable to score/comments

29. Tragal tenderness

Mark only one oval.

	1	2	3	4	5	6	7	8	9	
Lowest importance	◯	◯	◯	◯	◯	◯	◯	◯	◯	Highest importance

30. Unable to score/comments

31. Please state any other symptoms or signs that you think should be included in diagnostic criteria for otitis externa

Section 2: Core outcome set for acute otitis externa

Currently there is no accepted set of outcome measures used in studies of patients with acute otitis externa (AOE). This can lead to difficulty in analysing and interpreting clinical studies, which limits the effectiveness of our treatment of patients with AOE .

We'd like to establish which features of AOE should be included in a Core Outcome Set (COS) for use in all future studies of acute otitis externa (AOE).

The development of a Core Outcome Set (COS) first involves determining WHAT should be measured as an outcome, later establishing HOW this should be measured. The following questions hope to identify WHAT features should be measured.

In studies looking at acute otitis externa studies, which of these OUTCOMES are important to report? [42 items]

NOTE: This is different from the diagnosis section. This section asks: What OUTCOMES from the MANAGEMENT of acute otitis externa are important? ie what should studies tell us about if they're looking at different ways of managing acute otitis externa. Not how to diagnose acute otitis externa in the first place

[1-5] Studies should report...

32. ...on improvement in the ability to concentrate

Mark only one oval.

	1	2	3	4	5	6	7	8	9	
Lowest importance	○	○	○	○	○	○	○	○	○	Highest importance

33. Unable to score/comments

34. ...on results of bacterial or fungal growth on an ear swab

Mark only one oval.

	1	2	3	4	5	6	7	8	9	
Lowest importance	○	○	○	○	○	○	○	○	○	Highest importance

35. Unable to score/comments

36. ...if patients completed the course of treatment

Mark only one oval.

	1	2	3	4	5	6	7	8	9	
Lowest importance	○	○	○	○	○	○	○	○	○	Highest importance

37. Unable to score/comments

38. ...if patients were compliant with the treatment

Mark only one oval.

	1	2	3	4	5	6	7	8	9	
Lowest importance	◯	◯	◯	◯	◯	◯	◯	◯	◯	Highest importance

39. Unable to score/comments

40. ...if patients died during treatment

Mark only one oval.

	1	2	3	4	5	6	7	8	9	
Lowest importance	◯	◯	◯	◯	◯	◯	◯	◯	◯	Highest importance

41. Unable to score/comments

[6-10] Studies should report...

42. ...on improvements in discharge from the ear (otorrhoea)

Mark only one oval.

	1	2	3	4	5	6	7	8	9	
Lowest importance	◯	◯	◯	◯	◯	◯	◯	◯	◯	Highest importance

43. Unable to score/comments

44. ...on improvements in reactive over-healing in the ear canal (called granulations)

Mark only one oval.

	1	2	3	4	5	6	7	8	9	
Lowest importance	◯	◯	◯	◯	◯	◯	◯	◯	◯	Highest importance

45. Unable to score/comments

46. …on improvements in ear canal redness (erythema)

Mark only one oval.

	1	2	3	4	5	6	7	8	9	
Lowest importance	◯	◯	◯	◯	◯	◯	◯	◯	◯	Highest importance

47. Unable to score/comments

48. …on improvements in ear canal swelling (oedema)

Mark only one oval.

	1	2	3	4	5	6	7	8	9	
Lowest importance	◯	◯	◯	◯	◯	◯	◯	◯	◯	Highest importance

49. Unable to score/comments

50. …on improvements in ear pain (otalgia)

Mark only one oval.

	1	2	3	4	5	6	7	8	9	
Lowest importance	◯	◯	◯	◯	◯	◯	◯	◯	◯	Highest importance

51. Unable to score/comments

____

____

____

____

____

[11-15] Studies should report...

52. ...on the ease of applying the treatment

Mark only one oval.

	1	2	3	4	5	6	7	8	9	
Lowest importance	⬭	⬭	⬭	⬭	⬭	⬭	⬭	⬭	⬭	Highest importance

53. Unable to score/comments

____

____

____

____

____

54. ...on any impact on activities of daily living or performing household task

Mark only one oval.

	1	2	3	4	5	6	7	8	9	
Lowest importance	⬭	⬭	⬭	⬭	⬭	⬭	⬭	⬭	⬭	Highest importance

55. Unable to score/comments

56. ...on any impact on ability to care for the family

Mark only one oval.

	1	2	3	4	5	6	7	8	9	
Lowest importance										Highest importance

57. Unable to score/comments

58. ...on any impact on mental health

Mark only one oval.

	1	2	3	4	5	6	7	8	9	
Lowest importance										Highest importance

59. Unable to score/comments

60. ...on any impact on sleep

Mark only one oval.

	1	2	3	4	5	6	7	8	9	
Lowest importance	( )	( )	( )	( )	( )	( )	( )	( )	( )	Highest importance

61. Unable to score/comments

____
____
____
____
____

[16-20] Studies should report...

62. ...on any impact on the ability to socialise

Mark only one oval.

	1	2	3	4	5	6	7	8	9	
Lowest importance	( )	( )	( )	( )	( )	( )	( )	( )	( )	Highest importance

63. Unable to score/comments

____
____
____
____
____

64. ...on any impact on the ability to work

Mark only one oval.

	1	2	3	4	5	6	7	8	9	
Lowest importance										Highest importance

65. Unable to score/comments

66. ...on improvements in a feeling of fullness in the ear (aural fullness)

Mark only one oval.

	1	2	3	4	5	6	7	8	9	
Lowest importance										Highest importance

67. Unable to score/comments

68. ...on improvements in a feeling of generalised weakness

Mark only one oval.

	1	2	3	4	5	6	7	8	9	
Lowest importance										Highest importance

69. Unable to score/comments

70. ...on improvements in hearing impairment

Mark only one oval.

	1	2	3	4	5	6	7	8	9	
Lowest importance	○	○	○	○	○	○	○	○	○	Highest importance

71. Unable to score/comments

[21-25] Studies should report...

72. ...on any impact on hours of bed rest

Mark only one oval.

	1	2	3	4	5	6	7	8	9	
Lowest importance	○	○	○	○	○	○	○	○	○	Highest importance

73. Unable to score/comments

74. ...on improvements in itchiness

Mark only one oval.

	1	2	3	4	5	6	7	8	9	
Lowest importance										Highest importance

75. Unable to score/comments

76. ...on improvements in jaw pain

Mark only one oval.

	1	2	3	4	5	6	7	8	9	
Lowest importance										Highest importance

77. Unable to score/comments

78. ...on improvements in the feeling of anxiety

Mark only one oval.

	1	2	3	4	5	6	7	8	9	
Lowest importance										Highest importance

79. Unable to score/comments

80. ...on any local side effects of treatments

Mark only one oval.

	1	2	3	4	5	6	7	8	9	
Lowest importance										Highest importance

81. Unable to score/comments

[26-30] Studies should report...

82. ...on the need for antibiotics

Mark only one oval.

	1	2	3	4	5	6	7	8	9	
Lowest importance	◯	◯	◯	◯	◯	◯	◯	◯	◯	Highest importance

83. Unable to score/comments

____
____
____
____
____

84. ...on the need for overnight or in-hospital care

Mark only one oval.

	1	2	3	4	5	6	7	8	9	
Lowest importance	◯	◯	◯	◯	◯	◯	◯	◯	◯	Highest importance

85. Unable to score/comments

____
____
____
____
____

86. ...on the need for pain relief

Mark only one oval.

	1	2	3	4	5	6	7	8	9	
Lowest importance	◯	◯	◯	◯	◯	◯	◯	◯	◯	Highest importance

87. Unable to score/comments

88. ...on the number/frequency of visits to ENT or the GP

Mark only one oval.

	1	2	3	4	5	6	7	8	9	
Lowest importance										Highest importance

89. Unable to score/comments

90. ... on the number of times the treatment is administered

Mark only one oval.

	1	2	3	4	5	6	7	8	9	
Lowest importance										Highest importance

91. Unable to score/comments

[31-35] Studies should report...

92. ...on improvements in any odour relating to the ear

Mark only one oval.

	1	2	3	4	5	6	7	8	9	
Lowest importance										Highest importance

93. Unable to score/comments

94. ...on improvements in any pain on pressing just in front of the ear canal (tragal tenderness)

Mark only one oval.

	1	2	3	4	5	6	7	8	9	
Lowest importance										Highest importance

95. Unable to score/comments

96. ...on any impact on quality of life

Mark only one oval.

	1	2	3	4	5	6	7	8	9	
Lowest importance	◯	◯	◯	◯	◯	◯	◯	◯	◯	Highest importance

97. Unable to score/comments

98. ...on patient satisfaction with their access to appropriate care (for diagnosis & treatment)

Mark only one oval.

	1	2	3	4	5	6	7	8	9	
Lowest importance	◯	◯	◯	◯	◯	◯	◯	◯	◯	Highest importance

99. Unable to score/comments

100. ...on patient satisfaction with the length of their symptoms

Mark only one oval.

	1	2	3	4	5	6	7	8	9	
Lowest importance	◯	◯	◯	◯	◯	◯	◯	◯	◯	Highest importance

101. Unable to score/comments

[36-40] Studies should report...

102. ...on patient satisfaction with length of treatment, including number of visits to specialist services (ENT)

Mark only one oval.

	1	2	3	4	5	6	7	8	9	
Lowest importance										Highest importance

103. Unable to score/comments

104. ...on patient satisfaction with the required frequency of the treatments

Mark only one oval.

	1	2	3	4	5	6	7	8	9	
Lowest importance										Highest importance

105. Unable to score/comments

106. ...on patient satisfaction with the time required for outpatient appointments

Mark only one oval.

	1	2	3	4	5	6	7	8	9	
Lowest importance										Highest importance

107. Unable to score/comments

108. ...on any spread of infection beyond the ear canal

Mark only one oval.

	1	2	3	4	5	6	7	8	9	
Lowest importance										Highest importance

109. Unable to score/comments

110. ...on any time off work

Mark only one oval.

	1	2	3	4	5	6	7	8	9	
Lowest importance	⬭	⬭	⬭	⬭	⬭	⬭	⬭	⬭	⬭	Highest importance

111. Unable to score/comments

____
____
____
____
____

[41-42] Studies should report...

112. ...on any widespread (systemic) side effects of pain relief

Mark only one oval.

	1	2	3	4	5	6	7	8	9	
Lowest importance	⬭	⬭	⬭	⬭	⬭	⬭	⬭	⬭	⬭	Highest importance

113. Unable to score/comments

____
____
____
____
____

114. ...on any widespread (systemic) side effects of treatments

Mark only one oval.

	1	2	3	4	5	6	7	8	9	
Lowest importance	◯	◯	◯	◯	◯	◯	◯	◯	◯	Highest importance

115. Unable to score/comments

And finally...

116. Please state any other outcomes that you think studies should include when reporting on the management of acute otitis externa

*** THANK YOU ***

That's it!

Thank you for taking part. Your responses will help create the diagnostic criteria and a core outcome set for acute otitis externa. This is round 1 of the process. We'll give you the feedback from everyone's responses to this round, then ask if this influences your answers for the next round, to reach a consensus.

Once you're happy with your responses, please hit submit to record them.

Thank you once again for taking part. If you have any problems at any stage, please email info@entintegrate.org

INTEGRATE DECODE steering committee

This content is neither created nor endorsed by Google.

The DECODE Project: Delphi round 2

*Required

1. Email address *

Your email address is used to track your participation and allow you to review your responses. By entering your email you agree to the DECODE team using it only for the purposes of conducting this survey and communicating the results to you. You can withdraw your consent at anytime.

DEveloping consensus Core Outcomes and Diagnostic criteria for acute otitis Externa

Welcome to round 2

Thank you for participating as a stakeholder in the second round of a Delphi process to develop a diagnostic criteria and core outcome set (COS) for research into acute otitis externa.

Your answers to these survey questions are anonymous to everyone outside of the project steering committee, so please answer freely.

Participation in all three stages of the Delphi will mean you are recognised as a collaborator (identifiable on PubMed) in any subsequent presentations and publications resulting from this work.

You may have been provided with a Participant Information Sheet. A copy of this, should you wish to read it, may be found here: https://ENTintegrate.org/decode. Here you will find additional information in Frequently Asked Questions about how this Delphi process was developed, and also information on what will happen once you have completed this questionnaire.

Rationale

Research into otitis externa is heterogenous, with researchers using a wide variety of different diagnostic criteria and outcomes. This makes direct comparisons between previous studies difficult.

Our project aims to develop diagnostic criteria and a core outcome set for otitis externa to help standardise future research. We aim to survey key patient and professional stakeholders in an attempt to reach consensus.

Survey methods

For the diagnosis, you will be asked which features you think are important to be included in a minimum diagnostic criteria.

For the Core Outcome Set, the Delphi process involves answering questions on three separate occasions to gather your opinions relating to the importance of different outcome measures for otitis externa:

Completed:
- ROUND 1: You were asked to rate all the possible outcomes and suggest any additional outcomes you think may be important.

Now:
- ROUND 2: You will be shown summaries of how YOU AND YOUR PEERS responded to items that did not reach consensus in round 1. You will be asked to rate these items again in light of this information.

Later:
- ROUND 3: You will be shown summaries of how EVERYONE responded to items that did not reach consensus in round 2. You will be asked to rate these items again in light of this information.

The identity of all panelists will remain confidential at all times.

Final consensus

After these 3 rounds, the outcomes reaching consensus (using criteria explained in the protocol) will either be included or excluded from the final Core Outcome Set. Outcomes not reaching a consensus will be reviewed by the DECODE steering committee and a final consensus will be reached as to whether the item should be included or not, based on the information from the Delphi process from each stakeholder group. The resulting Core Outcome Set will be submitted for publication in a peer reviewed journal.

Thank you

Thank you once again for taking part. If you have any problems at any stage, please email info@entintegrate.org

INTEGRATE DECODE steering committee

2. Please select you stakeholder group *

Mark only one oval.

- ⬭ Audiologist
- ⬭ ENT registrar
- ⬭ ENT consultant - Otologist
- ⬭ ENT consultant - General / Non-otology
- ⬭ GP
- ⬭ Junior doctor (below ST3)
- ⬭ Microbiologist
- ⬭ Nurse

Changes to the survey

Thank you for all the comments you provided. These have all been reviewed by the steering committee. As a result of this feedback, the phrasing of some of the questions has been amended. We have also added new questions where it was felt the item was not previously covered. All your comments will be used to inform the final consensus process in instances where there remains disagreement after the 3 consultation rounds.

SECTION 1: Diagnosis of acute otitis externa

There are currently no accepted diagnostic criteria for acute otitis externa (AOE), which complicates patient selection for trials.

For the purposes of these diagnostic criteria:
- Super localised infections of the external ear (e.g. furuncles) are excluded.
- Uncomplicated otitis externa is considered infection without spread beyond the soft tissues of the external ear.
- Necrotising otitis externa/skull base osteomyelitis is considered a different entity

Results from round 1:

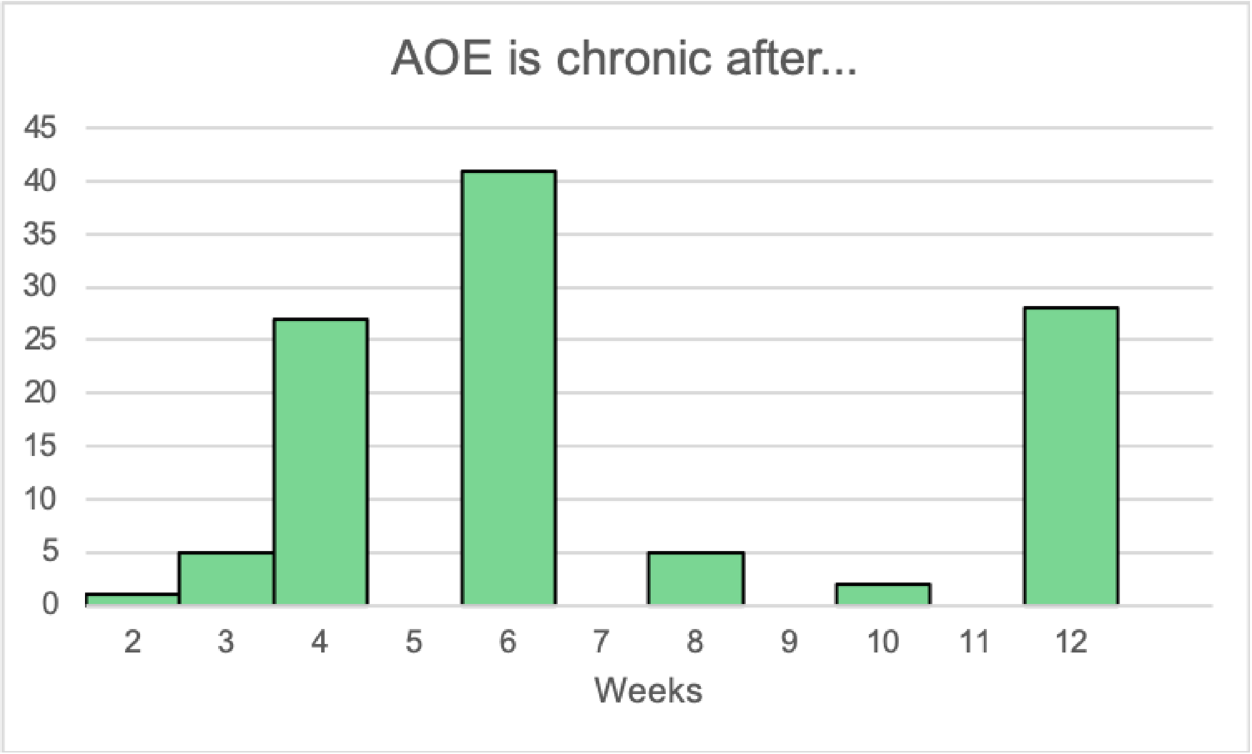

3. From the onset of symptoms, treated or untreated acute otitis externa should be considered to have become ‘chronic’ after... *

Mark only one oval.

- 2 weeks
- 3 weeks
- 4 weeks
- 5 weeks
- 6 weeks
- 7 weeks
- 8 weeks
- 9 weeks
- 10 weeks
- 11 weeks
- 12 weeks

4. Comments

SIGNS & SYMPTOMS

We'd like to establish the KEY FEATURES in the diagnosis of acute otitis externa.

Consensus results from round 1

3 of the 13 items from round 1 reached consensus amongst professionals to be included in the MINIMUM DIAGNOSTIC CRITERIA for AOE:

- External auditory canal oedema
- Otalgia (ear pain)
- Otorrhoea (discharge from the ear)

No items were excluded in round 1.

Based on your comments, 1 further item has been added.

- Squamous debris/keratin

Please rate the importance of including the following features in the MINIMUM DIAGNOSTIC CRITERIA for AOE: [10/13 items remaining, 1 new item]

Results from round 1:

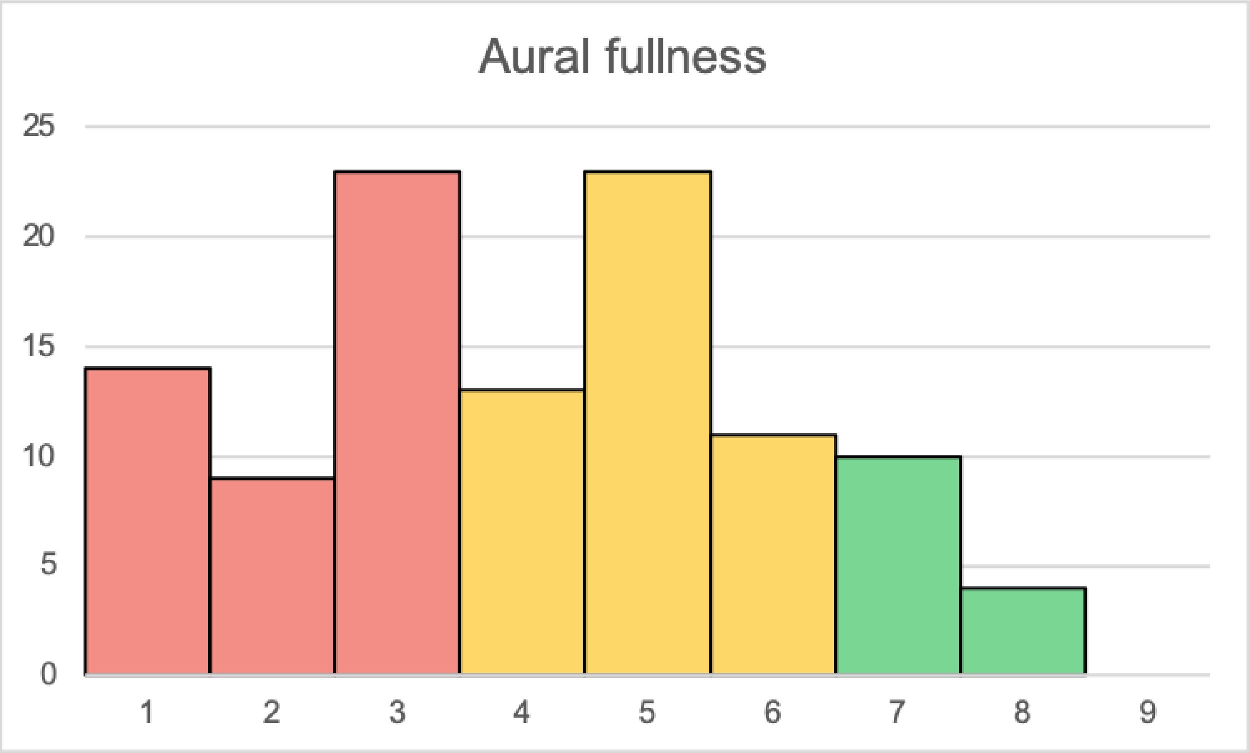

5. Aural fullness

Mark only one oval.

	1	2	3	4	5	6	7	8	9	
Lowest importance	◯	◯	◯	◯	◯	◯	◯	◯	◯	Highest importance

6. Unable to score/comments

Results from round 1:

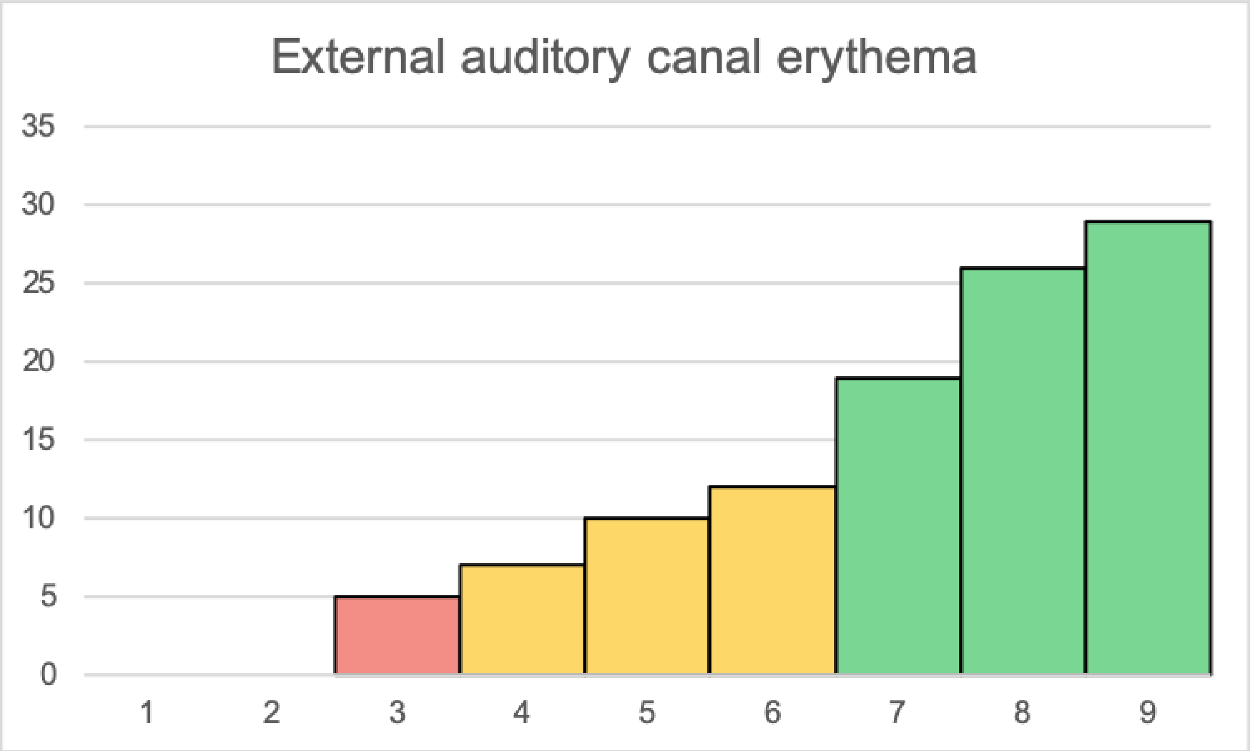

7. External auditory canal erythema

Mark only one oval.

	1	2	3	4	5	6	7	8	9	
Lowest importance	⬭	⬭	⬭	⬭	⬭	⬭	⬭	⬭	⬭	Highest importance

8. Unable to score/comments

Results from round 1:

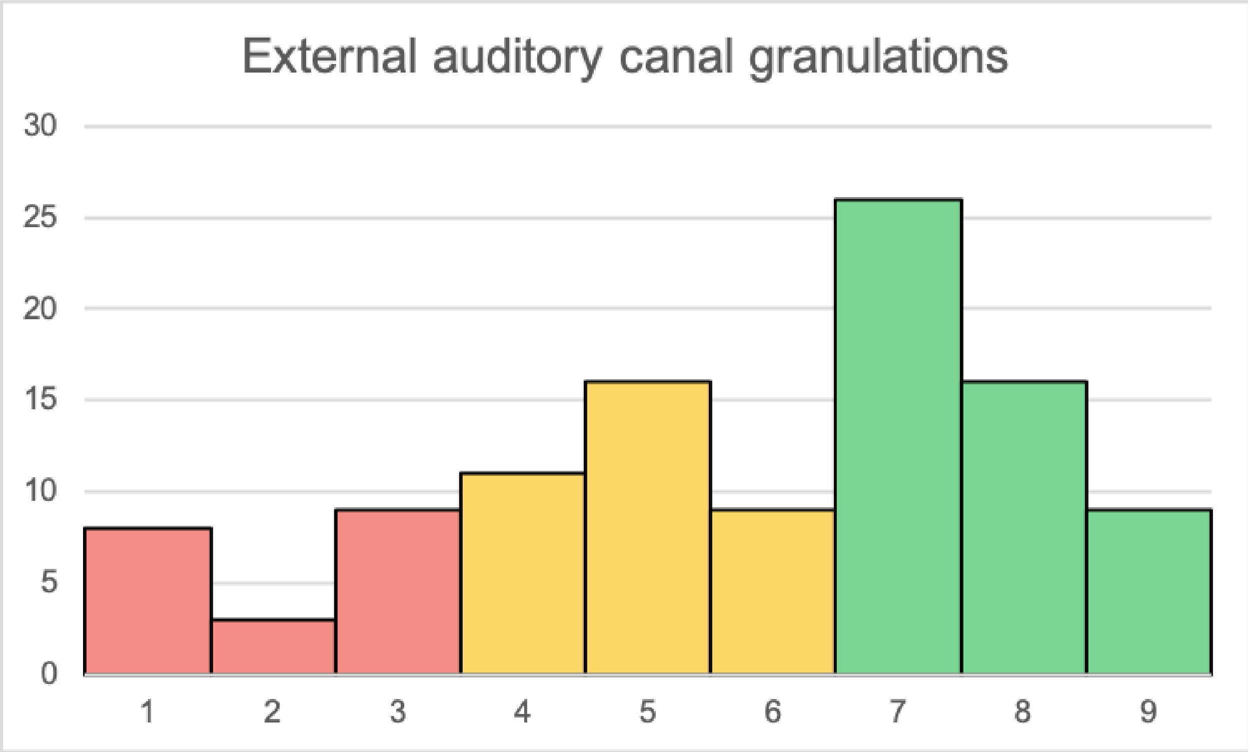

9. External auditory canal granulations

Mark only one oval.

	1	2	3	4	5	6	7	8	9	
Lowest importance	⬭	⬭	⬭	⬭	⬭	⬭	⬭	⬭	⬭	Highest importance

10. Unable to score/comments

Results from round 1:

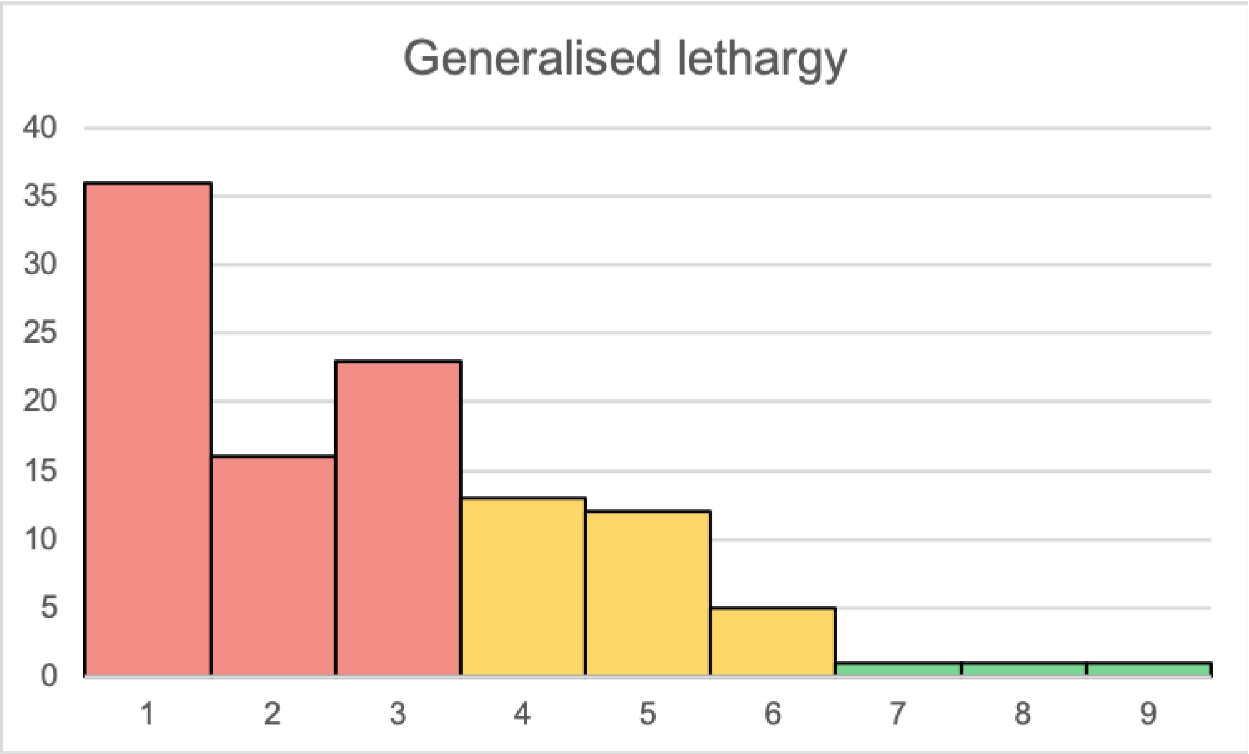

11. Generalised lethargy

Mark only one oval.

	1	2	3	4	5	6	7	8	9	
Lowest importance	◯	◯	◯	◯	◯	◯	◯	◯	◯	Highest importance

12. Unable to score/comments

Results from round 1:

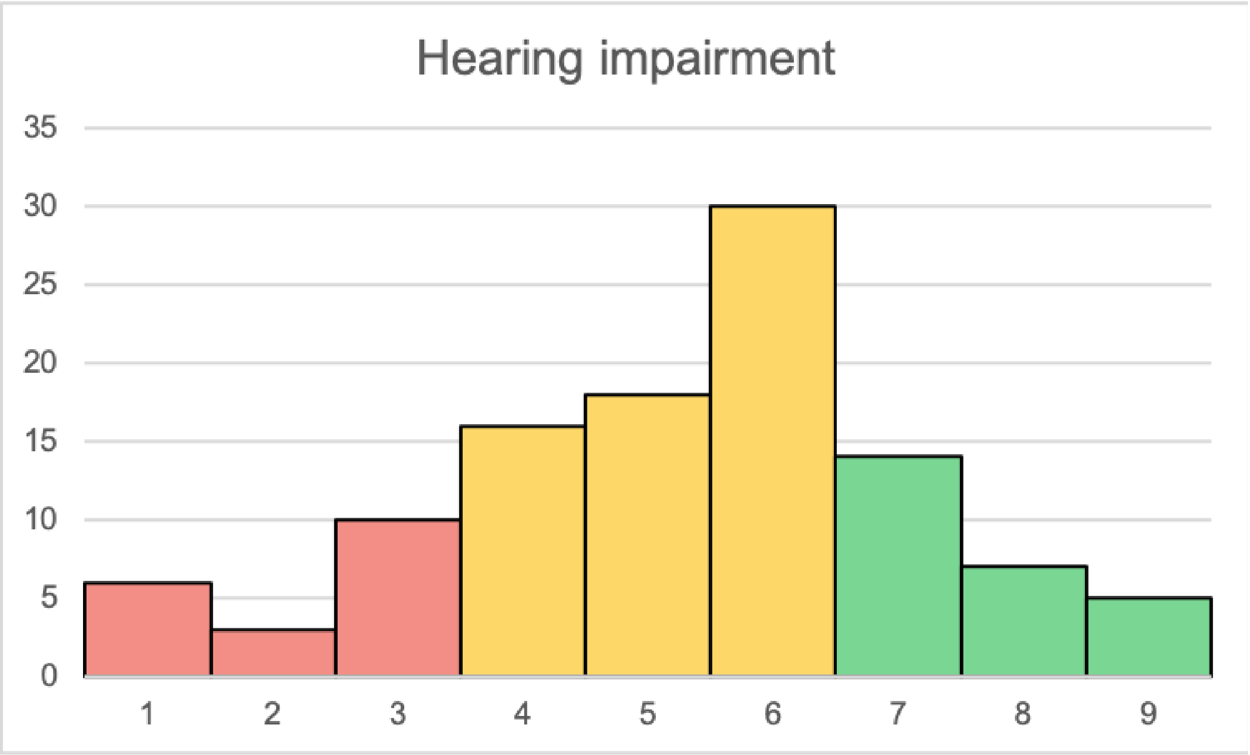

13. Hearing impairment

Mark only one oval.

	1	2	3	4	5	6	7	8	9	
Lowest importance	◯	◯	◯	◯	◯	◯	◯	◯	◯	Highest importance

14. Unable to score/comments

Results from round 1:

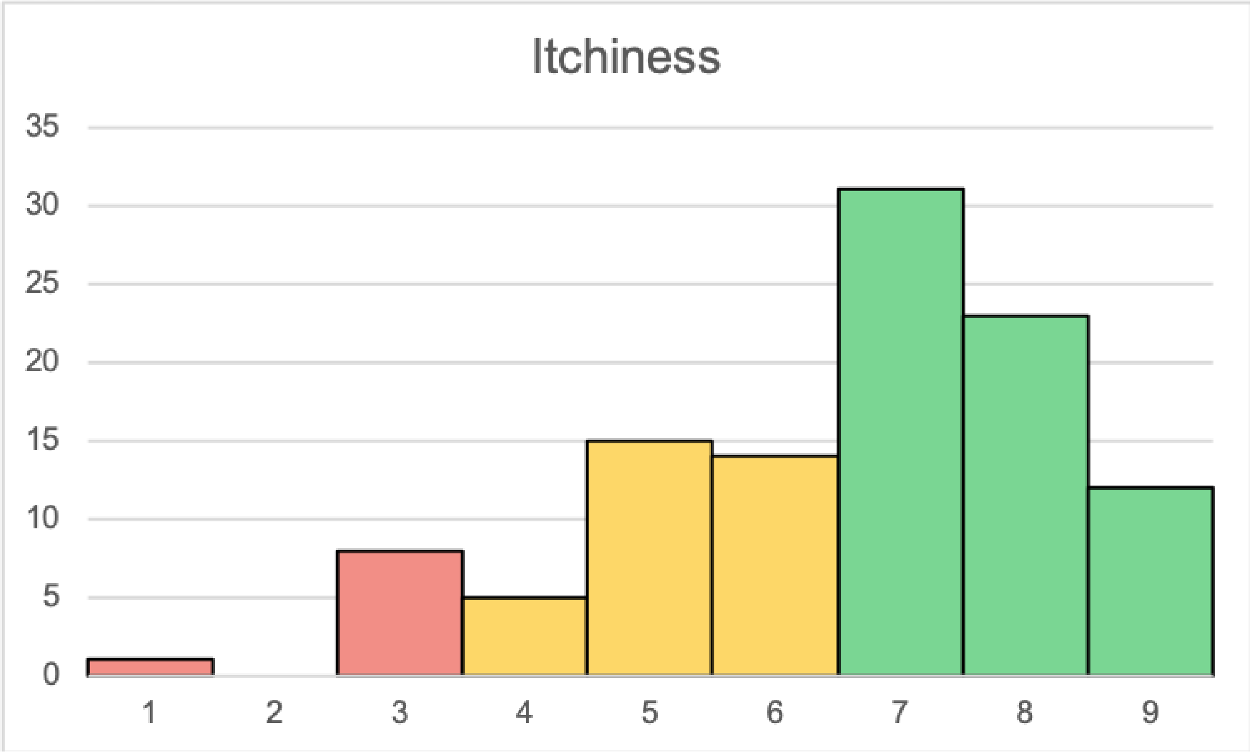

15. Itchiness

Mark only one oval.

	1	2	3	4	5	6	7	8	9	
Lowest importance	◯	◯	◯	◯	◯	◯	◯	◯	◯	Highest importance

16. Unable to score/comments

Results from round 1:

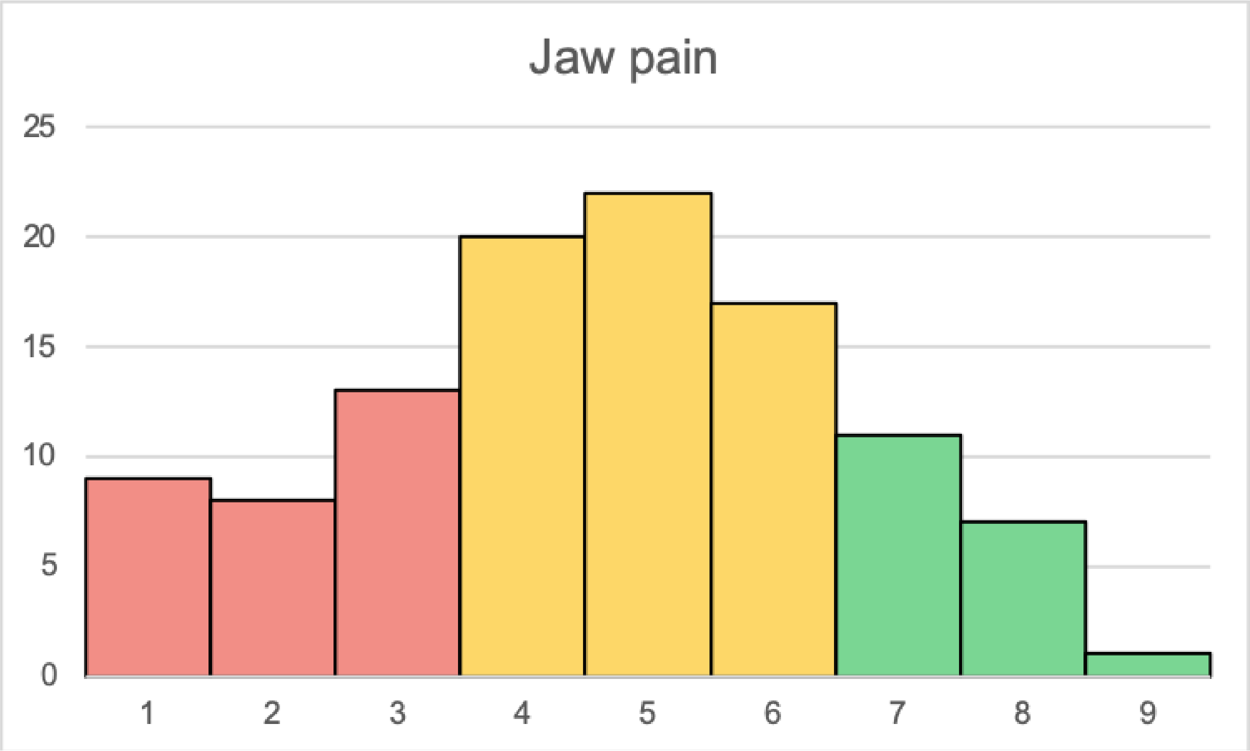

17. Jaw pain

Mark only one oval.

	1	2	3	4	5	6	7	8	9	
Lowest importance	◯	◯	◯	◯	◯	◯	◯	◯	◯	Highest importance

18. Unable to score/comments

Results from round 1:

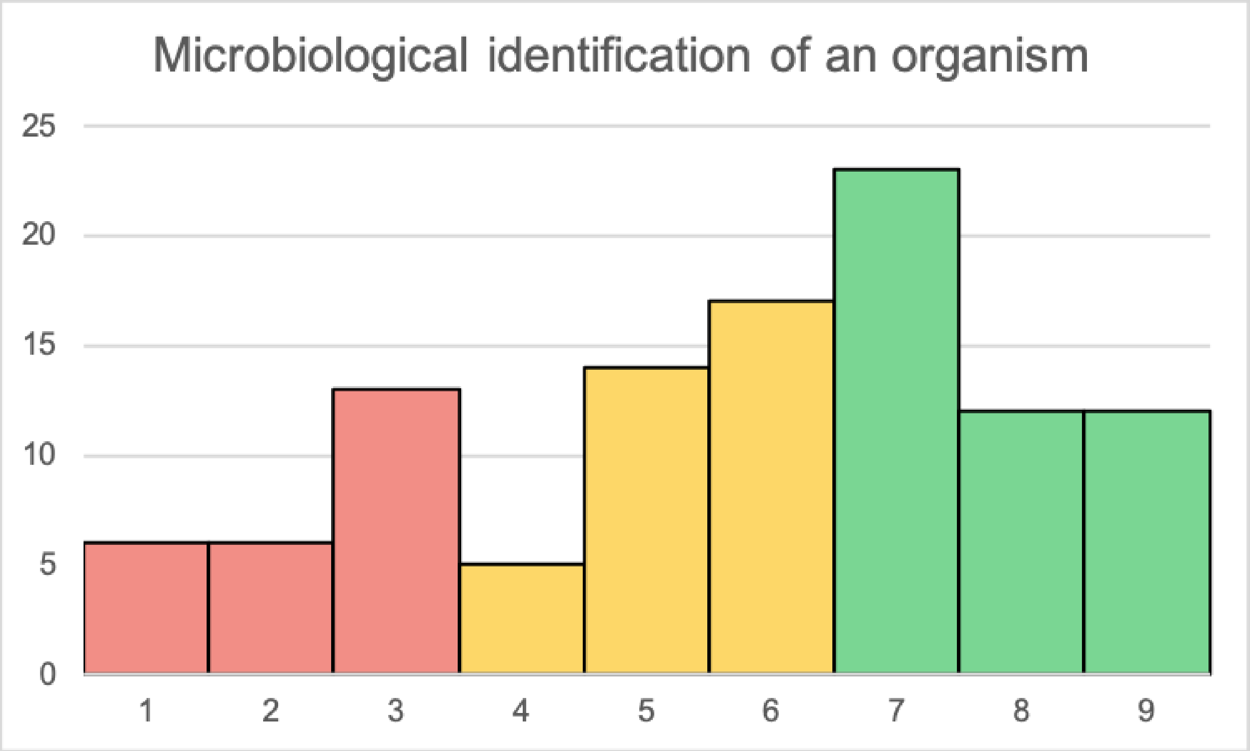

19. Microbiological identification of an organism

Mark only one oval.

	1	2	3	4	5	6	7	8	9	
Lowest importance	◯	◯	◯	◯	◯	◯	◯	◯	◯	Highest importance

20. Unable to score/comments

Results from round 1:

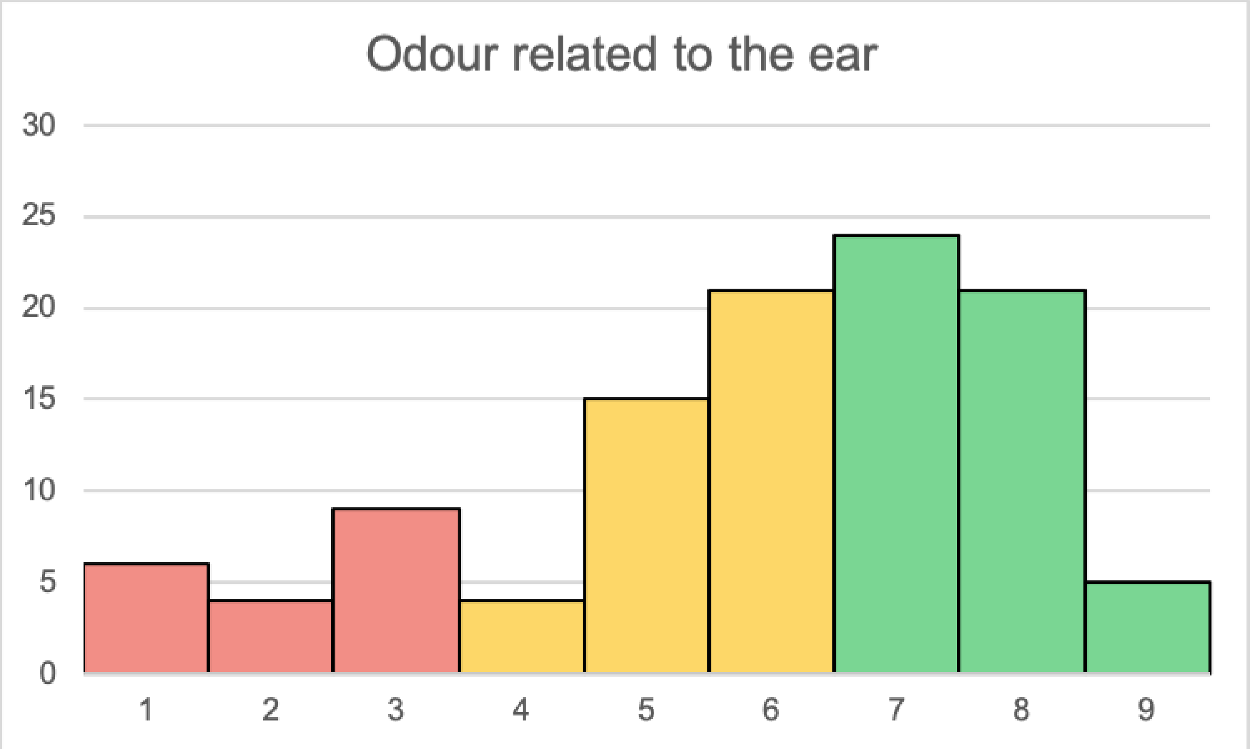

21. Odour related to the ear

Mark only one oval.

	1	2	3	4	5	6	7	8	9	
Lowest importance	◯	◯	◯	◯	◯	◯	◯	◯	◯	Highest importance

22. Unable to score/comments

Results from round 1:

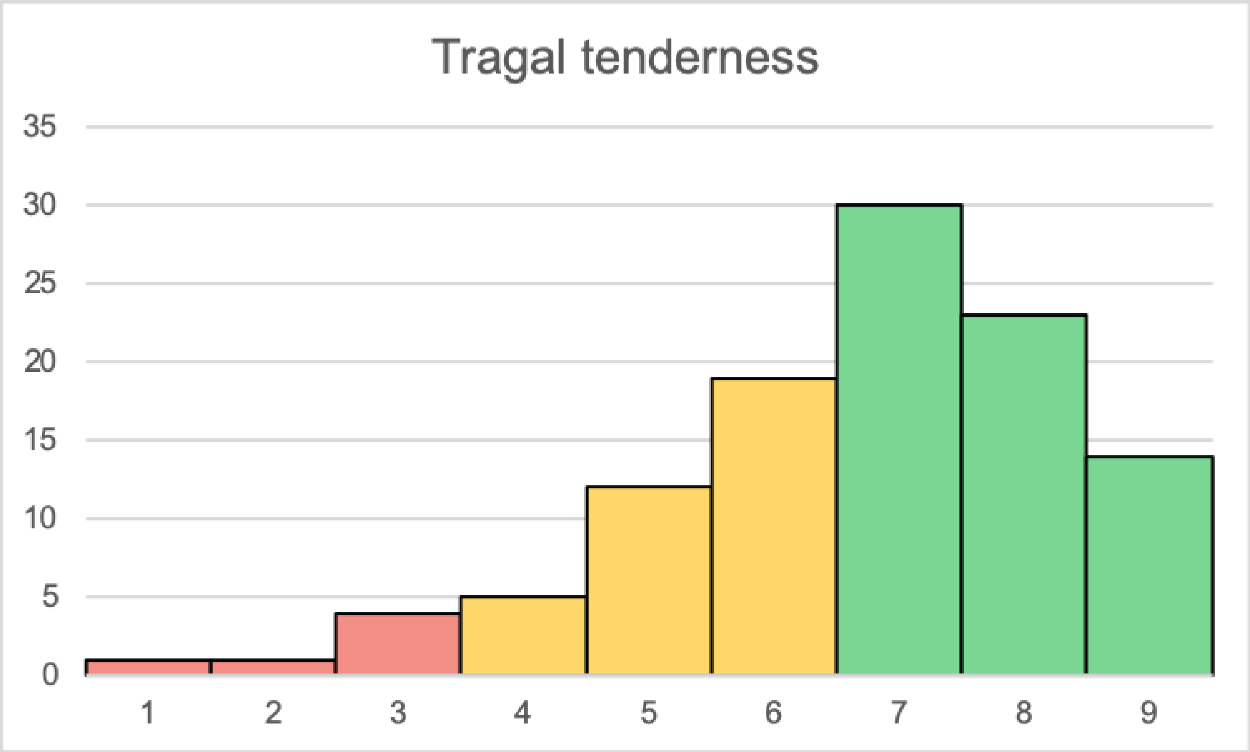

23. Tragal tenderness

Mark only one oval.

	1	2	3	4	5	6	7	8	9	
Lowest importance	⬭	⬭	⬭	⬭	⬭	⬭	⬭	⬭	⬭	Highest importance

24. Unable to score/comments

NEW ITEM:

25. Squamous debris/keratin

Mark only one oval.

	1	2	3	4	5	6	7	8	9	
Lowest importance	◯	◯	◯	◯	◯	◯	◯	◯	◯	Highest importance

26. Unable to score/comments

____

____

____

____

____

Section 2: Core outcome set for acute otitis externa

Currently there is no accepted set of outcome measures used in studies of patients with acute otitis externa (AOE). This can lead to difficulty in analysing and interpreting clinical studies, which limits the effectiveness of our treatment of patients with AOE .

We'd like to establish which features of AOE should be included in a Core Outcome Set (COS) for use in all future studies of acute otitis externa (AOE).

The development of a Core Outcome Set (COS) first involves determining WHAT should be measured as an outcome, later establishing HOW this should be measured. The following questions hope to identify WHAT features should be measured.

Consensus results from round 1

17 of the 42 items reached consensus amongst professionals to be included in the CORE OUTCOME SET for AOE.

...if patients completed the course of treatment
...if patients were compliant with the treatment
...on improvements in discharge from the ear (otorrhoea)
...on improvements in reactive over-healing in the ear canal (called granulations)
...on improvements in ear canal redness (erythema)
...on improvements in ear canal swelling (oedema)
...on improvements in ear pain (otalgia)
...on improvements in hearing impairment
...on improvements in itchiness
...on any local side effects of treatments
...on the need for antibiotics
...on the need for overnight or in-hospital care
...on the need for pain relief
...on the number/frequency of visits to ENT or the GP
...on the number of times the treatment is administered
...on any spread of infection beyond the ear canal
...on any widespread (systemic) side effects of treatments

No items were excluded in round 1.

Based on your feedback, 1 item has been added:

…the time to resolution of symptoms

These outcomes have NOT reached consensus. Please use the feedback from round 1 to FOCUS on which OUTCOMES you would like to EXCLUDE (or INCLUDE) in an ESSENTIAL outcome set, for trials in acute otitis externa

[1-5] Studies should report...

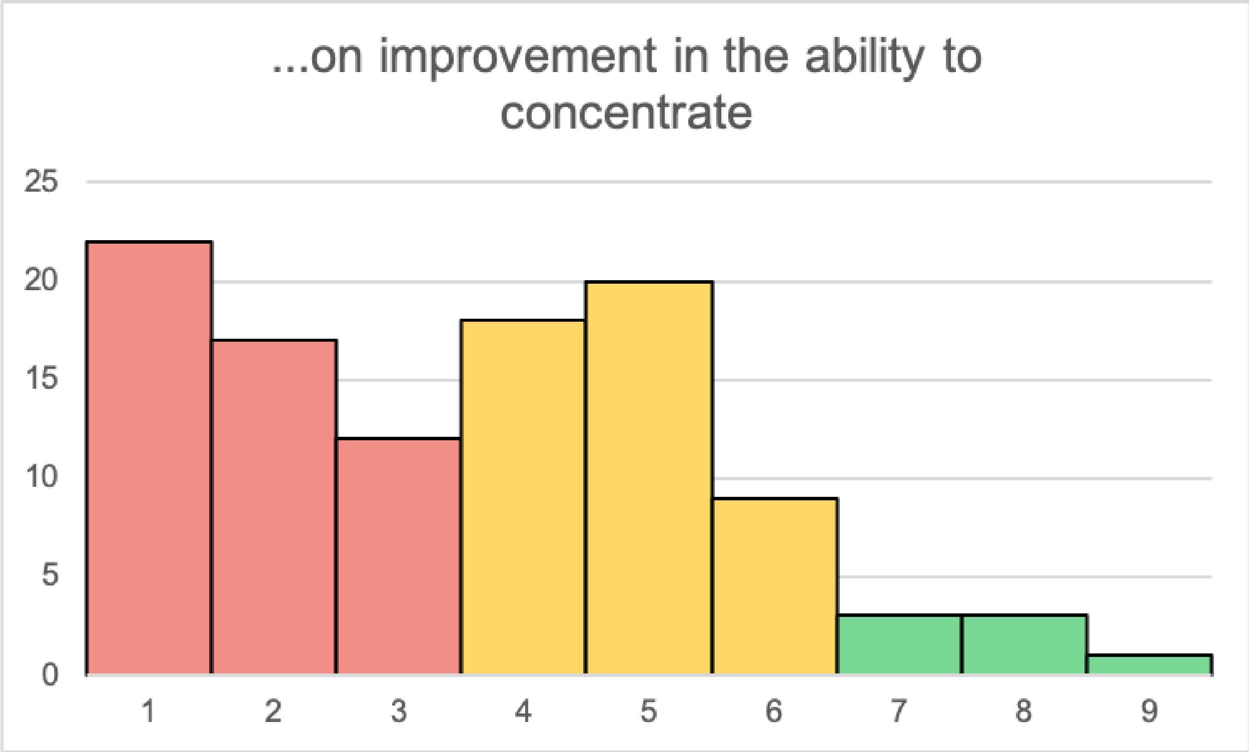

27. ...on improvement in the ability to concentrate

Mark only one oval.

	1	2	3	4	5	6	7	8	9	
Lowest importance	◯	◯	◯	◯	◯	◯	◯	◯	◯	Highest importance

28. Unable to score/comments

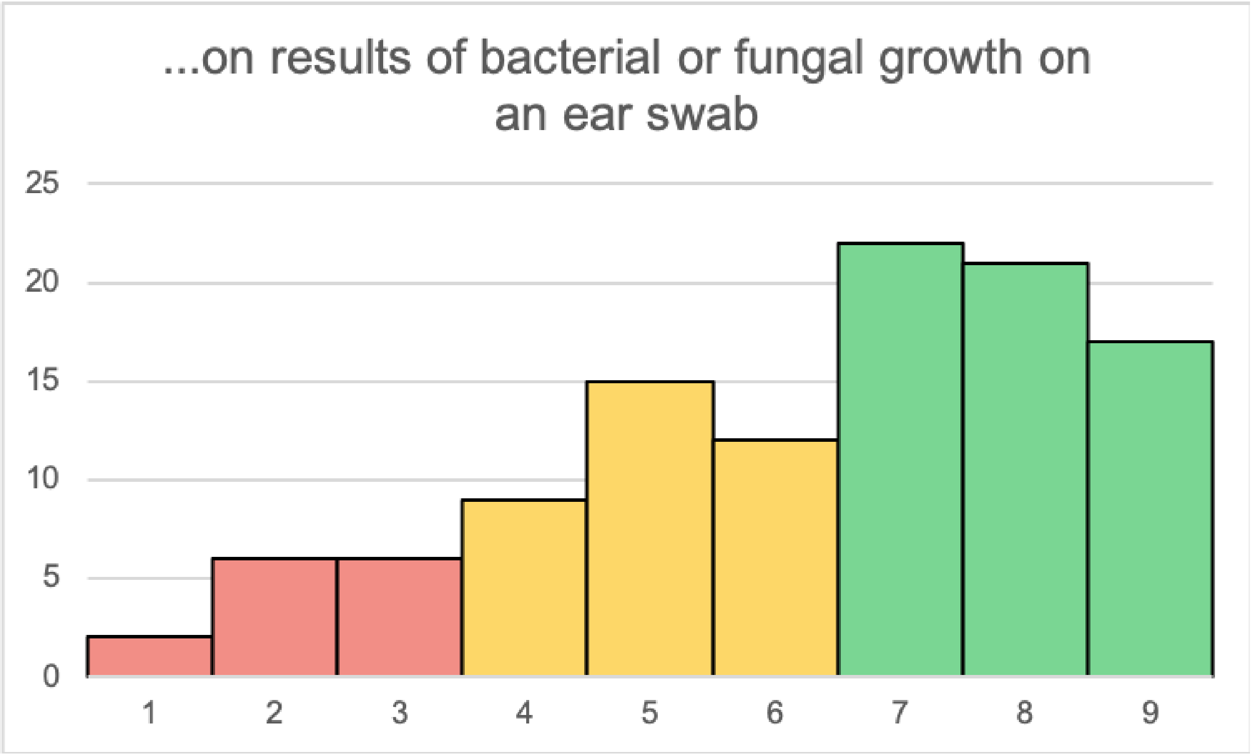

29. ...on results of bacterial or fungal growth on an ear swab

Mark only one oval.

	1	2	3	4	5	6	7	8	9	
Lowest importance	○	○	○	○	○	○	○	○	○	Highest importance

30. Unable to score/comments

____

____

____

____

____

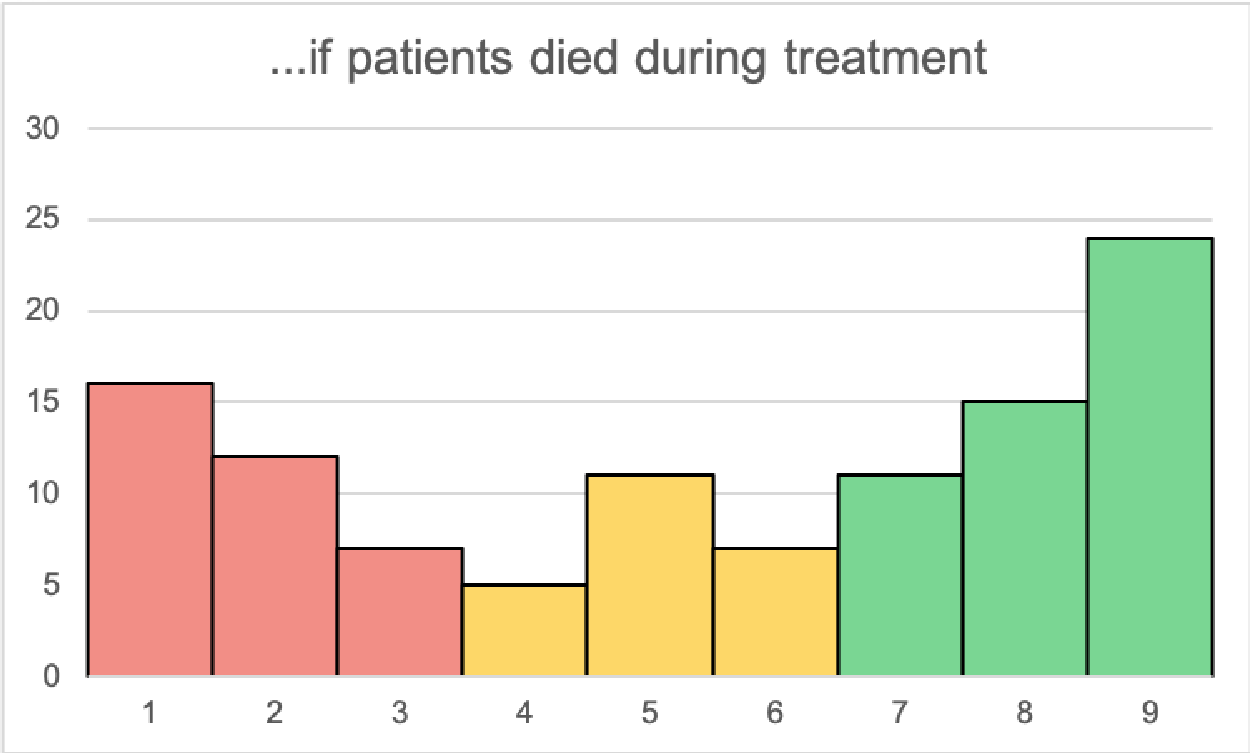

31. ...if patients died during treatment

Mark only one oval.

	1	2	3	4	5	6	7	8	9	
Lowest importance	⬭	⬭	⬭	⬭	⬭	⬭	⬭	⬭	⬭	Highest importance

32. Unable to score/comments

____

____

____

____

____

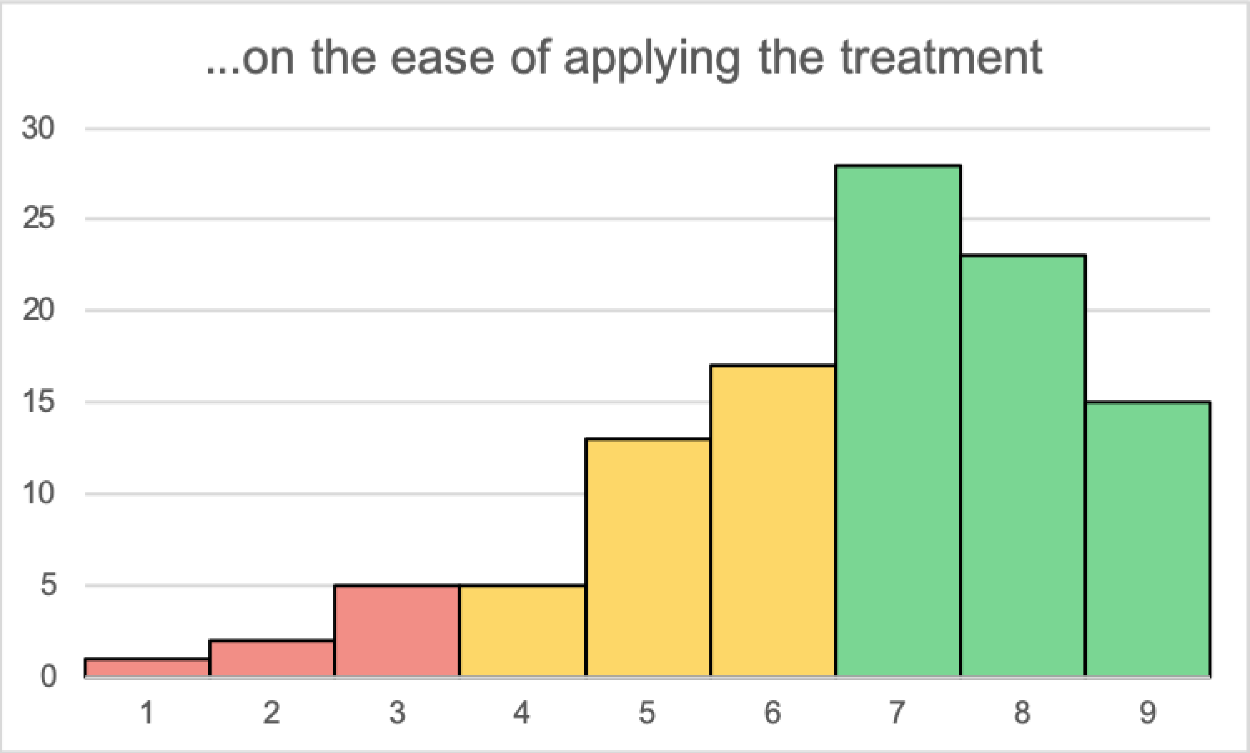

33. ...on the ease of applying the treatment

Mark only one oval.

	1	2	3	4	5	6	7	8	9	
Lowest importance	◯	◯	◯	◯	◯	◯	◯	◯	◯	Highest importance

34. Unable to score/comments

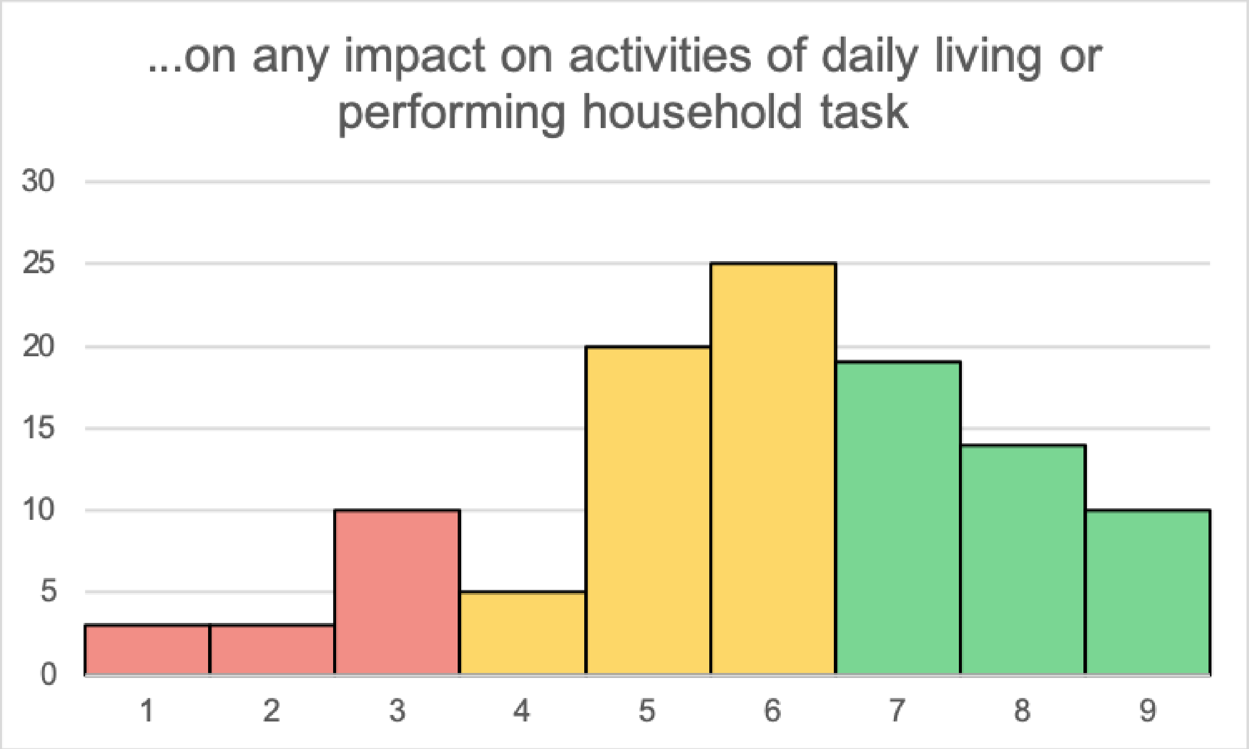

35. ...on any impact on activities of daily living or performing household task

Mark only one oval.

	1	2	3	4	5	6	7	8	9	
Lowest importance	◯	◯	◯	◯	◯	◯	◯	◯	◯	Highest importance

36. Unable to score/comments

____

____

____

____

____

[6-10] Studies should report...

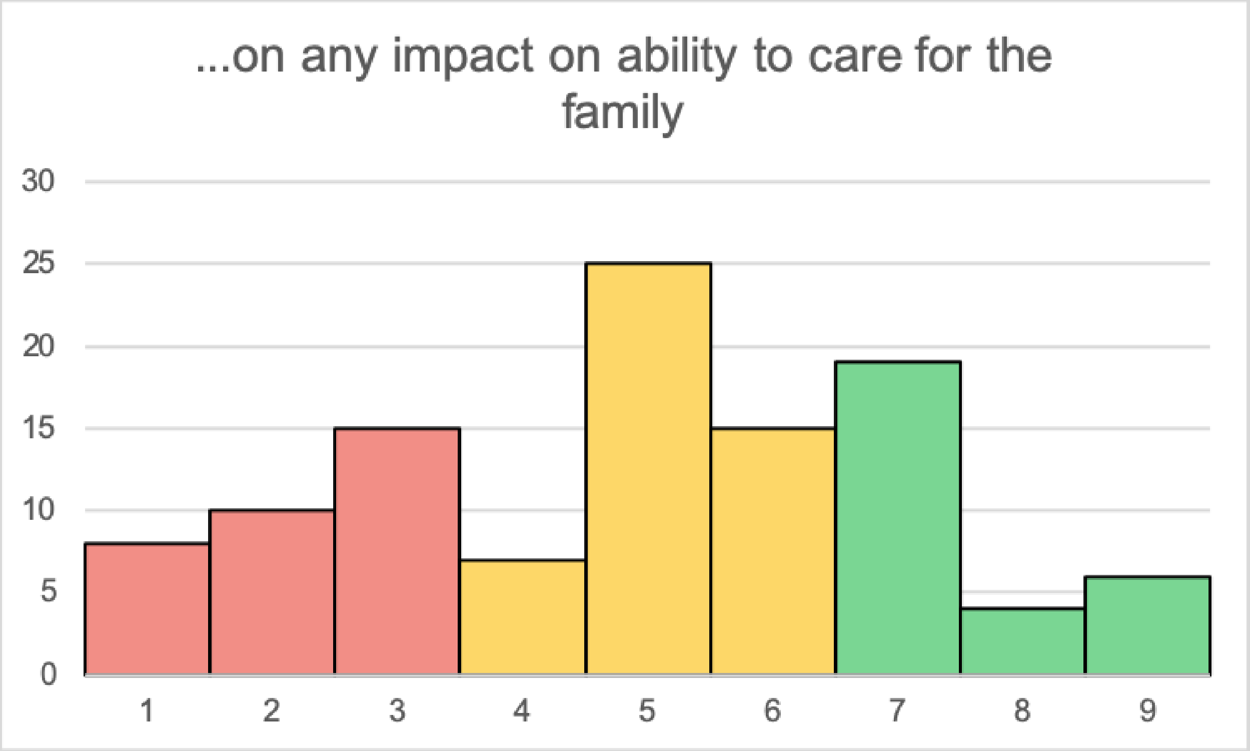

37. ...on any impact on ability to care for the family (for adults)

Mark only one oval.

	1	2	3	4	5	6	7	8	9	
Lowest importance	◯	◯	◯	◯	◯	◯	◯	◯	◯	Highest importance

38. Unable to score/comments

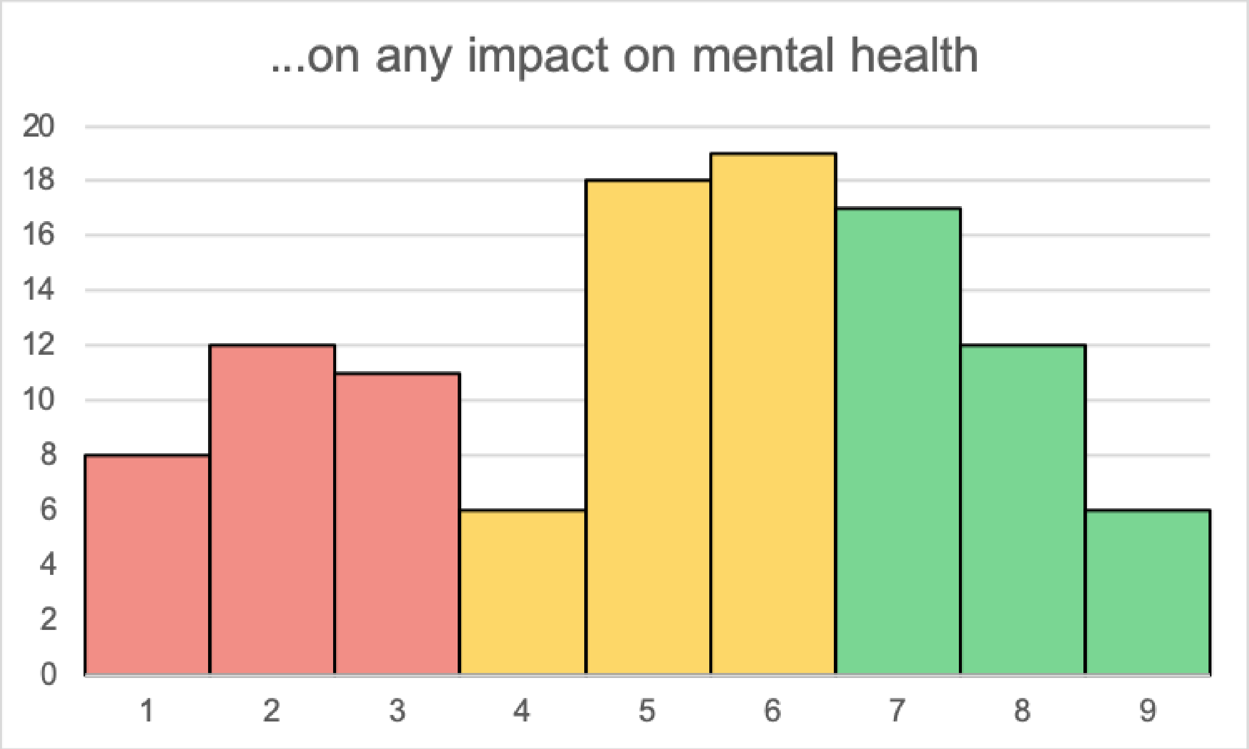

39. ...on any impact on mental health

Mark only one oval.

	1	2	3	4	5	6	7	8	9	
Lowest importance	◯	◯	◯	◯	◯	◯	◯	◯	◯	Highest importance

40. Unable to score/comments

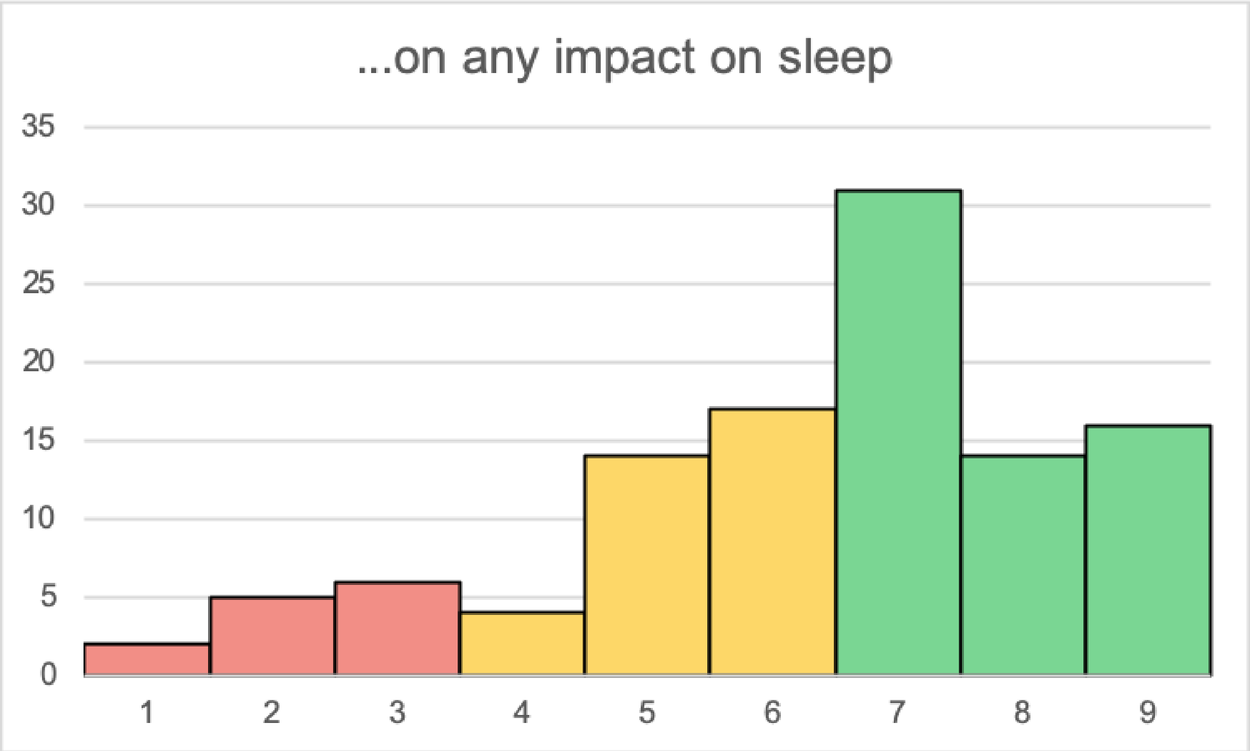

41. ...on any impact on sleep

Mark only one oval.

	1	2	3	4	5	6	7	8	9	
Lowest importance	◯	◯	◯	◯	◯	◯	◯	◯	◯	Highest importance

42. Unable to score/comments

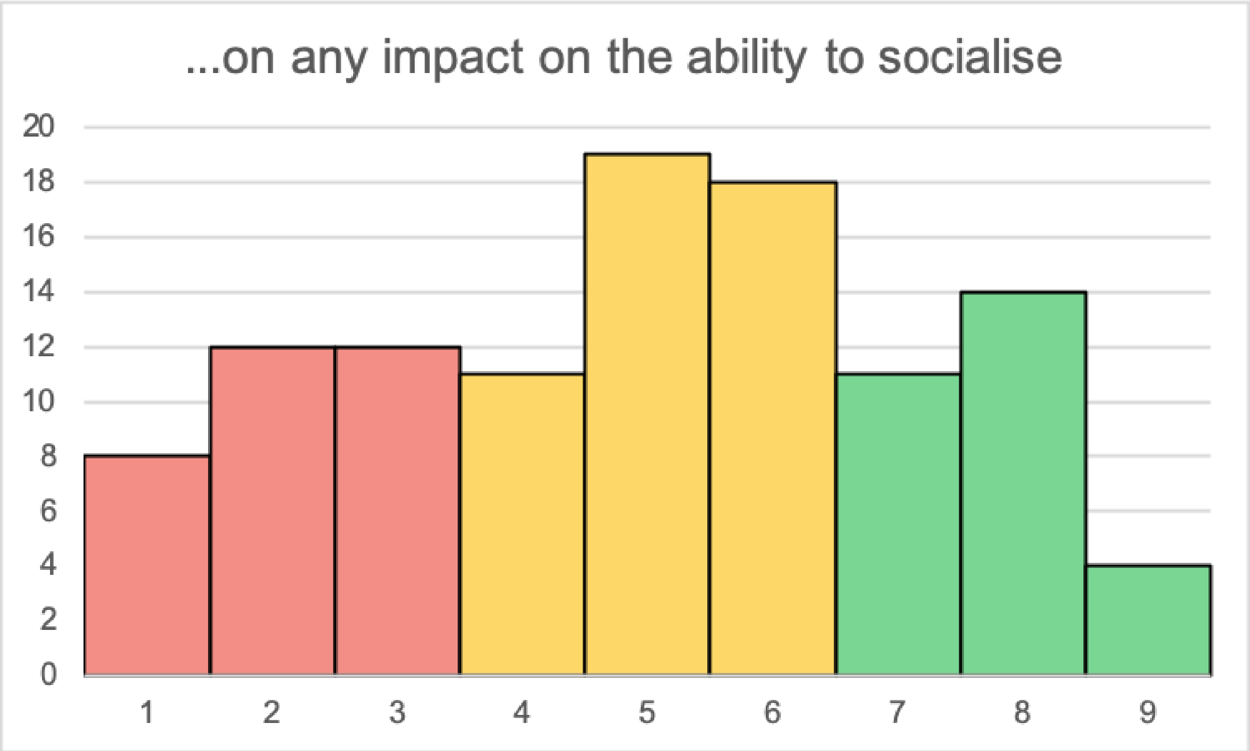

43. ...on any impact on the ability to socialise

Mark only one oval.

	1	2	3	4	5	6	7	8	9	
Lowest importance	◯	◯	◯	◯	◯	◯	◯	◯	◯	Highest importance

44. Unable to score/comments

____

____

____

____

____

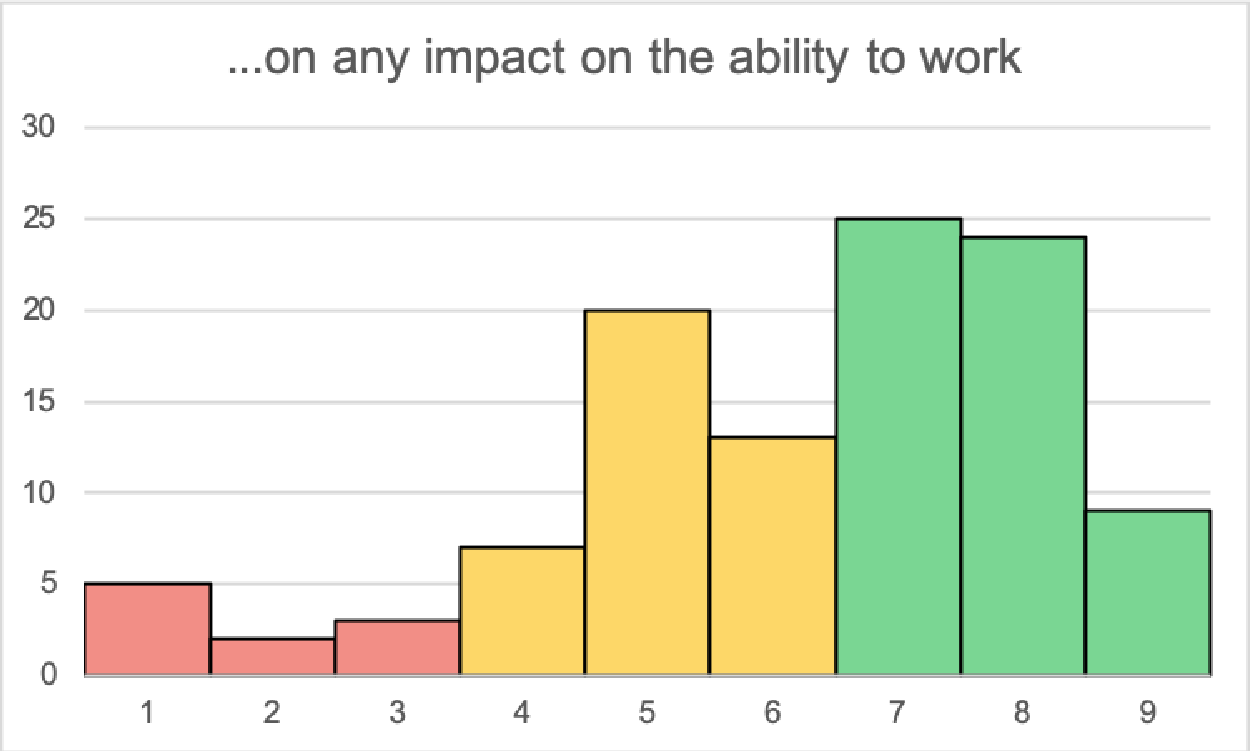

45. ...on any impact on the ability to work

Mark only one oval.

	1	2	3	4	5	6	7	8	9	
Lowest importance	◯	◯	◯	◯	◯	◯	◯	◯	◯	Highest importance

46. Unable to score/comments

[11-15] Studies should report...

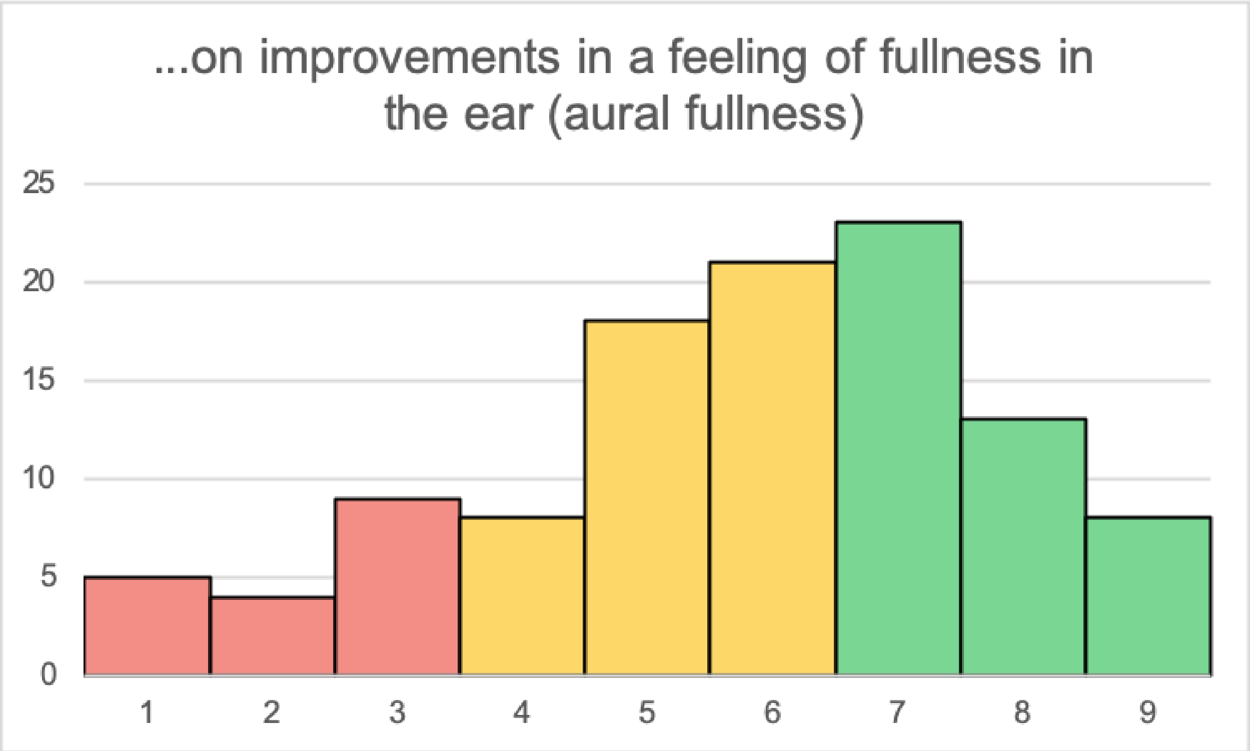

47. ...on improvements in a feeling of fullness in the ear (aural fullness)

Mark only one oval.

	1	2	3	4	5	6	7	8	9	
Lowest importance	◯	◯	◯	◯	◯	◯	◯	◯	◯	Highest importance

48. Unable to score/comments

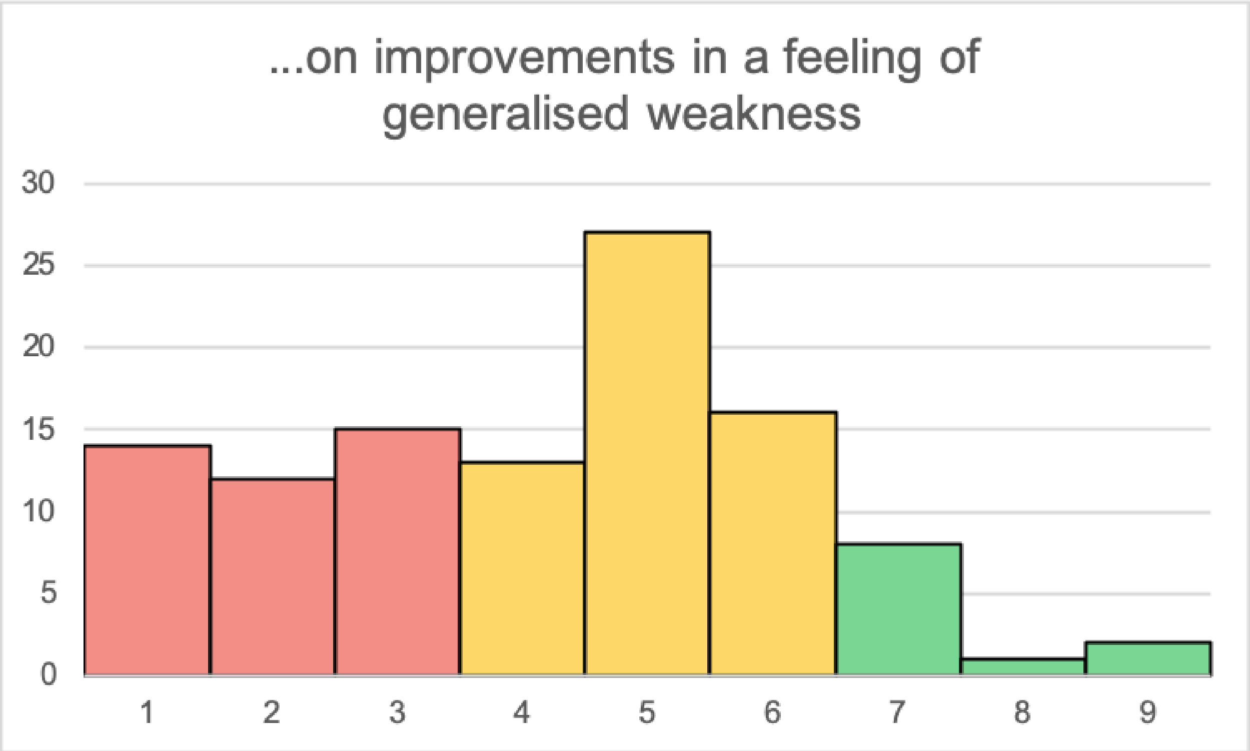

49. ...on improvements in a feeling of generalised weakness

Mark only one oval.

	1	2	3	4	5	6	7	8	9	
Lowest importance	◯	◯	◯	◯	◯	◯	◯	◯	◯	Highest importance

50. Unable to score/comments

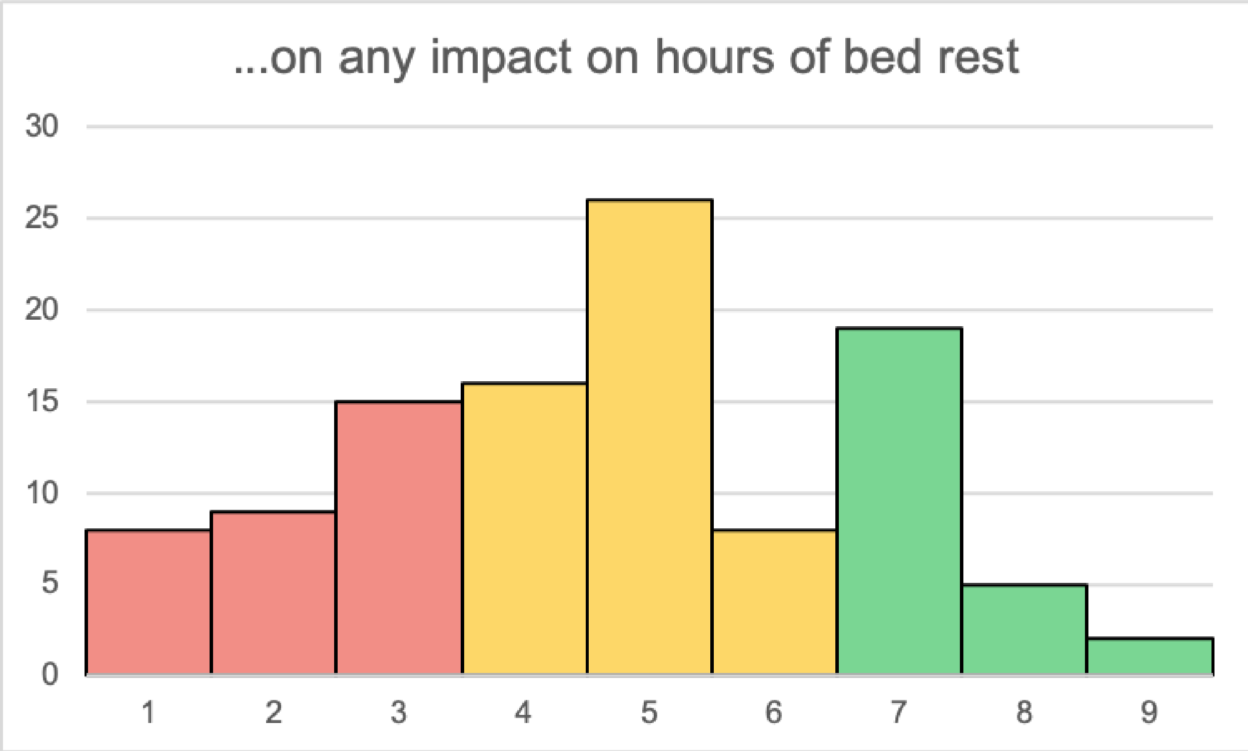

51. ...on any impact on hours of bed rest

Mark only one oval.

	1	2	3	4	5	6	7	8	9	
Lowest importance	◯	◯	◯	◯	◯	◯	◯	◯	◯	Highest importance

52. Unable to score/comments

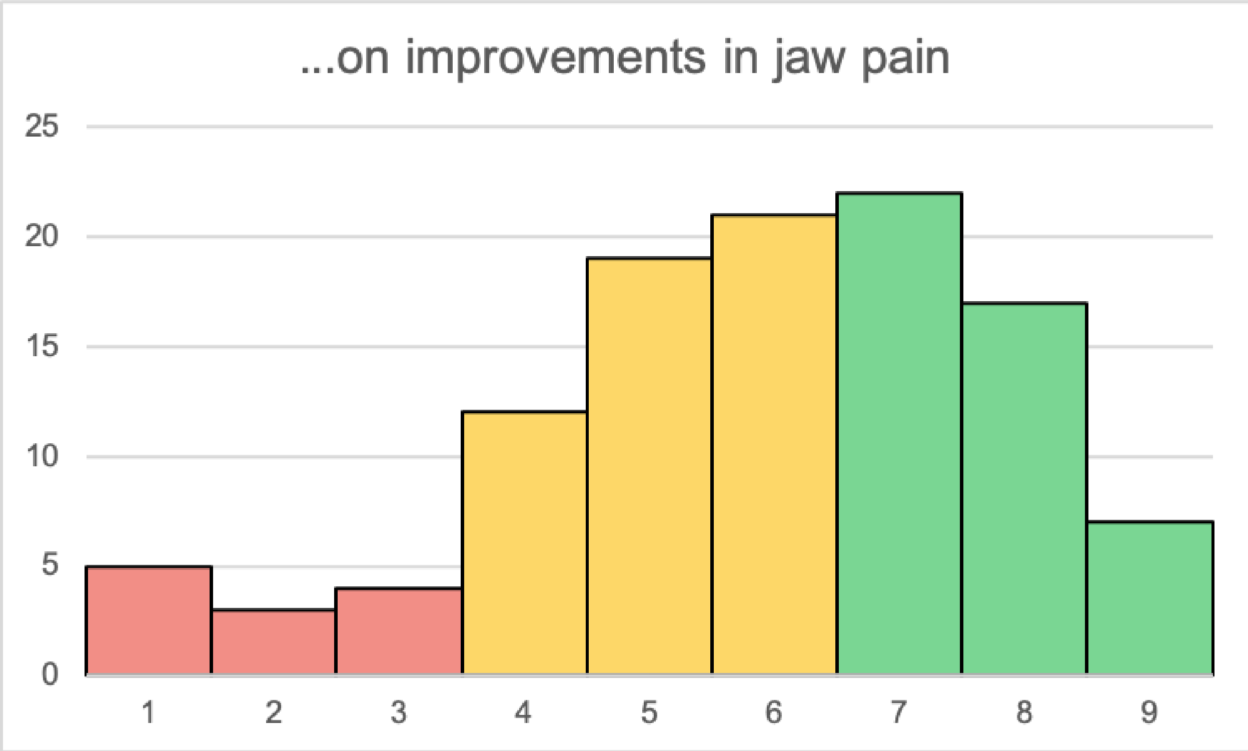

53. ...on improvements in pain in the jaw

Mark only one oval.

	1	2	3	4	5	6	7	8	9	
Lowest importance	◯	◯	◯	◯	◯	◯	◯	◯	◯	Highest importance

54. Unable to score/comments

____

____

____

____

____

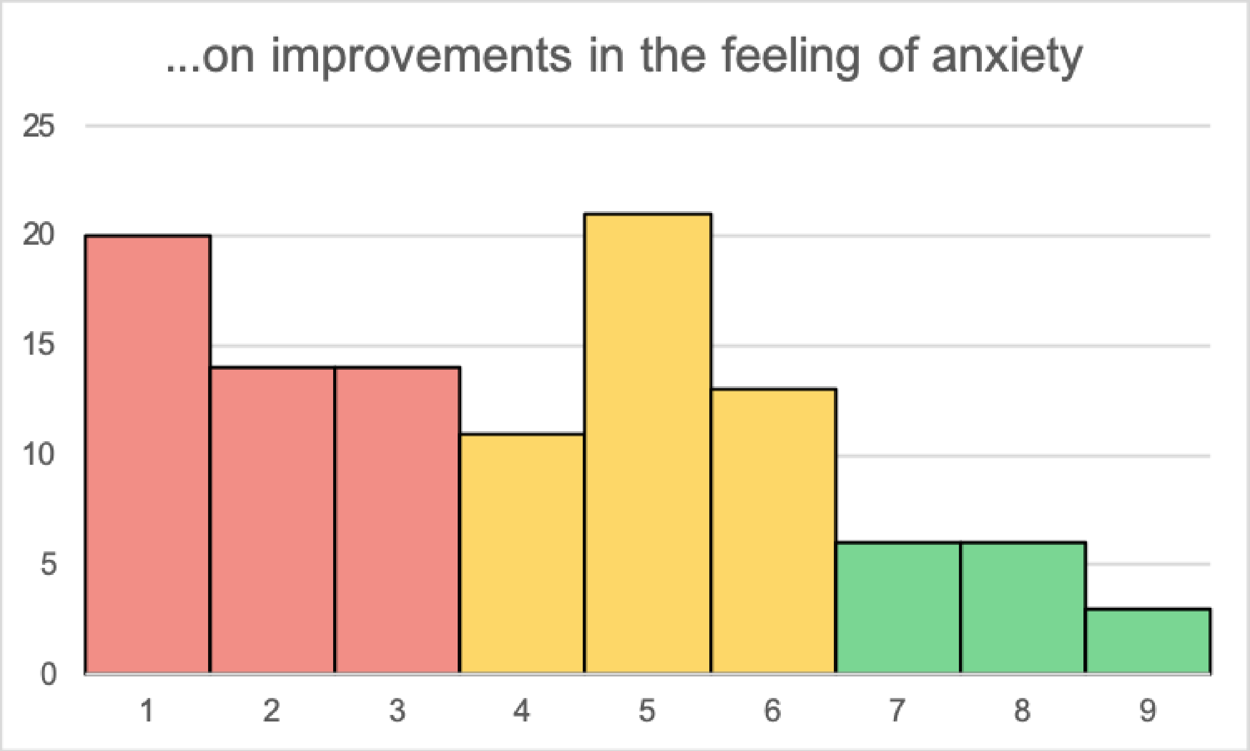

55. ...on improvements in the feeling of anxiety

Mark only one oval.

	1	2	3	4	5	6	7	8	9	
Lowest importance	◯	◯	◯	◯	◯	◯	◯	◯	◯	Highest importance

56. Unable to score/comments

[16-20] Studies should report...

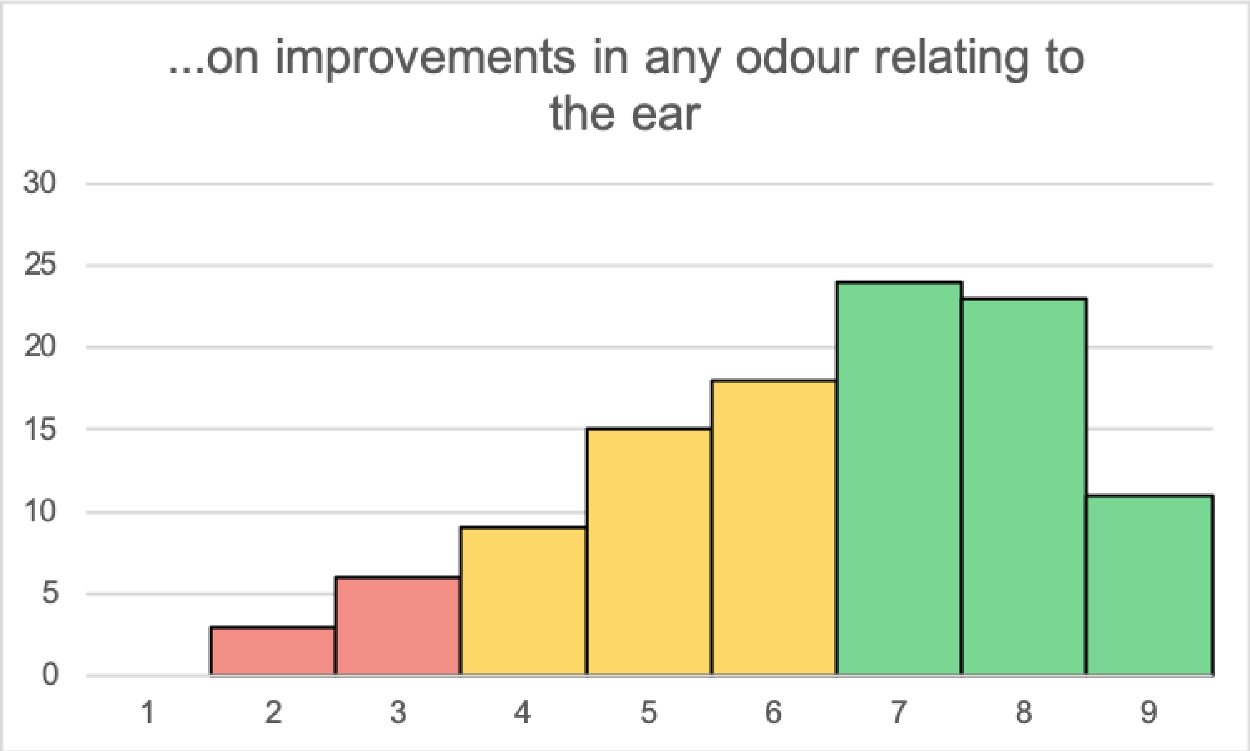

57. ...on improvements in any odour relating to the ear

Mark only one oval.

	1	2	3	4	5	6	7	8	9	
Lowest importance	◯	◯	◯	◯	◯	◯	◯	◯	◯	Highest importance

58. Unable to score/comments

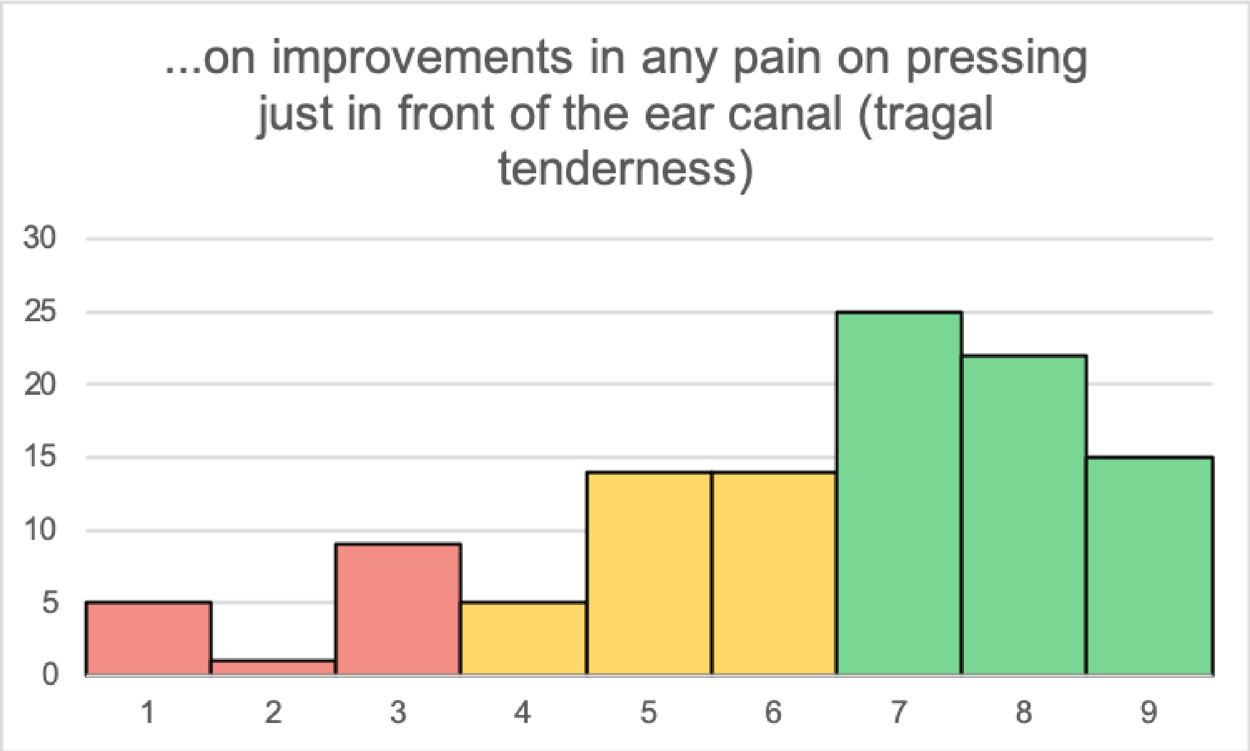

59. ...on improvements in any pain on pressing just in front of the ear canal (tragal tenderness)

Mark only one oval.

	1	2	3	4	5	6	7	8	9	
Lowest importance	◯	◯	◯	◯	◯	◯	◯	◯	◯	Highest importance

60. Unable to score/comments

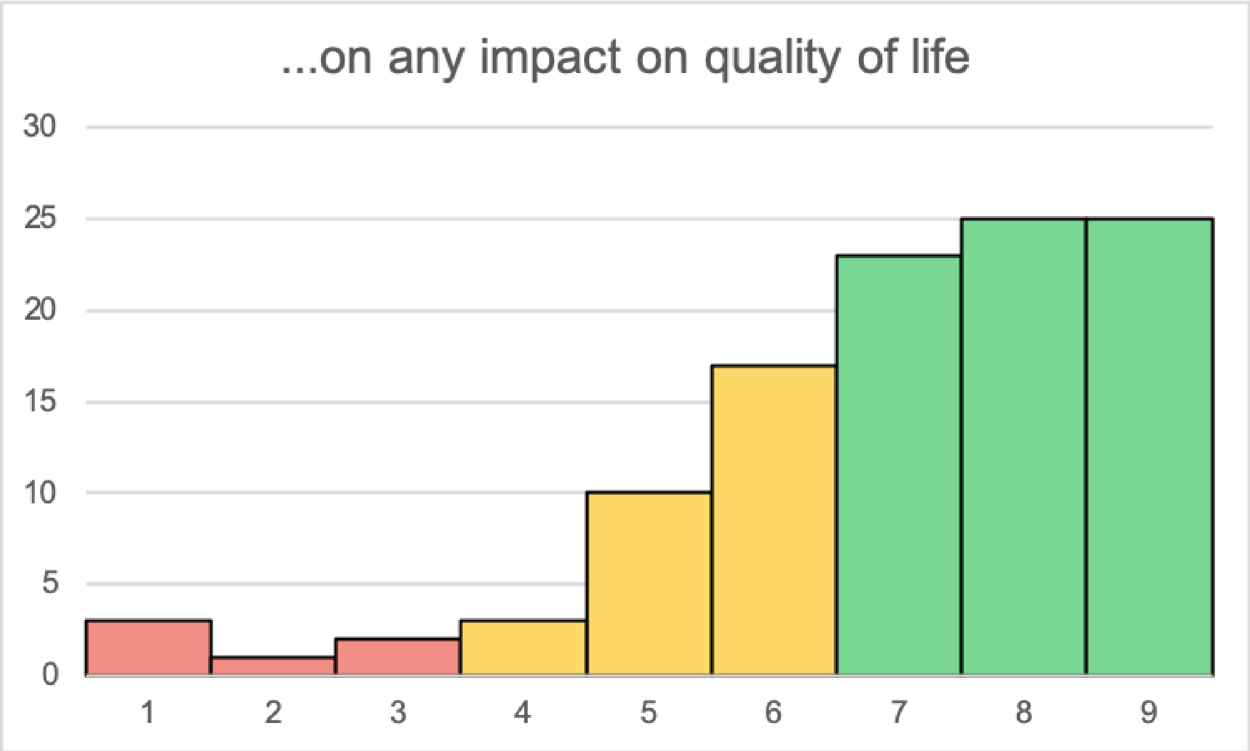

61. ...on any impact on quality of life

Mark only one oval.

	1	2	3	4	5	6	7	8	9	
Lowest importance	◯	◯	◯	◯	◯	◯	◯	◯	◯	Highest importance

62. Unable to score/comments

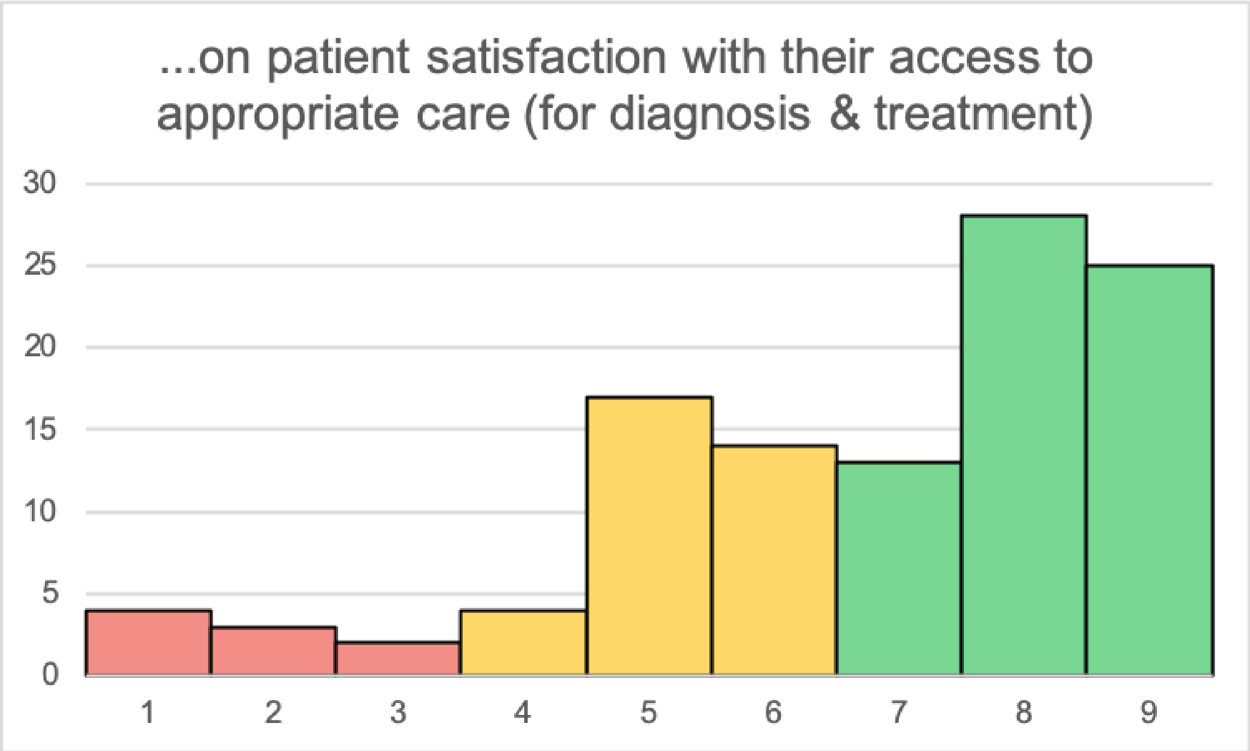

63. ...on patient satisfaction with their access to appropriate care (for diagnosis & treatment)

Mark only one oval.

	1	2	3	4	5	6	7	8	9	
Lowest importance	⬭	⬭	⬭	⬭	⬭	⬭	⬭	⬭	⬭	Highest importance

64. Unable to score/comments

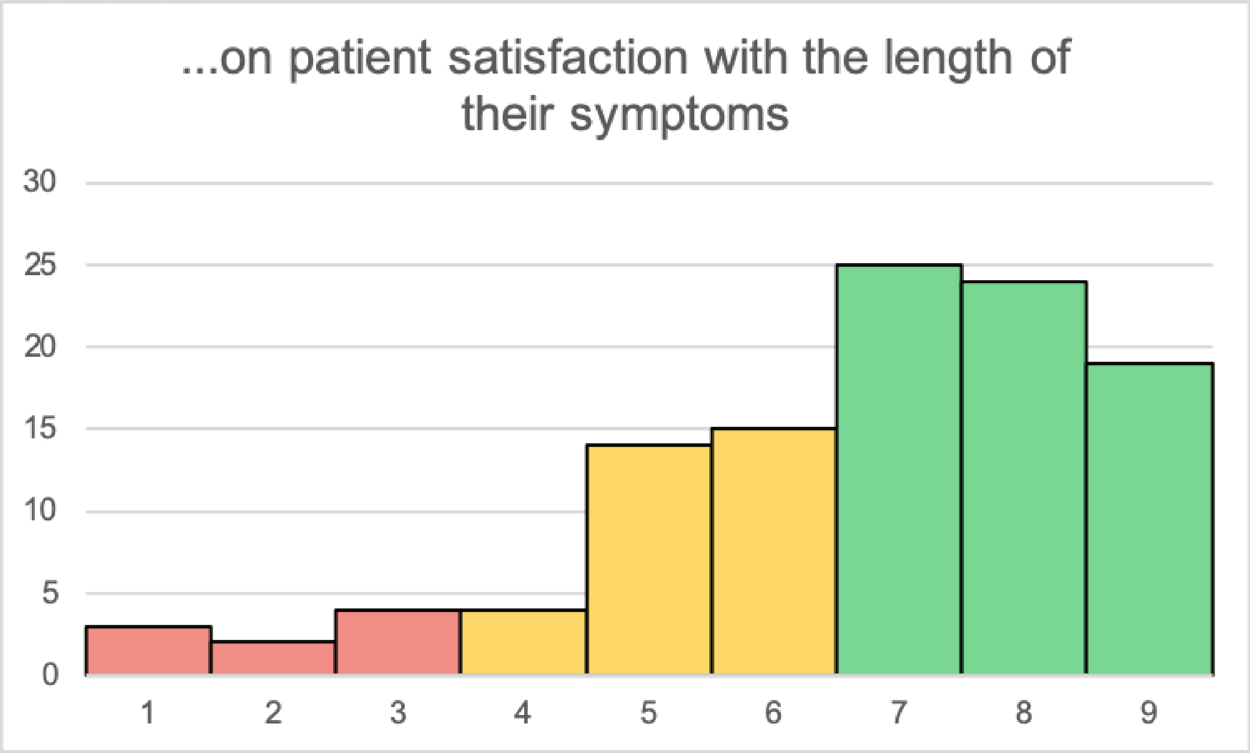

65. ...on patient satisfaction with the length of their symptoms

Mark only one oval.

	1	2	3	4	5	6	7	8	9	
Lowest importance	◯	◯	◯	◯	◯	◯	◯	◯	◯	Highest importance

66. Unable to score/comments

[21-25] Studies should report...

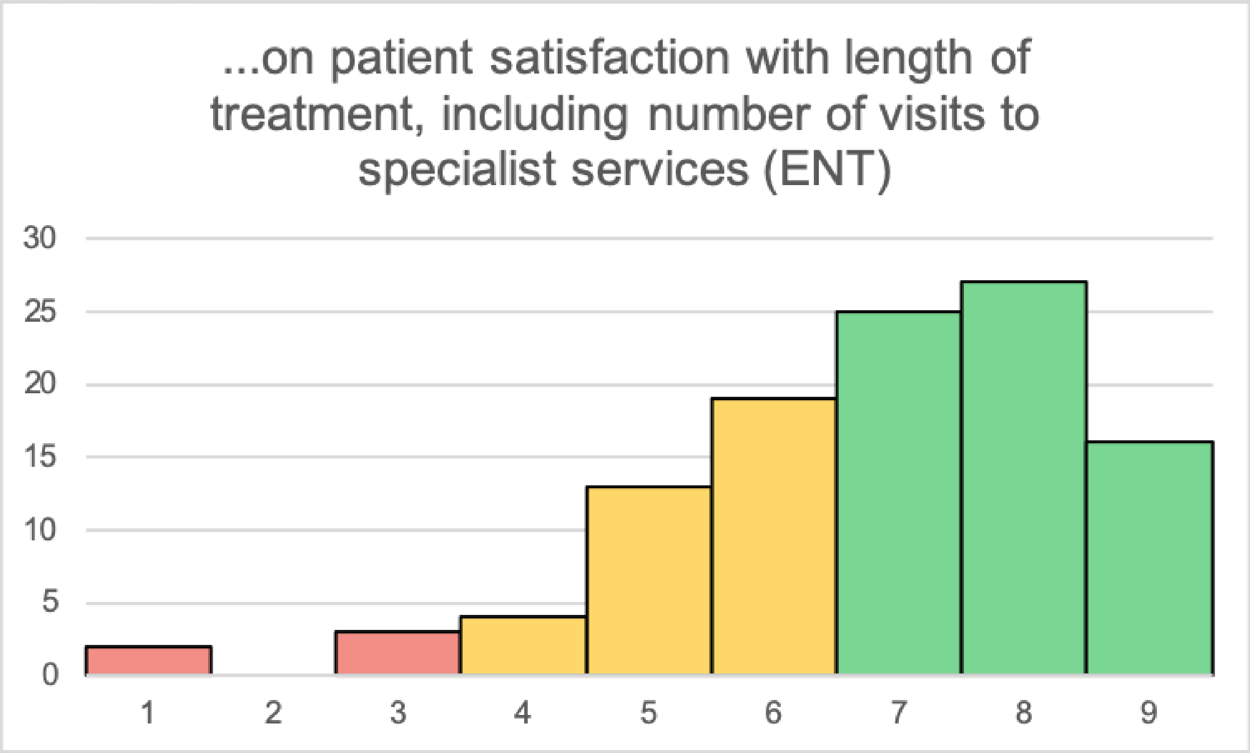

67. ...on patient satisfaction with length of treatment, including number of visits to specialist services (ENT)

Mark only one oval.

	1	2	3	4	5	6	7	8	9	
Lowest importance	⬭	⬭	⬭	⬭	⬭	⬭	⬭	⬭	⬭	Highest importance

68. Unable to score/comments

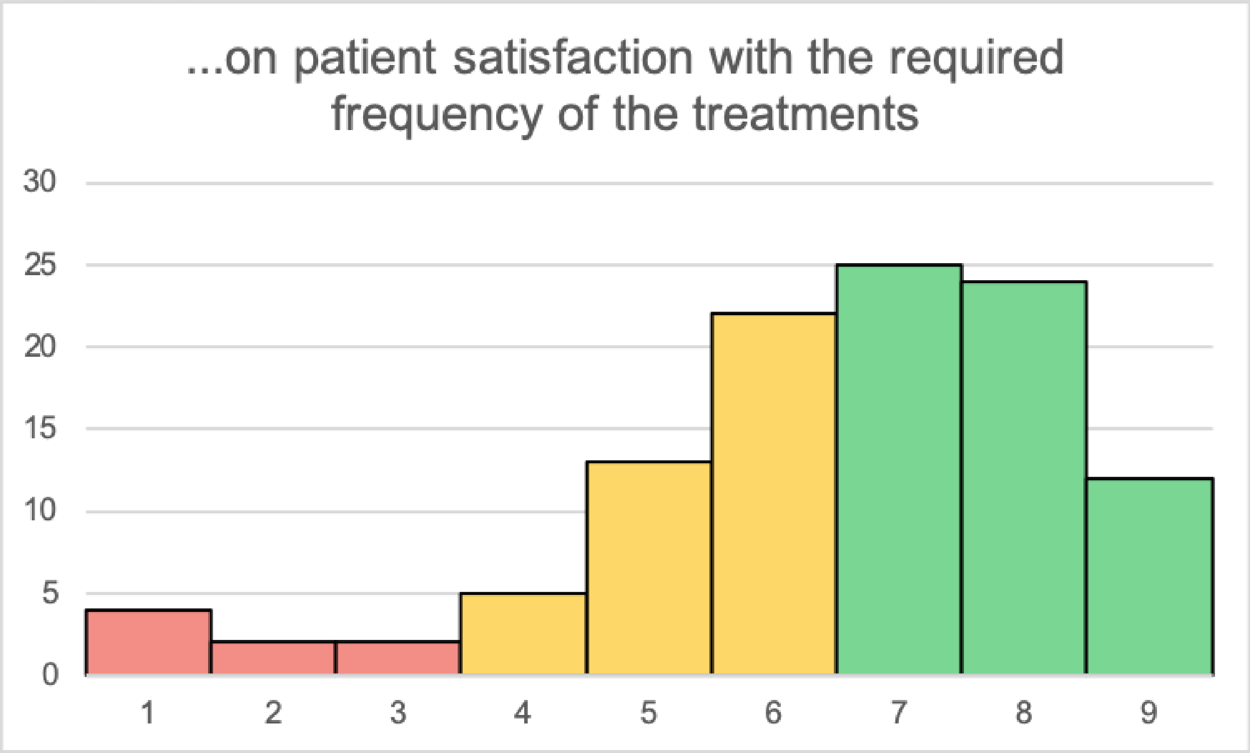

69. ...on patient satisfaction with the required frequency of the treatments

Mark only one oval.

	1	2	3	4	5	6	7	8	9	
Lowest importance	◯	◯	◯	◯	◯	◯	◯	◯	◯	Highest importance

70. Unable to score/comments

____
____
____
____
____

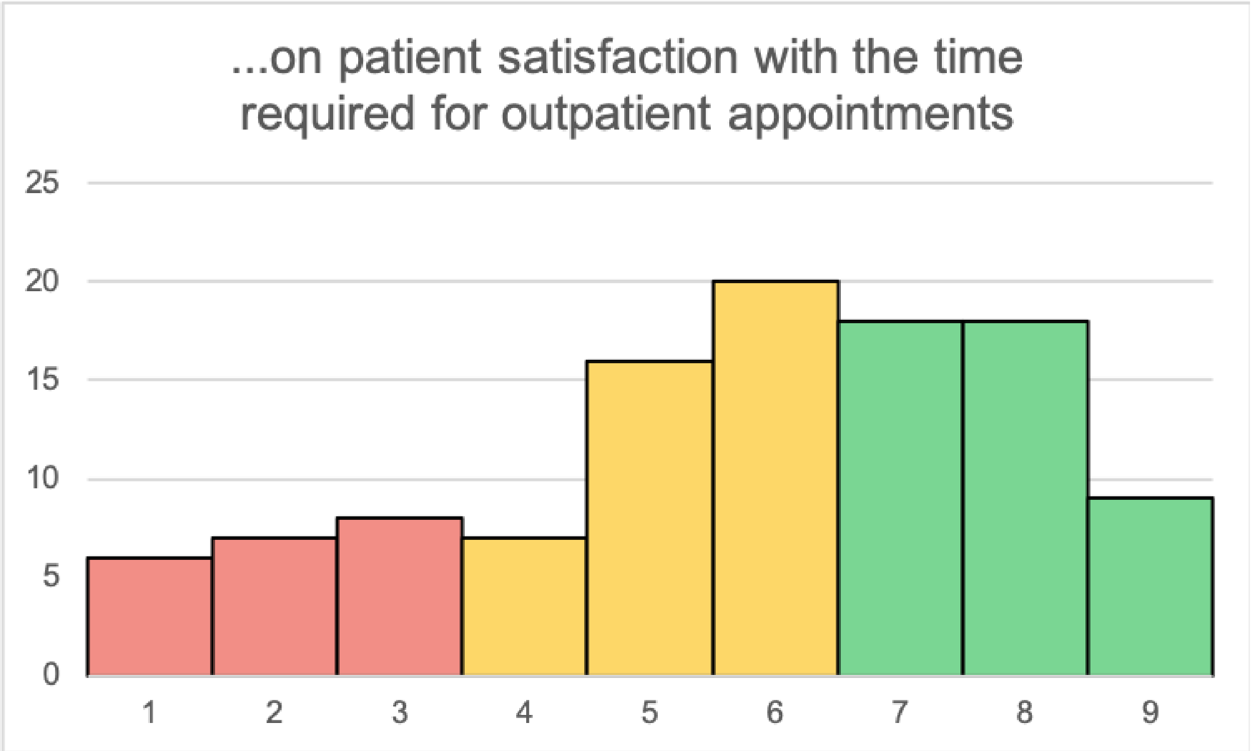

71. ...on patient satisfaction with the time required for outpatient appointments

Mark only one oval.

	1	2	3	4	5	6	7	8	9	
Lowest importance	⬭	⬭	⬭	⬭	⬭	⬭	⬭	⬭	⬭	Highest importance

72. Unable to score/comments

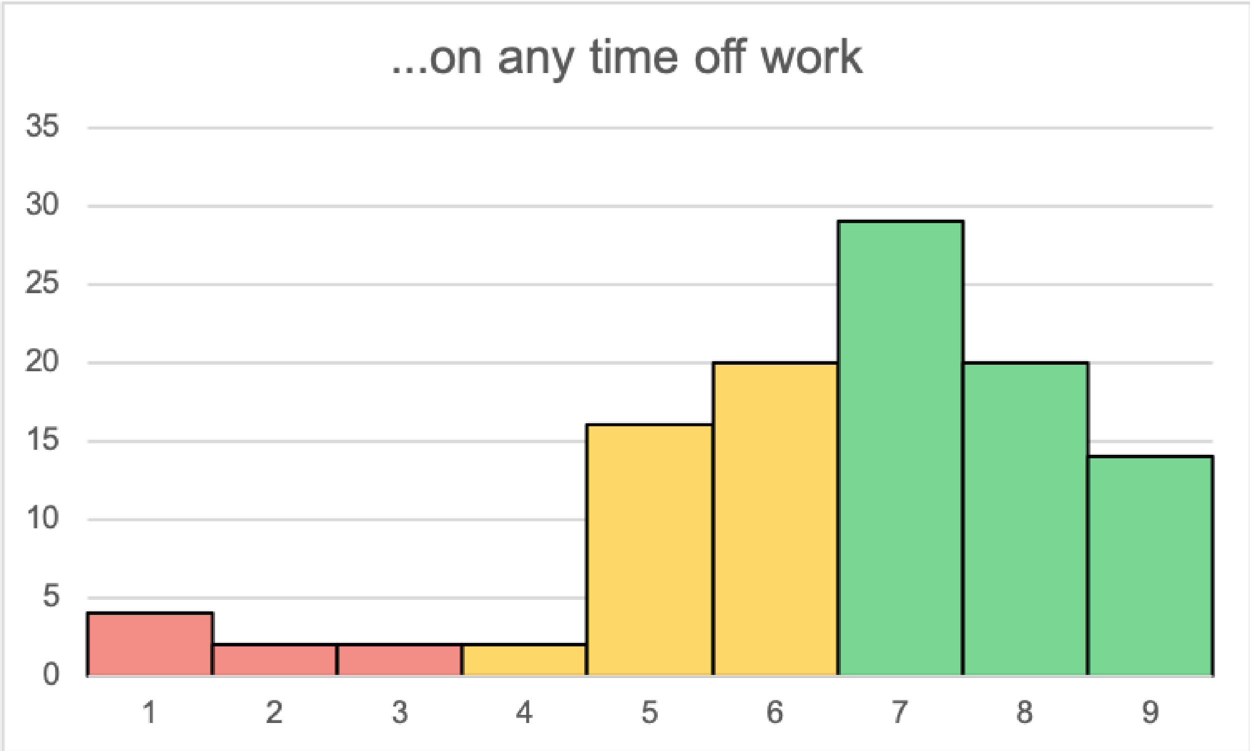

73. ...on any time off work

Mark only one oval.

	1	2	3	4	5	6	7	8	9	
Lowest importance	◯	◯	◯	◯	◯	◯	◯	◯	◯	Highest importance

74. Unable to score/comments

____

____

____

____

____

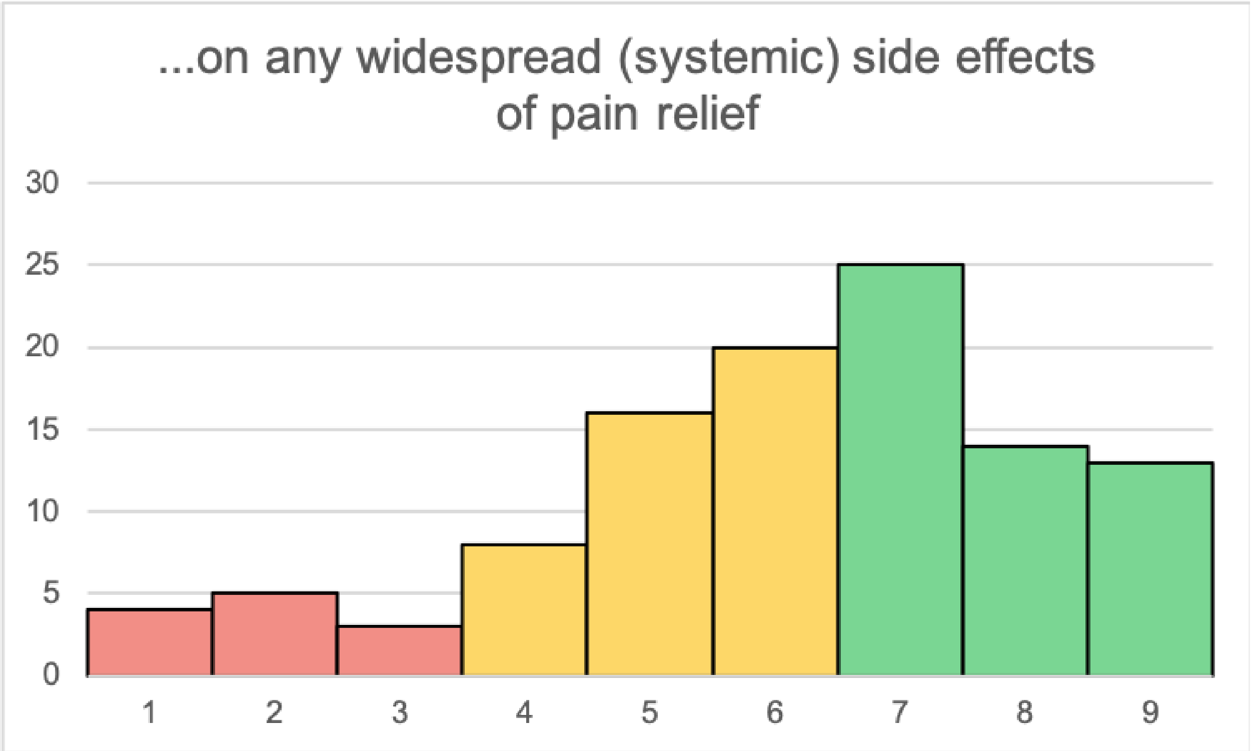

75. ...on any widespread (systemic) side effects of pain relief

Mark only one oval.

	1	2	3	4	5	6	7	8	9	
Lowest importance	◯	◯	◯	◯	◯	◯	◯	◯	◯	Highest importance

76. Unable to score/comments

NEW ITEMS:

[1-4] Studies should report...

77. ...the time to resolution of symptoms

Mark only one oval.

	1	2	3	4	5	6	7	8	9	
Lowest importance	⬭	⬭	⬭	⬭	⬭	⬭	⬭	⬭	⬭	Highest importance

78. Unable to score/comments

____

____

____

____

____

*** THANK YOU ***

That's it!

Thank you for taking part. Your responses will help create the diagnostic criteria and a core outcome set for acute otitis externa. This is round 2 of the process. We'll give you the feedback from everyone's responses to this round, then ask if this influences your answers for the next round, to reach a consensus.

Once you're happy with your responses, please hit submit to record them.

Thank you once again for taking part. If you have any problems at any stage, please email info@entintegrate.org

INTEGRATE DECODE steering committee

This content is neither created nor endorsed by Google.

Google Forms

The DECODE Project: Delphi round 3

*Required

1. Email address *

Your email address is used to track your participation and allow you to review your responses. By entering your email you agree to the DECODE team using it only for the purposes of conducting this survey and communicating the results to you. You can withdraw your consent at anytime.

DEveloping consensus Core Outcomes and Diagnostic criteria for acute otitis Externa

Welcome to round 3

Thank you for participating as a stakeholder in the second round of a Delphi process to develop a diagnostic criteria and core outcome set (COS) for research into acute otitis externa.

Your answers to these survey questions are anonymous to everyone outside of the project steering committee, so please answer freely.

Participation in all three stages of the Delphi will mean you are recognised as a collaborator (identifiable on PubMed) in any subsequent presentations and publications resulting from this work.

You may have been provided with a Participant Information Sheet. A copy of this, should you wish to read it, may be found here: https://ENTintegrate.org/decode. Here you will find additional information in Frequently Asked Questions about how this Delphi process was developed, and also information on what will happen once you have completed this questionnaire.

Rationale

Research into otitis externa is heterogenous, with researchers using a wide variety of different diagnostic criteria and outcomes. This makes direct comparisons between previous studies difficult.

Our project aims to develop diagnostic criteria and a core outcome set for otitis externa to help standardise future research. We aim to survey key patient and professional stakeholders in an attempt to reach consensus.

Survey methods

For the diagnosis, you will be asked which features you think are important to be included in a minimum diagnostic criteria.

For the Core Outcome Set, the Delphi process involves answering questions on three separate occasions to gather your opinions relating to the importance of different outcome measures for otitis externa:

Completed:
- ROUND 1: You were asked to rate all the possible outcomes and suggest any additional outcomes you think may be important.
- ROUND 2: You were shown summaries of how YOU AND YOUR PEERS responded to items that did not reach consensus in round 1. You were asked to rate these items again, in light of this information.

Now:
- ROUND 3: You will be shown summaries of how PATIENTS AND PROFESSIONALS responded to items that did not yet reach consensus. You are asked to rate these items again in light of this information.

Final consensus

After these 3 rounds, the outcomes reaching consensus (using criteria explained in the protocol) will either be included or excluded from the final Core Outcome Set. Outcomes not reaching a consensus will be reviewed by the DECODE steering committee and a final consensus will be reached as to whether the item should be included or not, based on the information from the Delphi process from each stakeholder group. The resulting Core Outcome Set will be submitted for publication in a peer reviewed journal.

Thank you

Thank you once again for taking part. If you have any problems at any stage, please email info@entintegrate.org

INTEGRATE DECODE steering committee

2. Please select you stakeholder group *

Mark only one oval.

- Audiologist
- ENT registrar
- ENT consultant - Otologist
- ENT consultant - General / Non-otology
- GP
- Junior doctor (below ST3)
- Microbiologist
- Nurse

Changes to the survey

Thank you again for all the comments you have provided. These have all been reviewed by the steering committee. As a result of this feedback, the phrasing of some of the questions has been amended. All your comments will be used to inform the final consensus process in instances where there remains disagreement after the 3 consultation rounds.

SECTION 1: Diagnosis of acute otitis externa

There are currently no accepted diagnostic criteria for acute otitis externa (AOE), which complicates patient selection for trials.

For the purposes of these diagnostic criteria:
- Super localised infections of the external ear (e.g. furuncles) are excluded.
- Uncomplicated otitis externa is considered infection without spread beyond the soft tissues of the external ear.
- Necrotising otitis externa/skull base osteomyelitis is considered a different entity

Results from round 2:

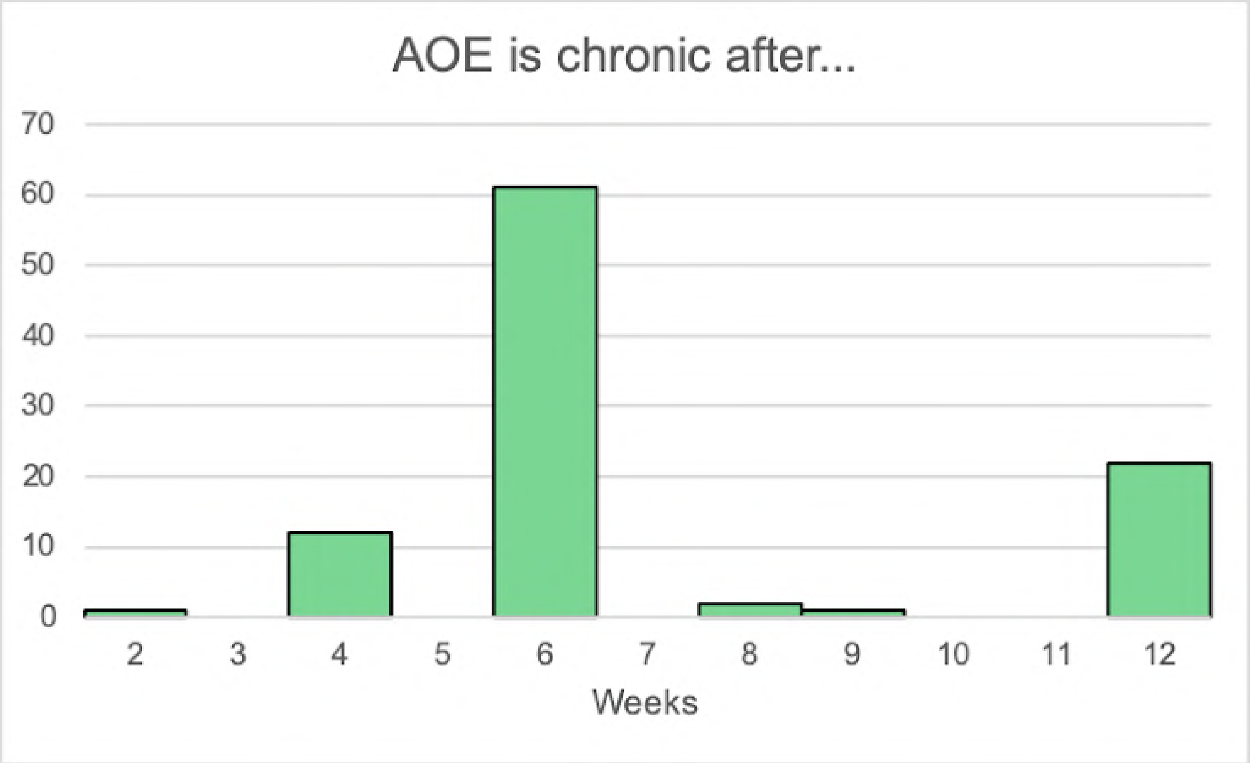

3. From the onset of symptoms, treated or untreated acute otitis externa should be considered to have become 'chronic' after... *

Mark only one oval.

- 2 weeks
- 3 weeks
- 4 weeks
- 5 weeks
- 6 weeks
- 7 weeks
- 8 weeks
- 9 weeks
- 10 weeks
- 11 weeks
- 12 weeks

4. Comments

SIGNS & SYMPTOMS

We'd like to establish the KEY FEATURES in the diagnosis of acute otitis externa.

Consensus results after round 2

7 of 14 items have now reached consensus for the DIAGNOSTIC CRITERIA for AOE:

6 items have been included:
- External auditory canal erythema
- Itchiness
- Tragal tenderness
- External auditory canal oedema
- Otalgia (ear pain)
- Otorrhoea (discharge from the ear)

1 item has been excluded:
- Generalised lethargy

7 items are yet to reach consensus:
- Aural Fullness
- External auditory canal granulations
- Hearing impairment
- Jaw pain
- Microbiological identification of an organism
- Odour related to the ear
- Squamous debris/keratin

Items reaching consensus in round 1:

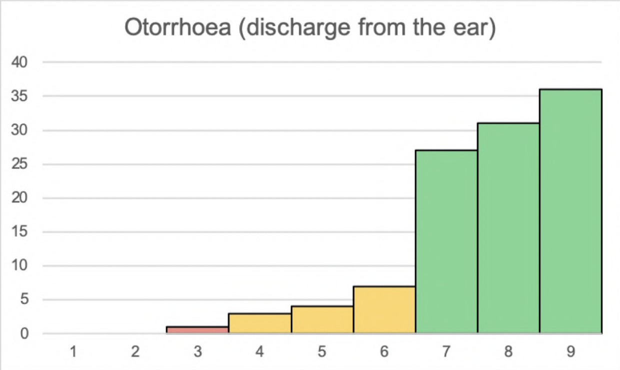

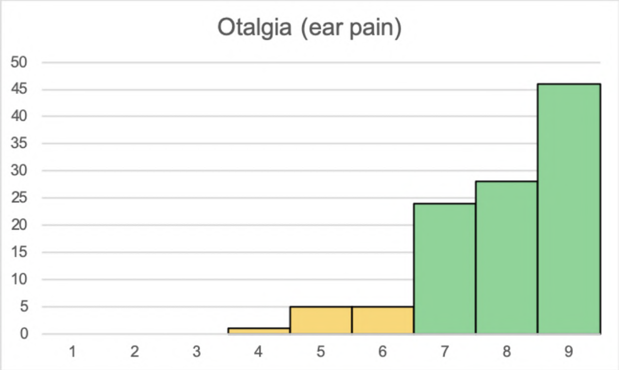

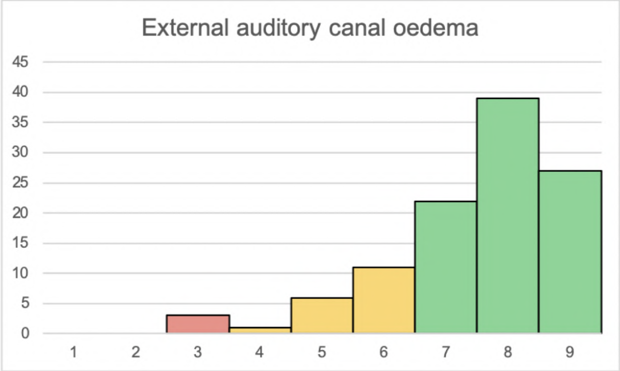

Items reaching consensus in round 2:

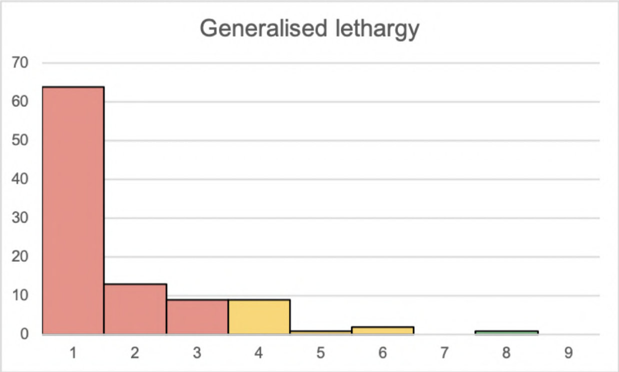

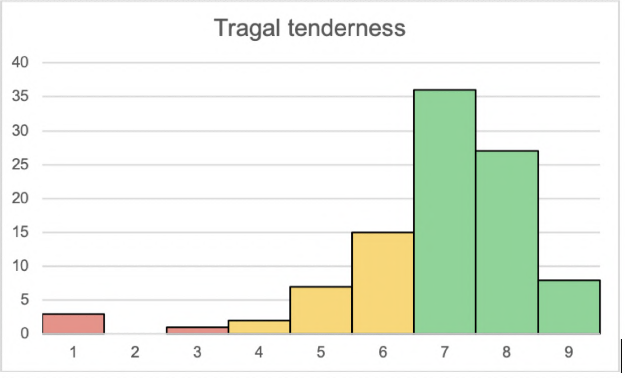

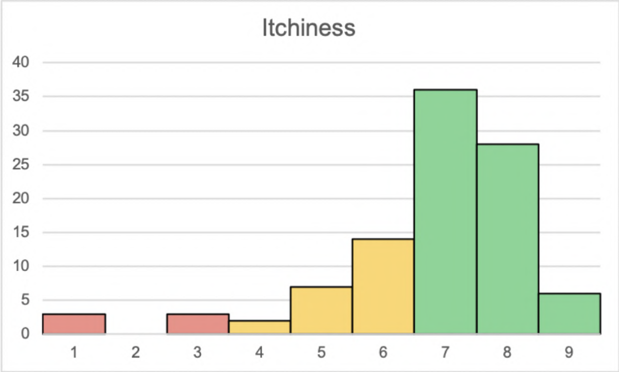

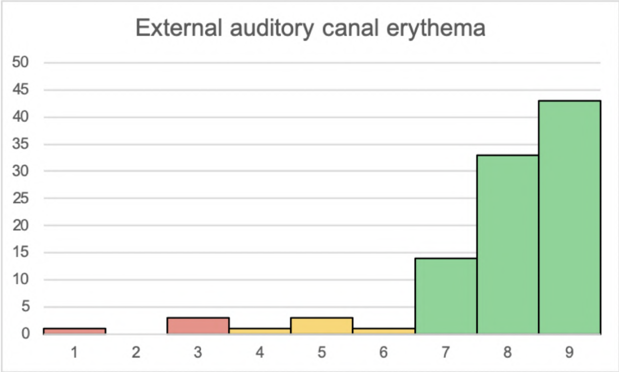

Please rate the importance of the following signs and symptoms of AOE, INCLUSION or EXCLUSION from a DIAGNOSTIC CRITERIA: [7 items remaining]

Results from round 2:

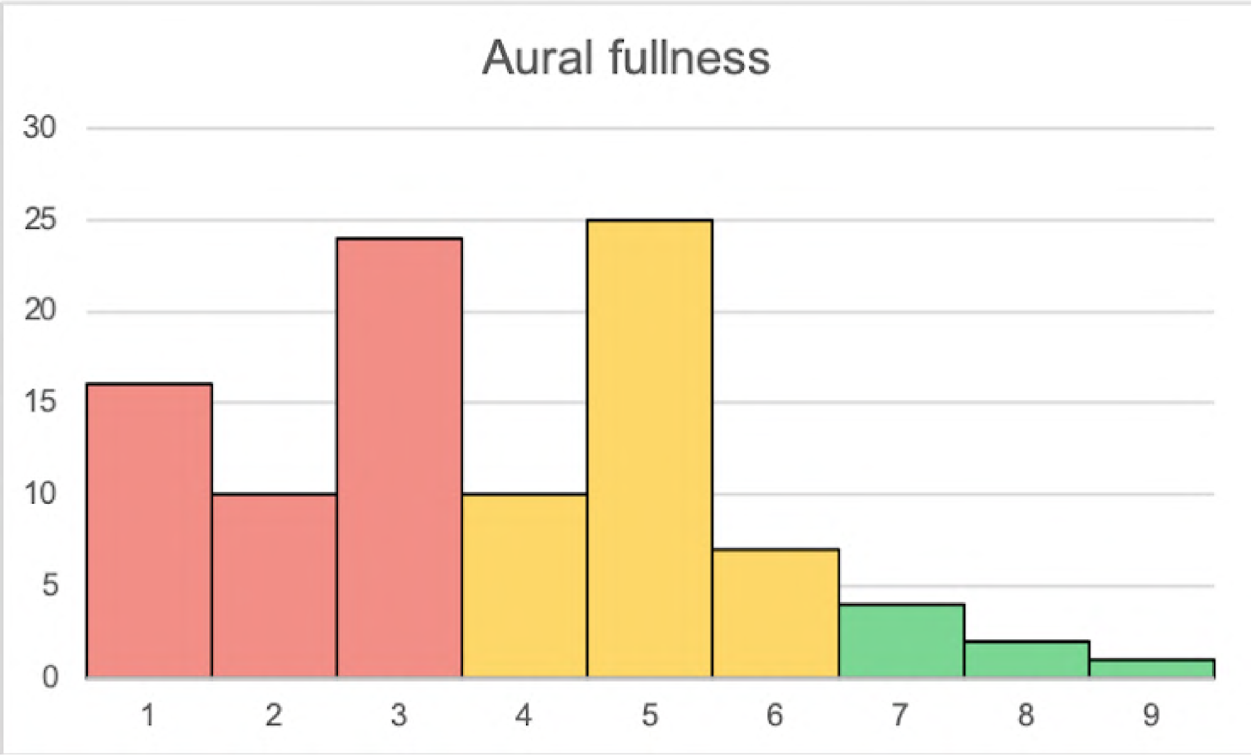

5. Your answer for round 3: Aural fullness

Mark only one oval.

	1	2	3	4	5	6	7	8	9	
Lowest importance	◯	◯	◯	◯	◯	◯	◯	◯	◯	Highest importance

6. Unable to score/comments

Results from round 2:

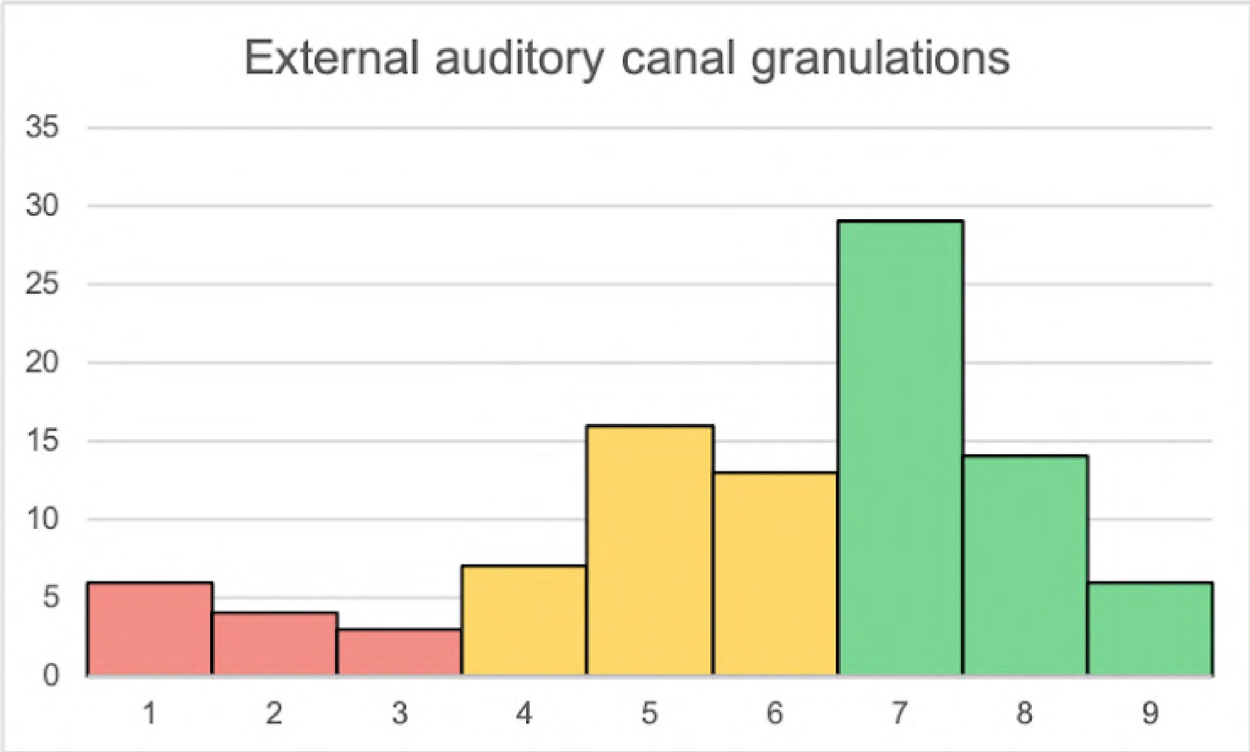

7. Your answer for round 3: External auditory canal granulations

Mark only one oval.

	1	2	3	4	5	6	7	8	9	
Lowest importance	⬭	⬭	⬭	⬭	⬭	⬭	⬭	⬭	⬭	Highest importance

8. Unable to score/comments

____

____

____

____

____

Results from round 2:

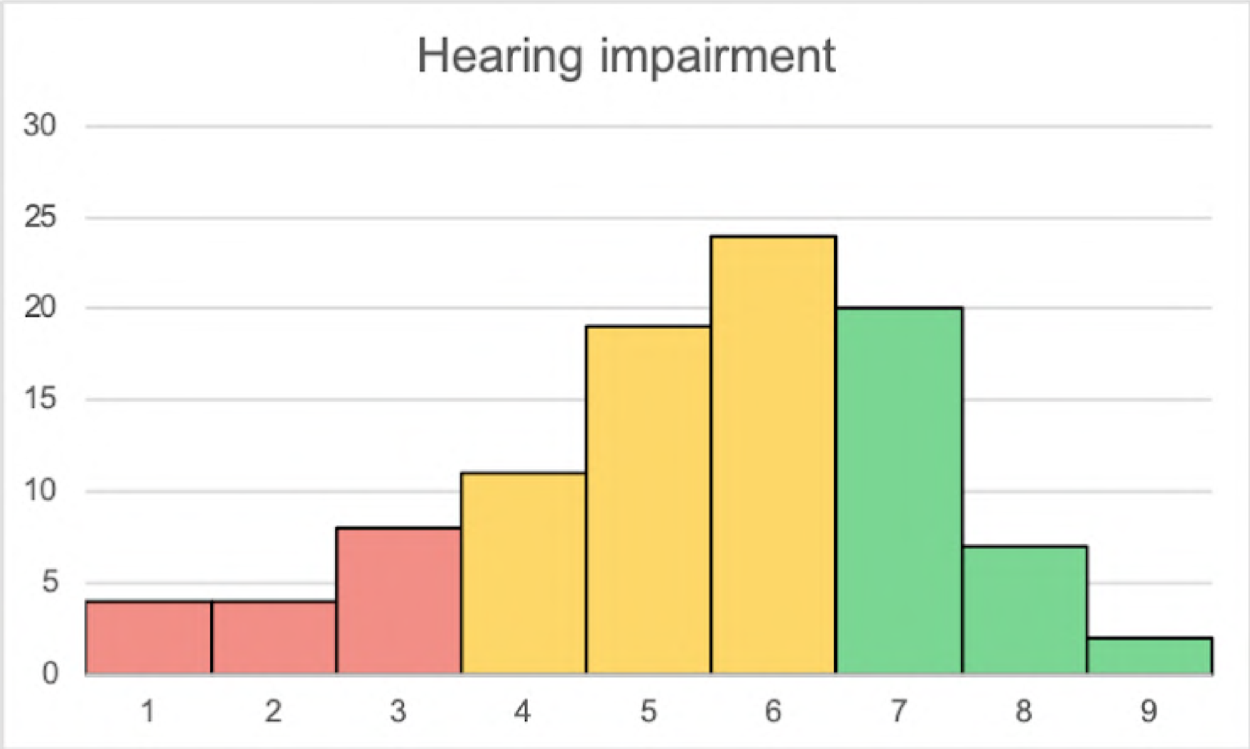

9. Your answer for round 3: Hearing impairment

Mark only one oval.

	1	2	3	4	5	6	7	8	9	
Lowest importance	◯	◯	◯	◯	◯	◯	◯	◯	◯	Highest importance

10. Unable to score/comments

Results from round 2:

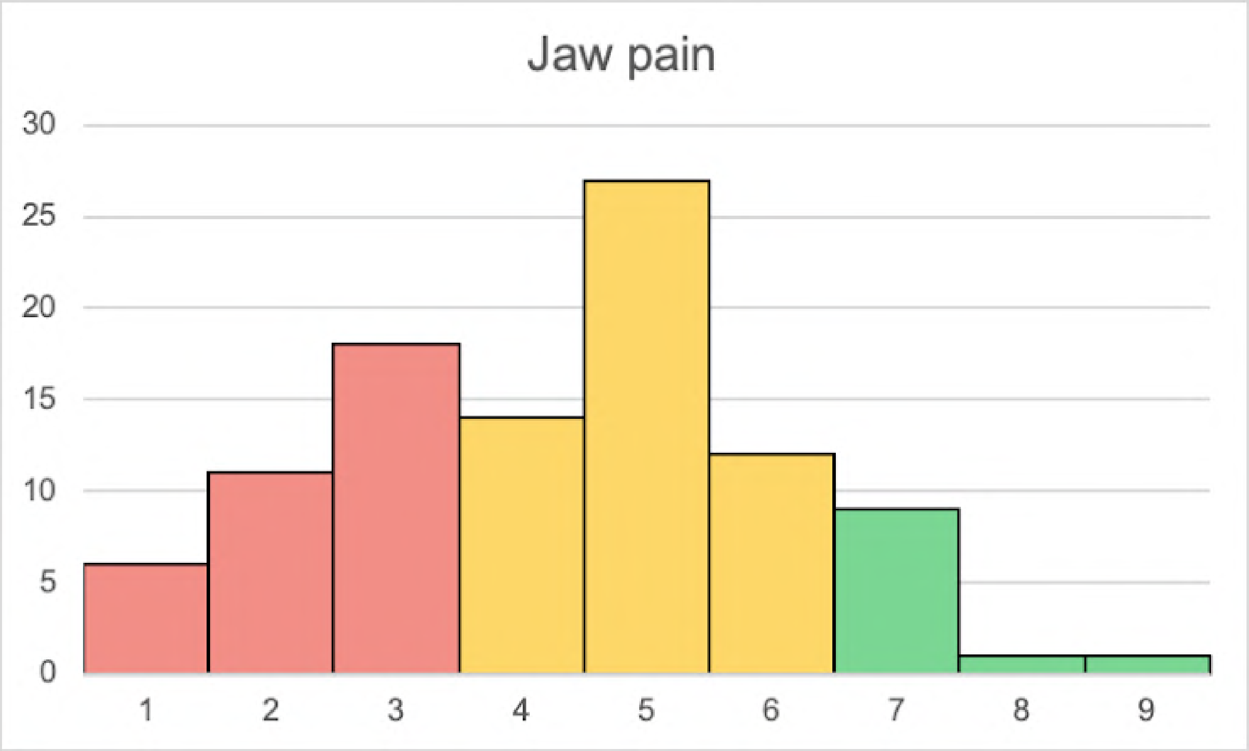

11. Your answer for round 3: Jaw pain

Mark only one oval.

	1	2	3	4	5	6	7	8	9	
Lowest importance	◯	◯	◯	◯	◯	◯	◯	◯	◯	Highest importance

12. Unable to score/comments

Results from round 2:

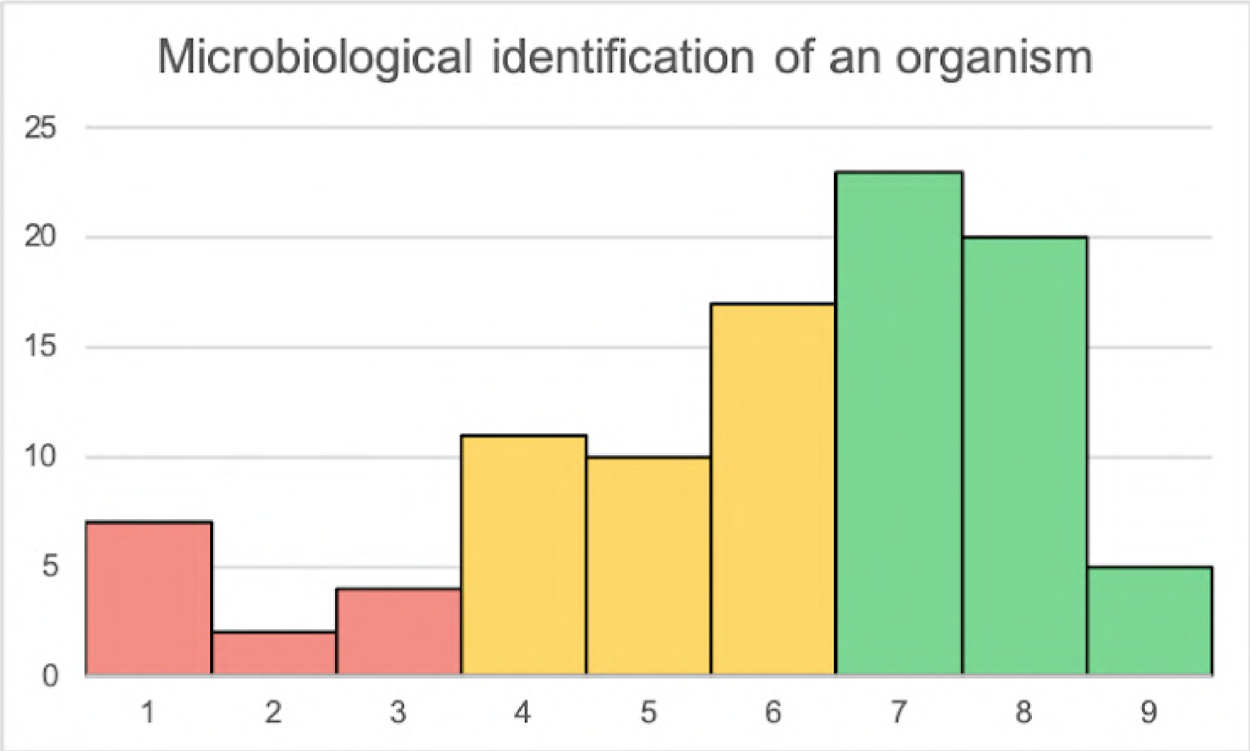

13. Your answer for round 3: Microbiological identification of an organism

Mark only one oval.

	1	2	3	4	5	6	7	8	9	
Lowest importance	◯	◯	◯	◯	◯	◯	◯	◯	◯	Highest importance

14. Unable to score/comments

Results from round 2:

15. Your answer for round 3: Odour related to the ear

Mark only one oval.

	1	2	3	4	5	6	7	8	9	
Lowest importance	◯	◯	◯	◯	◯	◯	◯	◯	◯	Highest importance

16. Unable to score/comments

Results from round 2:

17. Your answer for round 3: Squamous debris/keratin

Mark only one oval.

	1	2	3	4	5	6	7	8	9	
Lowest importance	◯	◯	◯	◯	◯	◯	◯	◯	◯	Highest importance

18. Unable to score/comments

Section 2: Core outcome set for acute otitis externa

Currently there is no accepted set of outcome measures used in studies of patients with acute otitis externa (AOE). This can lead to difficulty in analysing and interpreting clinical studies, which limits the effectiveness of our treatment of patients with AOE .

We'd like to establish which features of AOE should be included in a Core Outcome Set (COS) for use in all future studies of acute otitis externa (AOE).

The development of a Core Outcome Set (COS) first involves determining WHAT should be measured as an outcome, later establishing HOW this should be measured. The following questions hope to identify WHAT features should be measured.

Consensus results from round 2

In round 2, a further 11 of the 43 items reached consensus amongst professionals.

26 of the 43 items have now unambiguously reached consensus between patients and professionals, to be included in the CORE OUTCOME SET for AOE.

INCLUDED outcomes:
...on the number/frequency of visits to ENT or the GP
...on the need for pain relief
...on the need for overnight or in-hospital care
...on the need for antibiotics
...on the ease of applying the treatment
...on results of bacterial or fungal growth on an ear swab
...on patient satisfaction with their access to appropriate care (for diagnosis & treatment)
...on patient satisfaction with the required frequency of the treatments
...on patient satisfaction with the length of their symptoms
...on patient satisfaction with length of treatment, including number of visits to specialist services (ENT)
...on improvements in itchiness
...on improvements in hearing impairment
...on improvements in ear pain (otalgia)
...on improvements in ear canal swelling (oedema)
...on improvements in ear canal redness (erythema)
...on improvements in discharge from the ear (otorrhoea)
...on improvements in any pain on pressing just in front of the ear canal (tragal tenderness)
...on any widespread (systemic) side effects of treatments
...on any time off work
...on any spread of infection beyond the ear canal
...on any local side effects of treatments
...on any impact on quality of life
...if patients were compliant with the treatment
...if patients completed the course of treatment
...on the number of times the treatment is administered
…the time to resolution of symptoms

EXCLUDED outcome:
- No outcomes have been unambiguously excluded

Round 3

17 COS items remain undecided amongst professionals and patients for round 3.

You will now be shown 2 graphs, one of patient responses and of professional responses.

Please rate these OUTCOMES as to whether they could be INCLUDED or EXCLUDED in an ESSENTIAL outcome set for trials in acute otitis externa [17 outcomes]

Patients' responses from round 2: [no consensus]

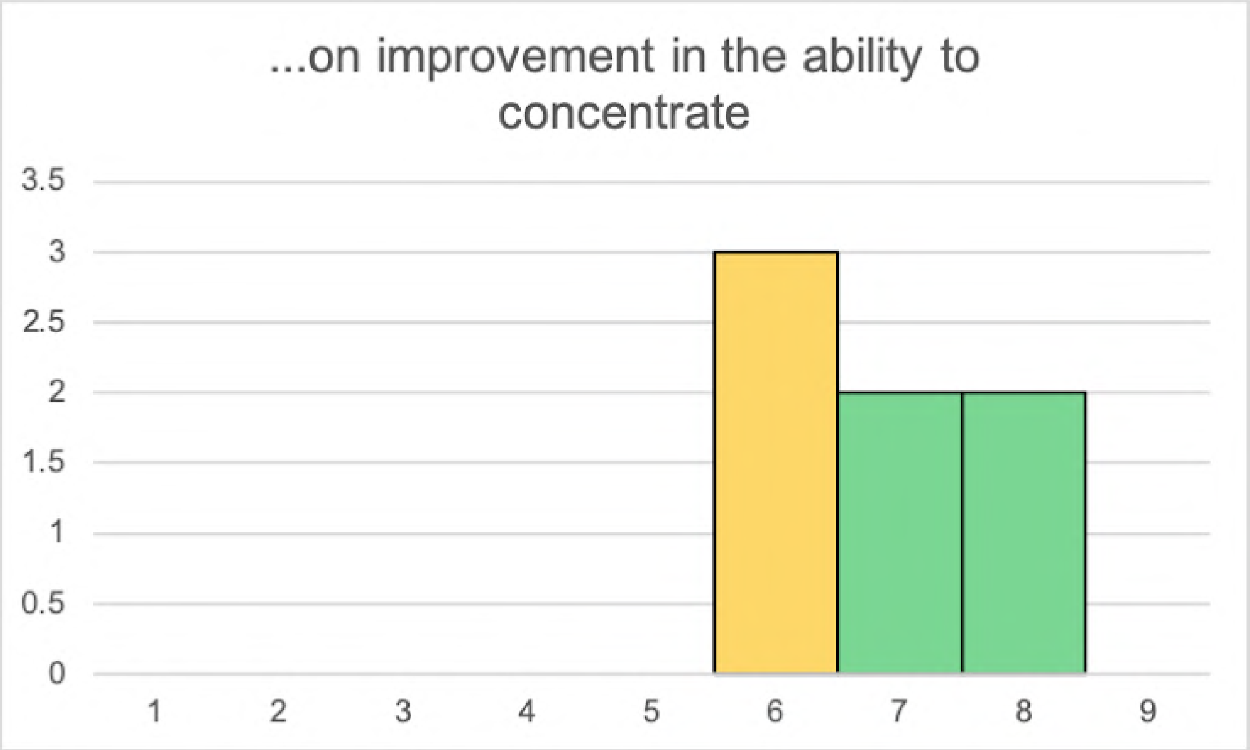

Professionals' responses from round 1: [excluded by professionals]

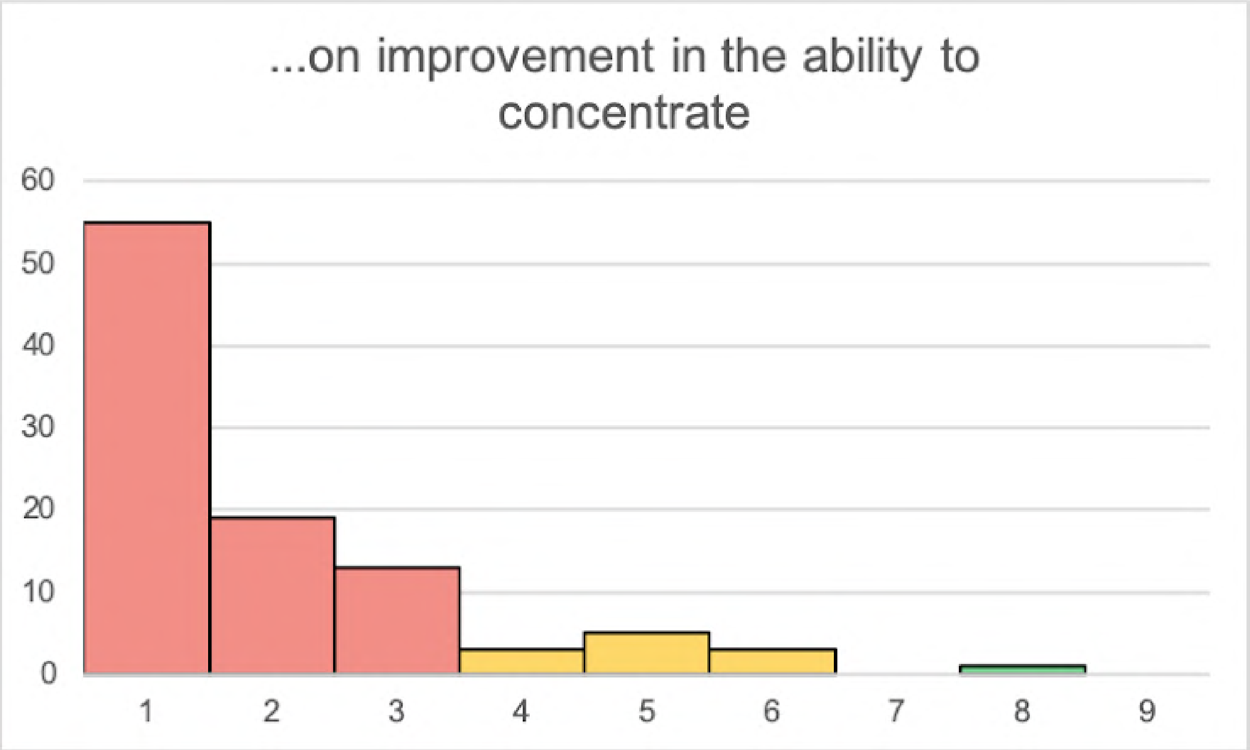

19. Your answer for round 3: Studies should report ...on improvement in the ability to concentrate

Mark only one oval.

	1	2	3	4	5	6	7	8	9	
Lowest importance	◯	◯	◯	◯	◯	◯	◯	◯	◯	Highest importance

20. Unable to score/comments

Patients' responses from round 2: [no consensus]

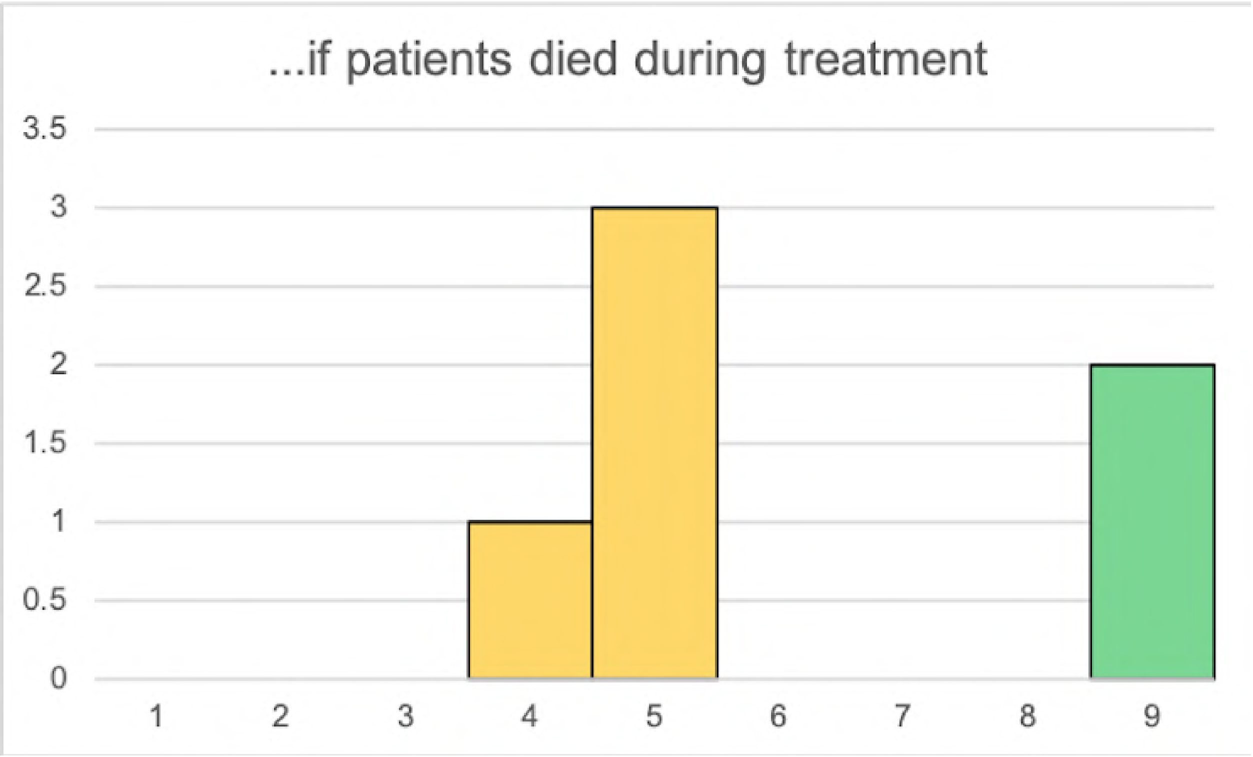

Professionals' response from round 2: [no consensus]

21. Your answer for round 3: Studies should report ...if patients died during treatment

Mark only one oval.

	1	2	3	4	5	6	7	8	9	
Lowest importance	⬭	⬭	⬭	⬭	⬭	⬭	⬭	⬭	⬭	Highest importance

22. Unable to score/comments

Patients' responses from round 2: [no consensus]

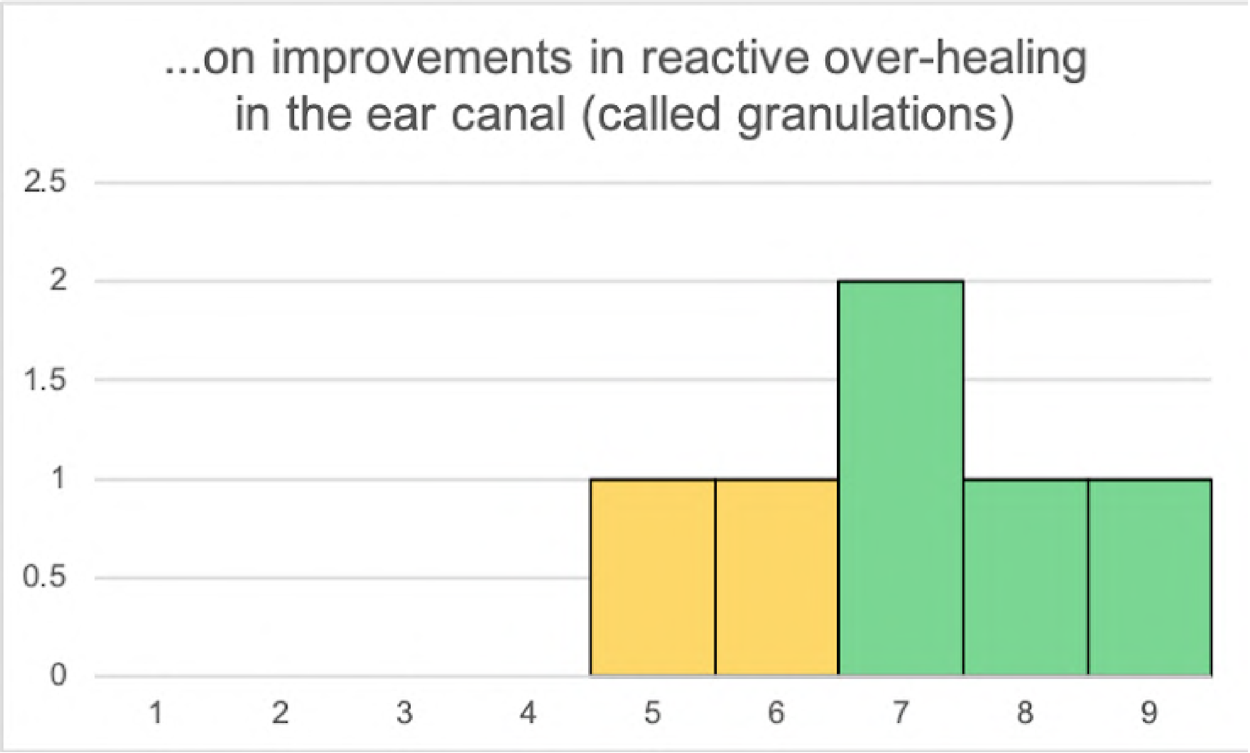

Professionals' responses from round 1: [included by professionals]

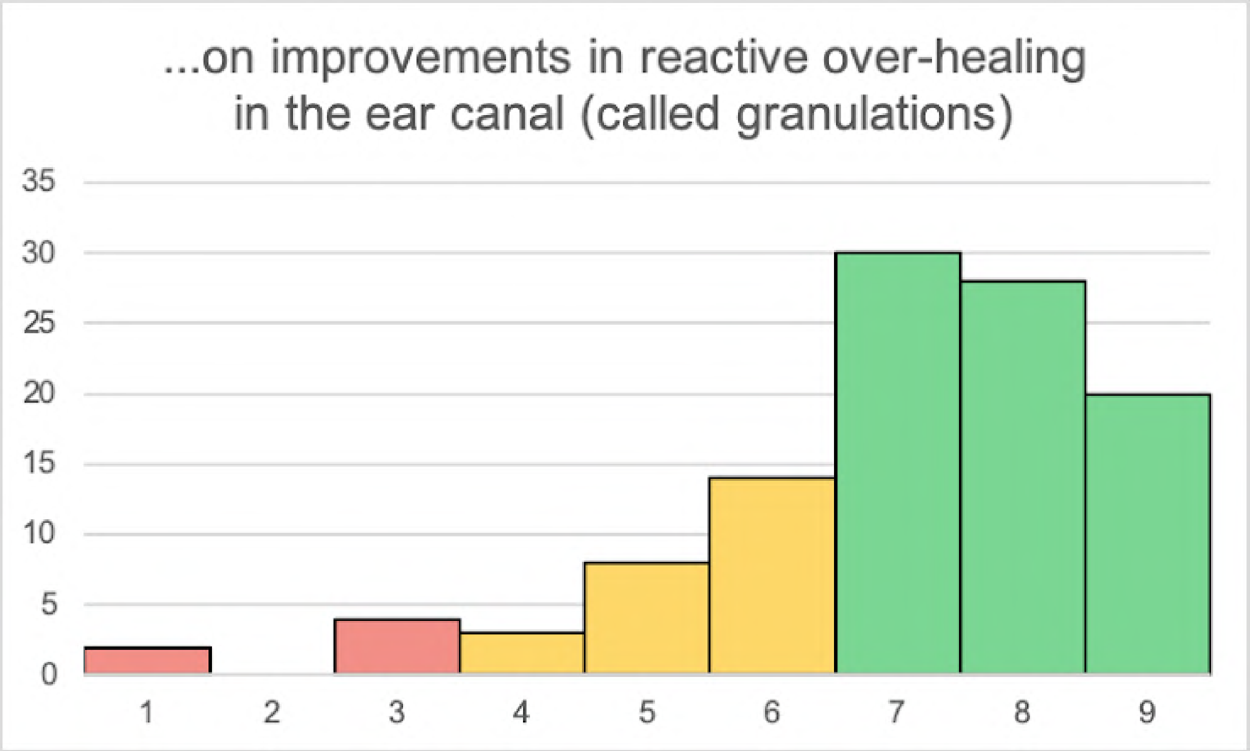

23. Your answer for round 3: Studies should report ...on improvements in reactive over-healing in the ear canal (called granulations)

Mark only one oval.

	1	2	3	4	5	6	7	8	9	
Lowest importance	◯	◯	◯	◯	◯	◯	◯	◯	◯	Highest importance

24. Unable to score/comments

____

____

____

____

____

Patients' responses from round 1: [included by patients]

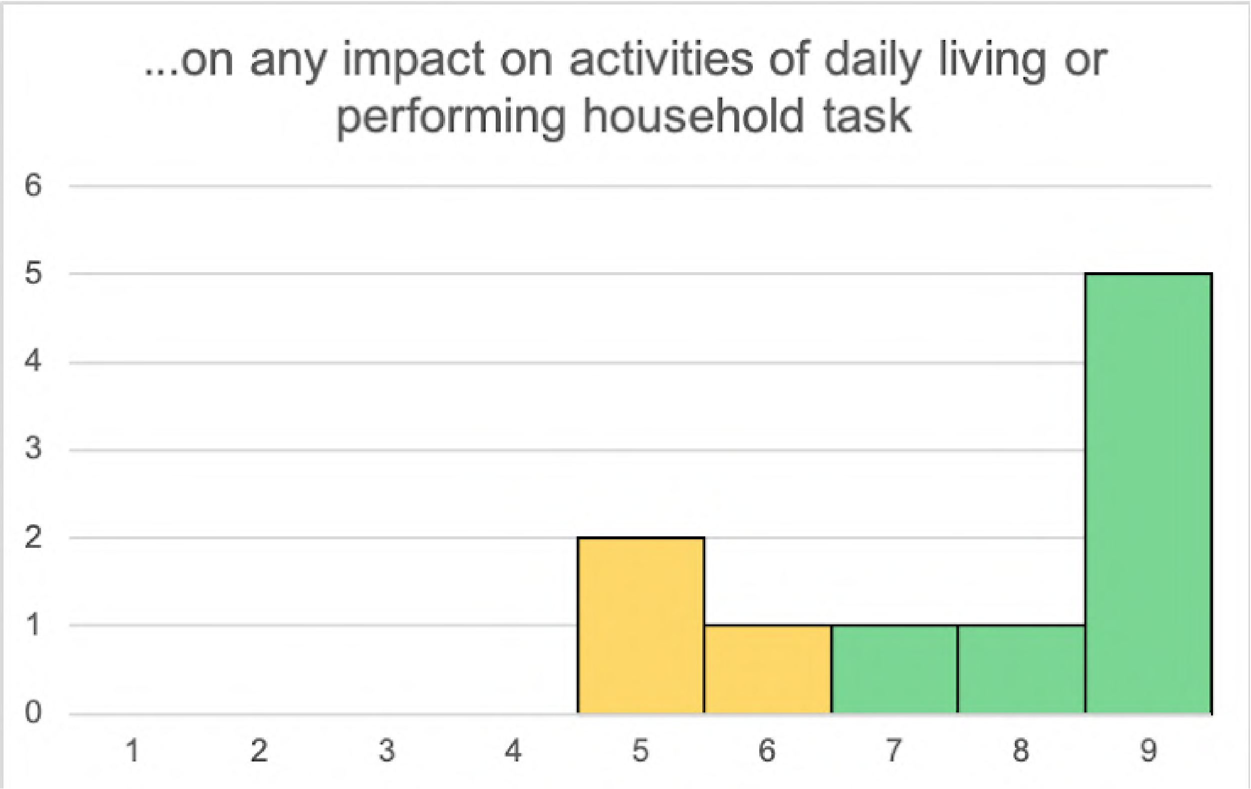

Professionals' responses from round 2: [no consensus]

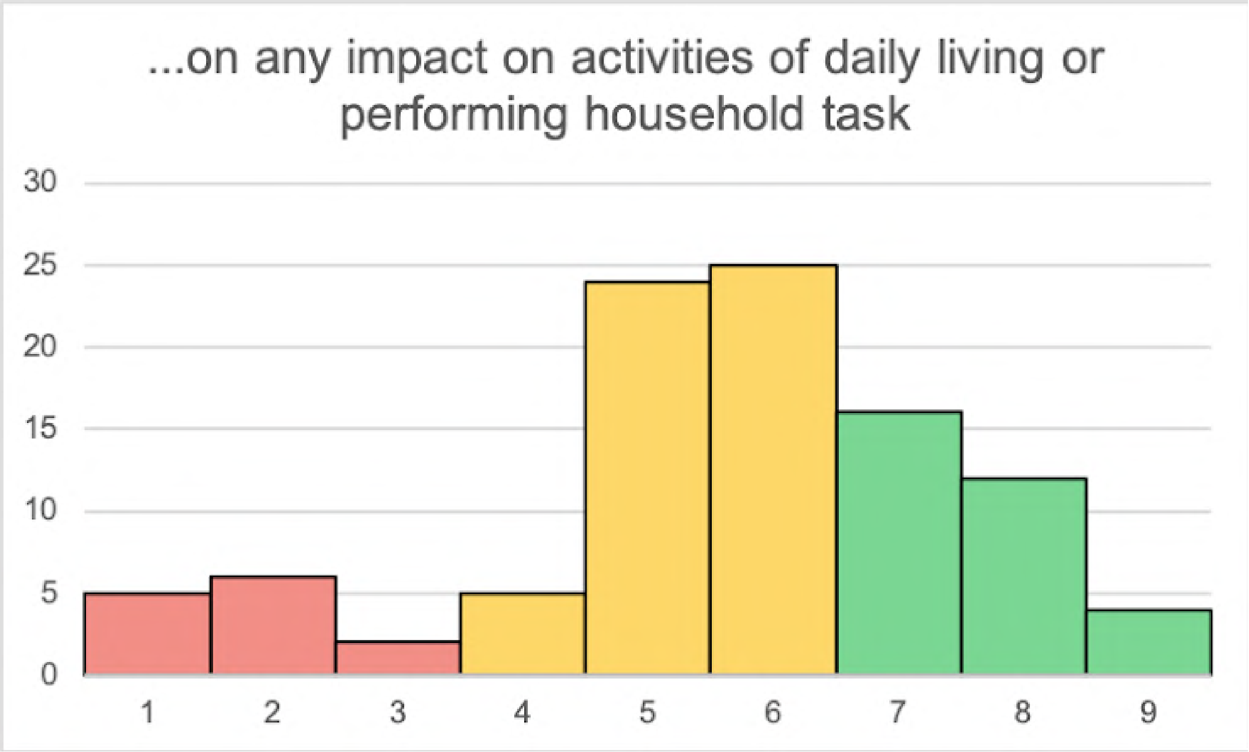

25. Your answer for round 3: Studies should report ...on any impact on activities of daily living or performing household task

Mark only one oval.

	1	2	3	4	5	6	7	8	9	
Lowest importance	◯	◯	◯	◯	◯	◯	◯	◯	◯	Highest importance

26. Unable to score/comments

Patients' responses from round 2: [no consensus]

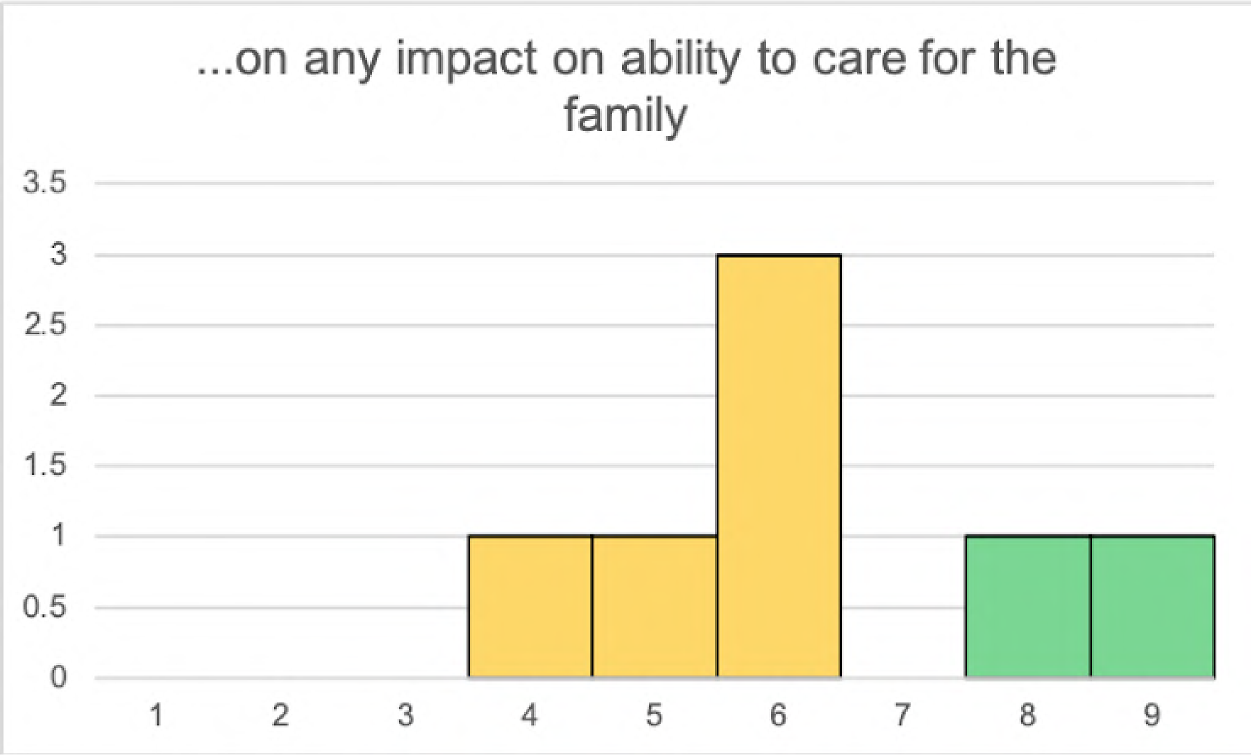

Professionals' responses from round 2: [no consensus]

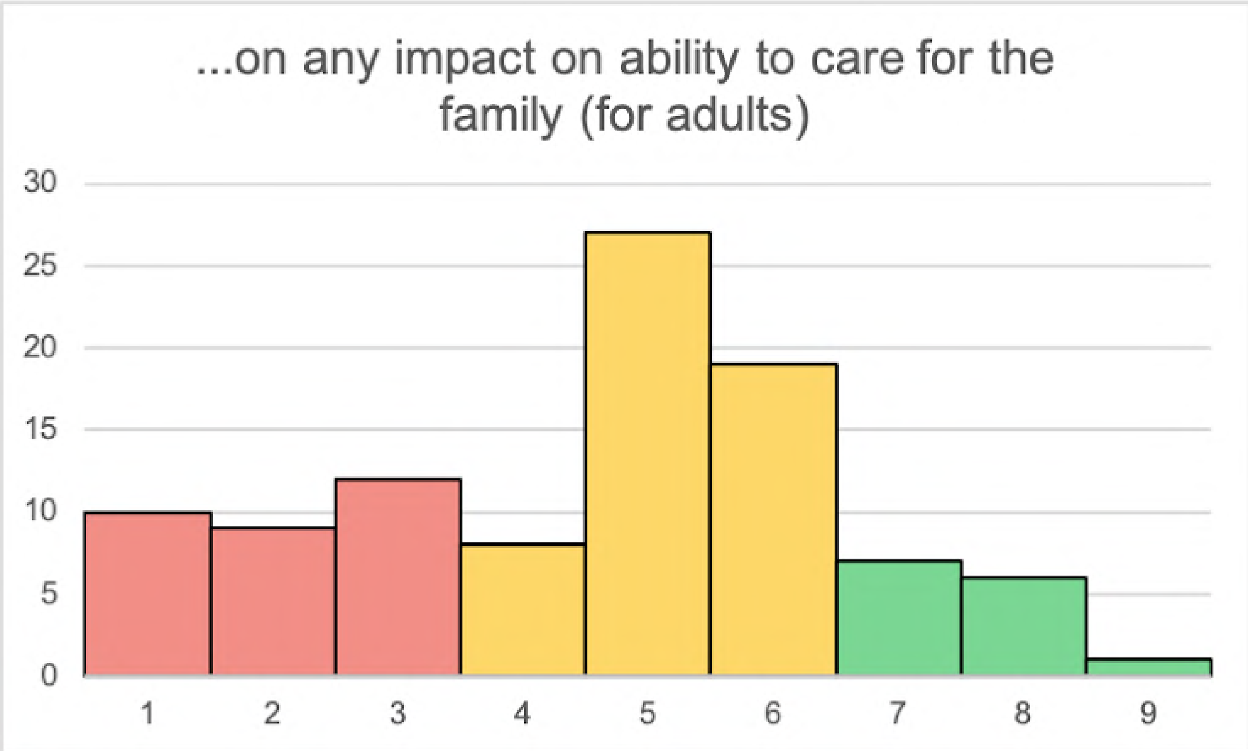

27. Your answer for round 3: Studies should report ...on any impact on ability to care for the family (for adults)

Mark only one oval.

	1	2	3	4	5	6	7	8	9	
Lowest importance	⬭	⬭	⬭	⬭	⬭	⬭	⬭	⬭	⬭	Highest importance

28. Unable to score/comments

[6-10] Studies should report...

Patients' responses from round 2: [no consensus]

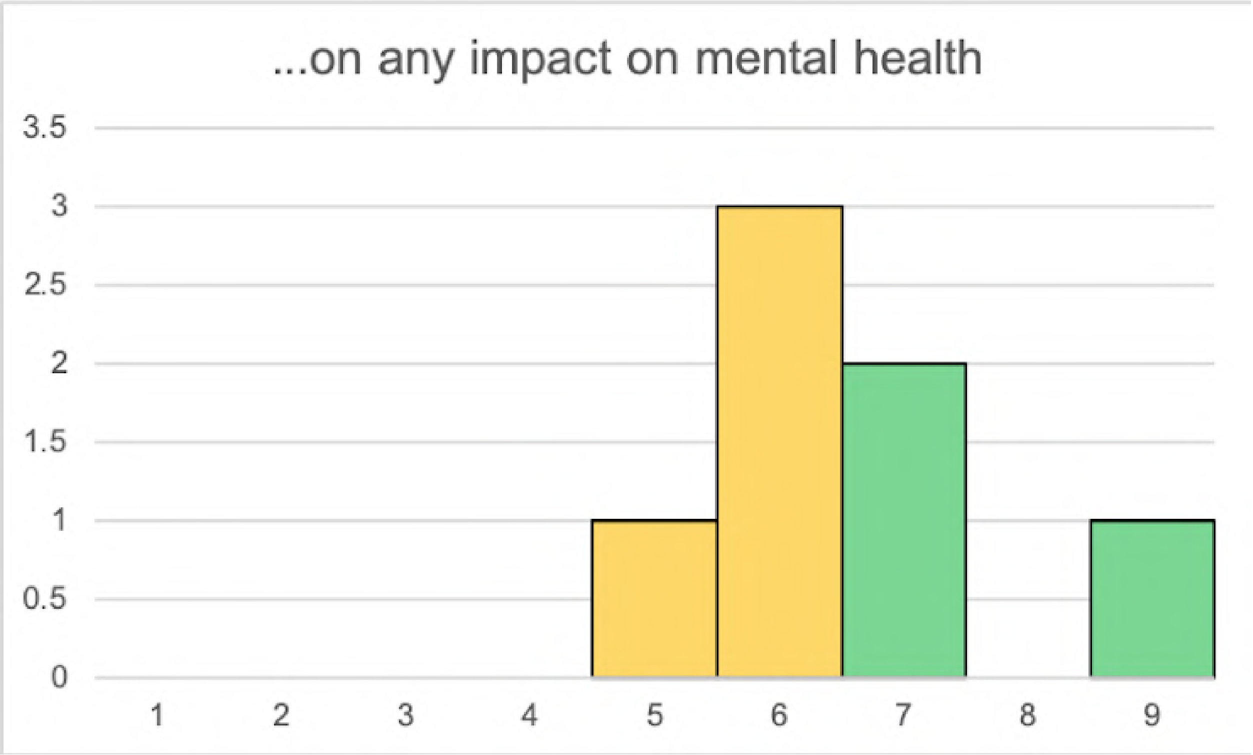

Professionals' responses from round 2: [no consensus]

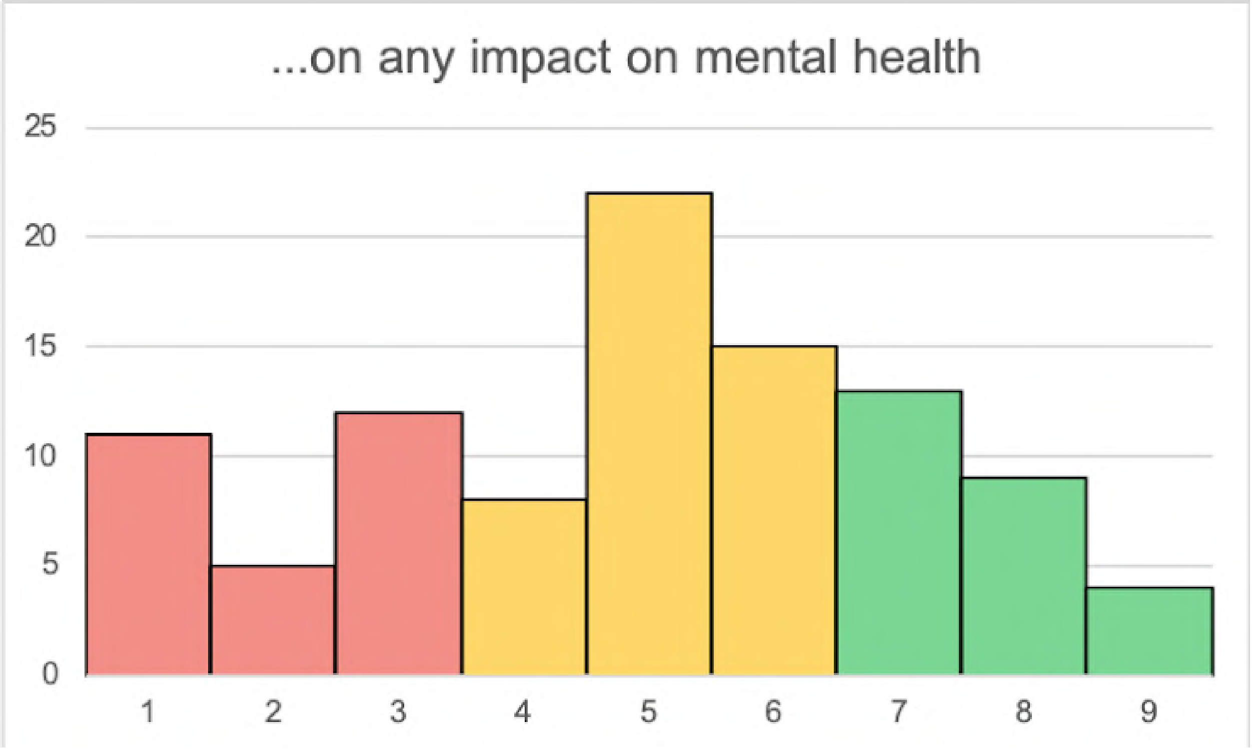

29. Your answer for round 3: Studies should report ...on any impact on mental health

Mark only one oval.

	1	2	3	4	5	6	7	8	9	
Lowest importance	⬭	⬭	⬭	⬭	⬭	⬭	⬭	⬭	⬭	Highest importance

30. Unable to score/comments

Patients' responses from round 1: [included by patients]

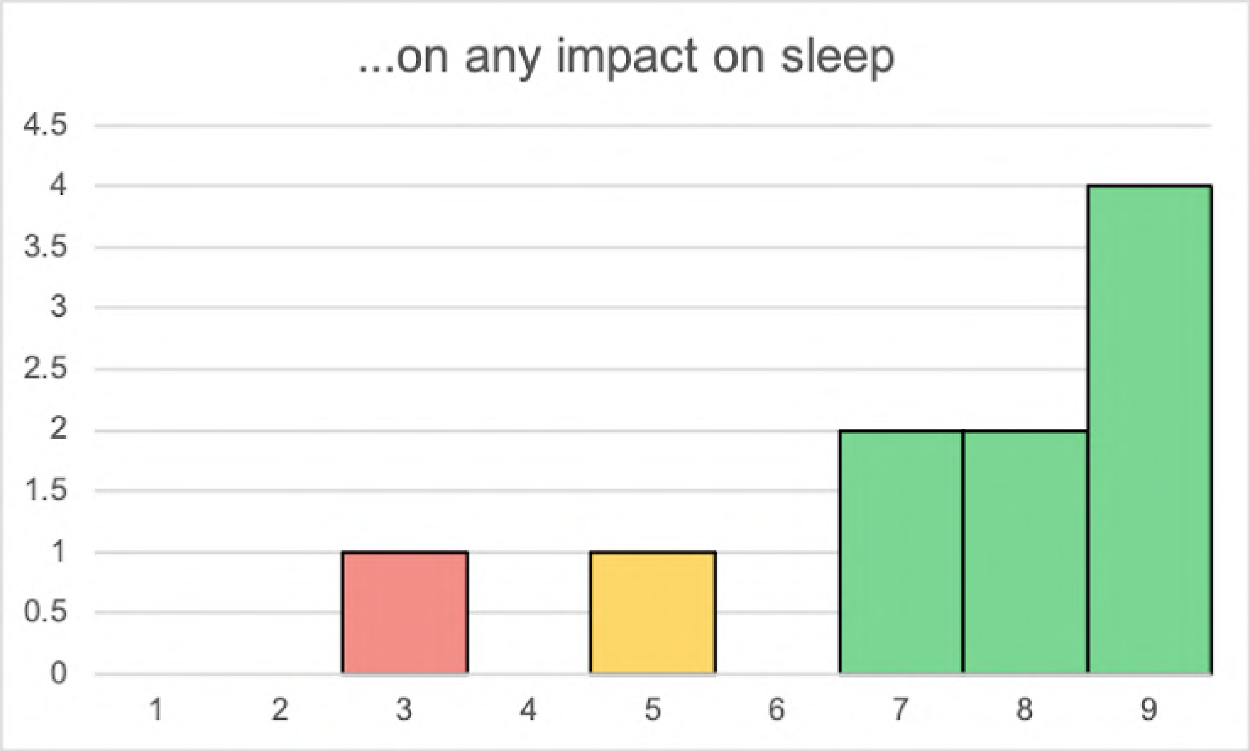

Professionals' responses from round 2: [no consensus]

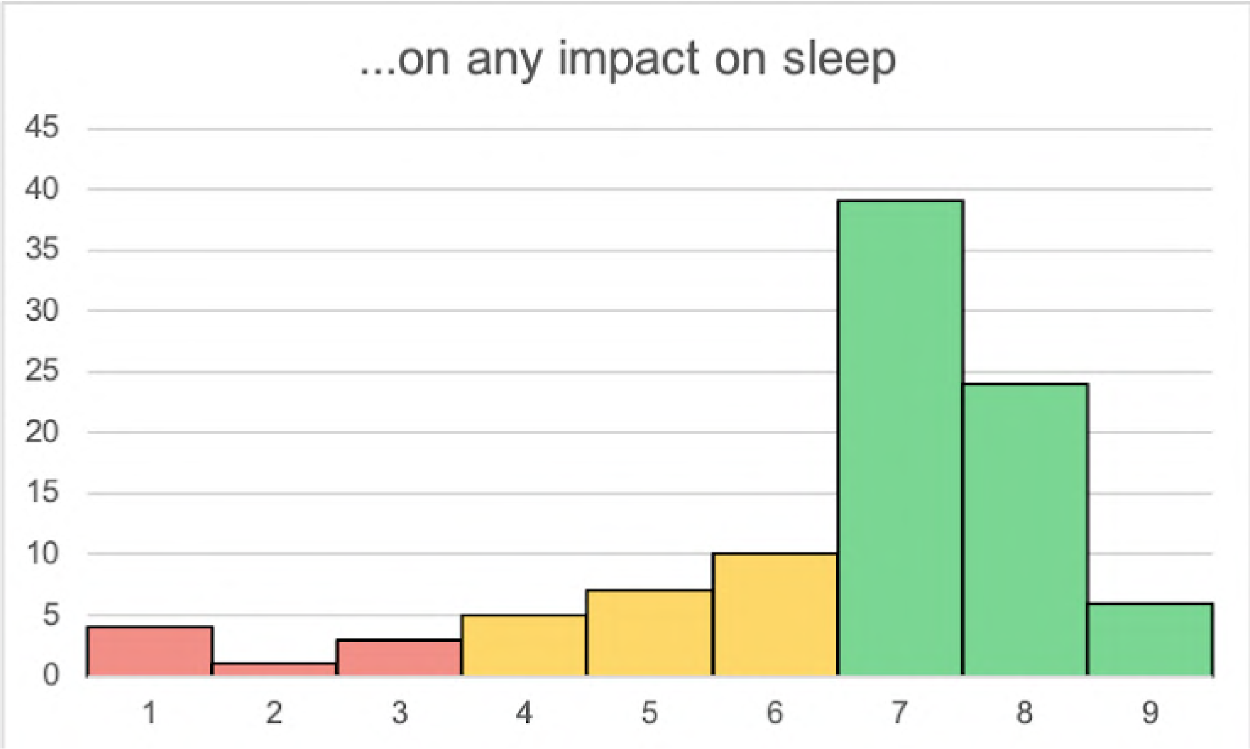

31. Your answer for round 3: Studies should report ...on any impact on sleep

Mark only one oval.

	1	2	3	4	5	6	7	8	9	
Lowest importance	◯	◯	◯	◯	◯	◯	◯	◯	◯	Highest importance

32. Unable to score/comments

Patients' responses from round 2: [included by patients]

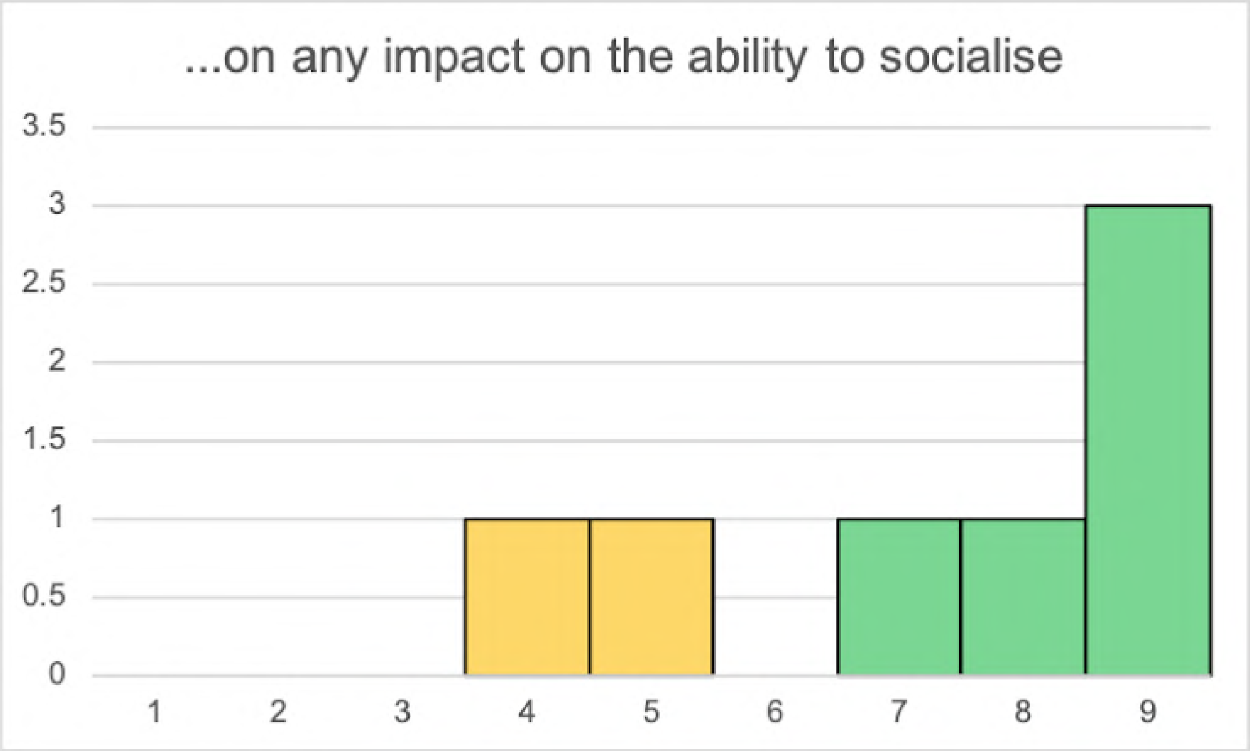

Professionals' responses from round 2: [no consensus]

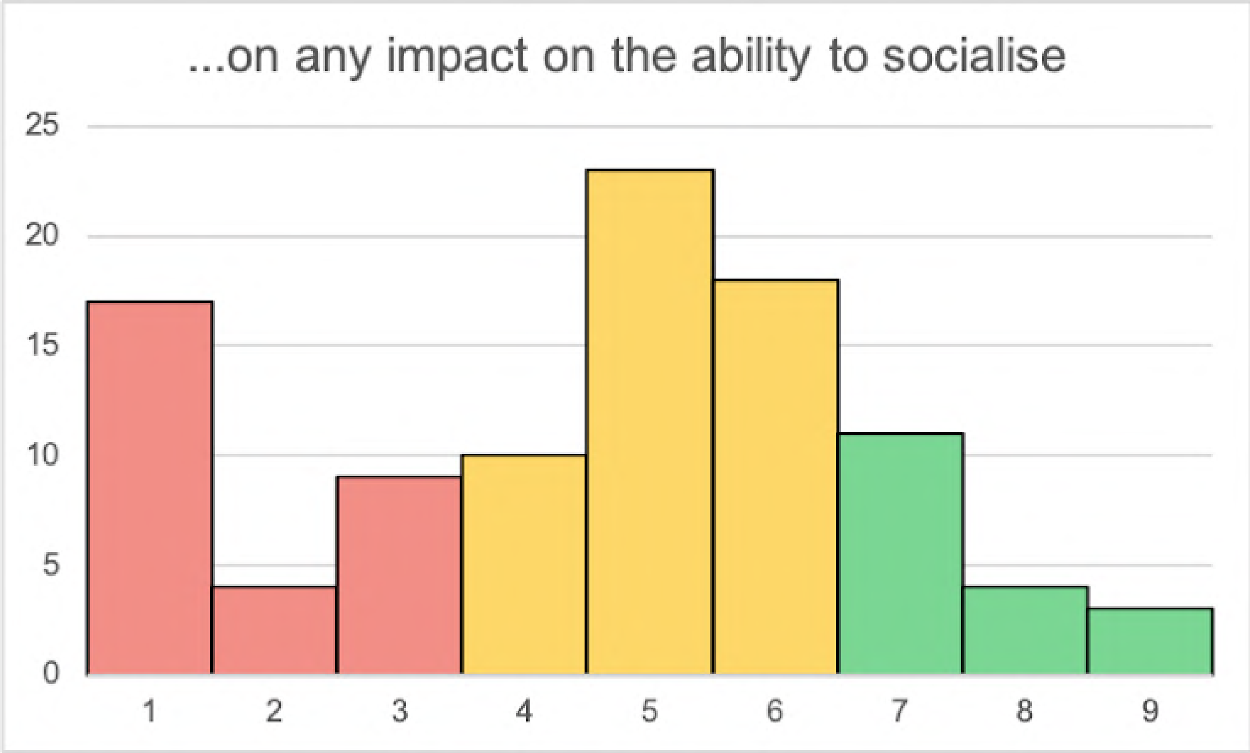

33. Your answer for round 3: Studies should report ...on any impact on the ability to socialise

Mark only one oval.

	1	2	3	4	5	6	7	8	9	
Lowest importance	⬭	⬭	⬭	⬭	⬭	⬭	⬭	⬭	⬭	Highest importance

34. Unable to score/comments

Patients' responses from round 2: [included by patients]

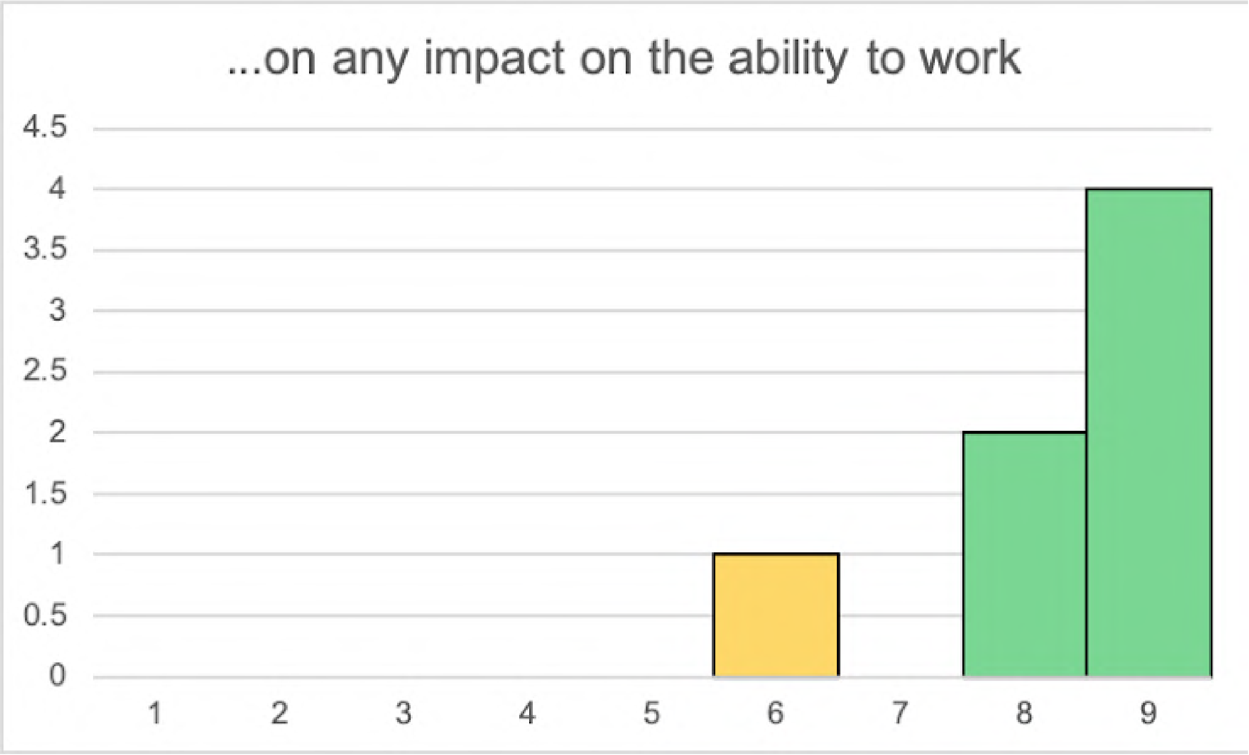

Professionals' responses from round 2: [no consensus]

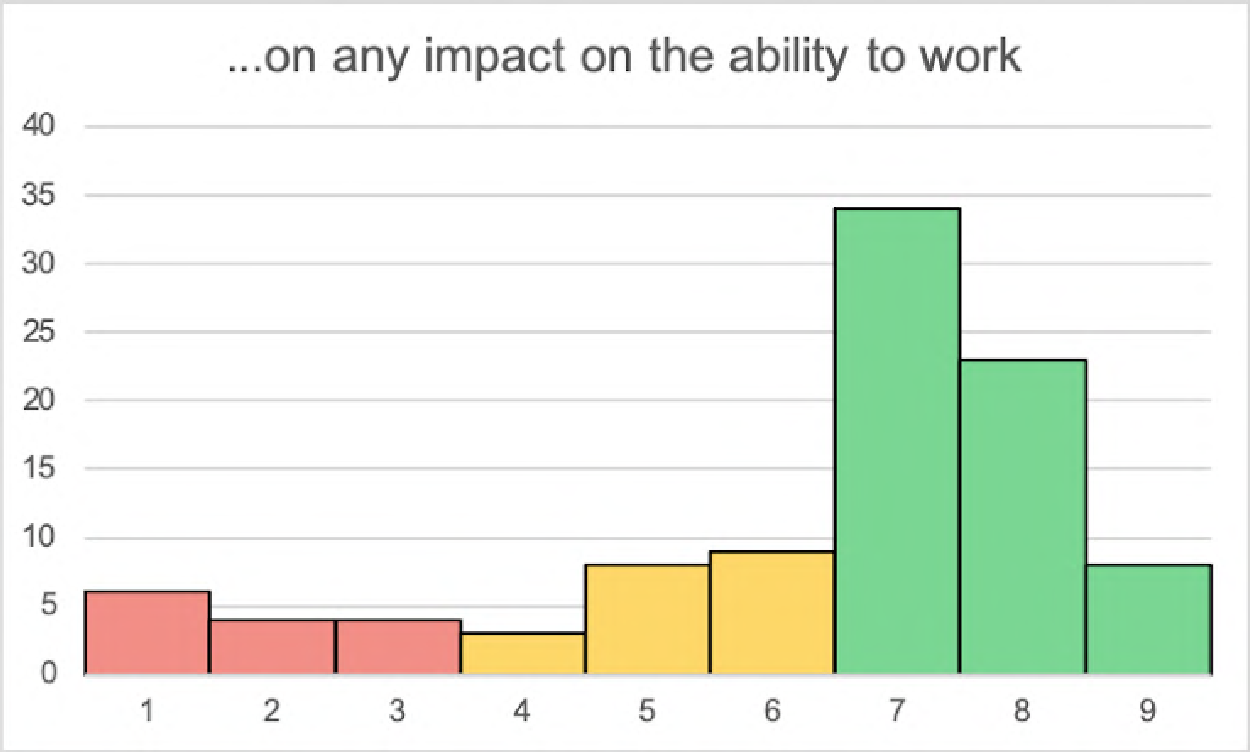

35. Your answer for round 3: Studies should report ...on any impact on the ability to work

Mark only one oval.

	1	2	3	4	5	6	7	8	9	
Lowest importance	◯	◯	◯	◯	◯	◯	◯	◯	◯	Highest importance

36. Unable to score/comments

Patients' responses from round 1: [included by patients]

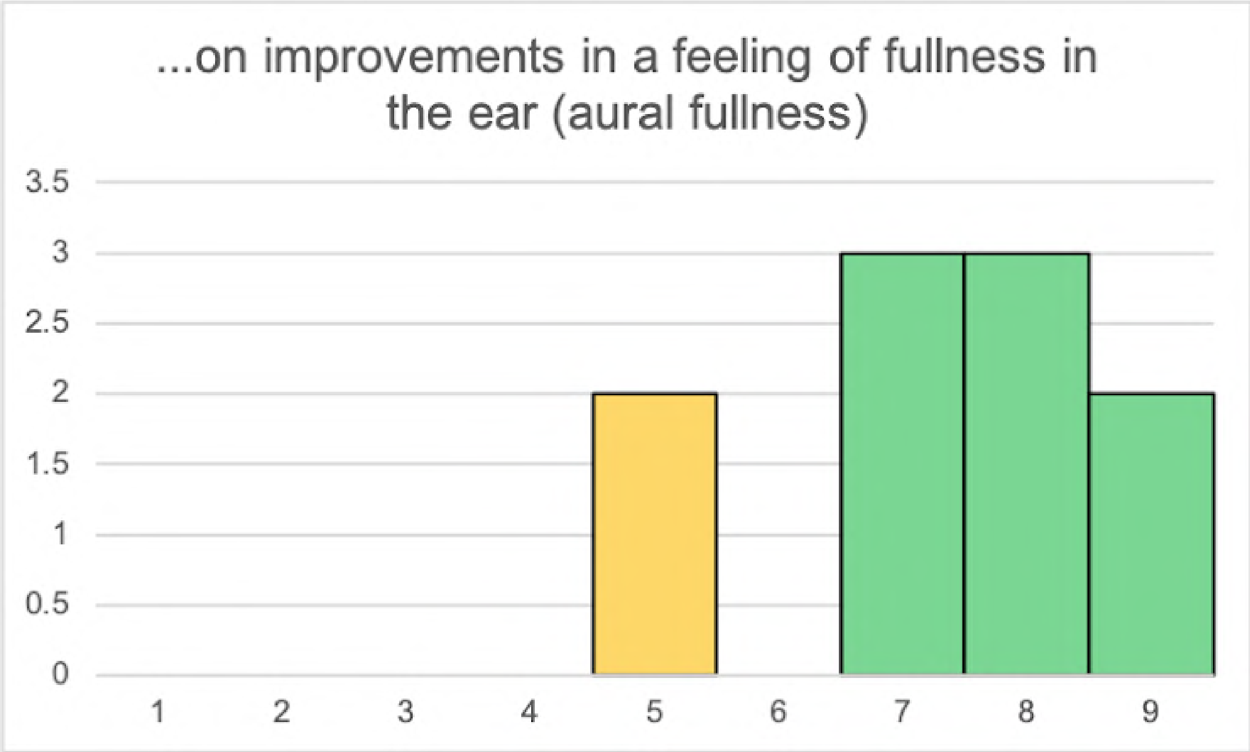

Professionals' responses from round 2: [no consensus]

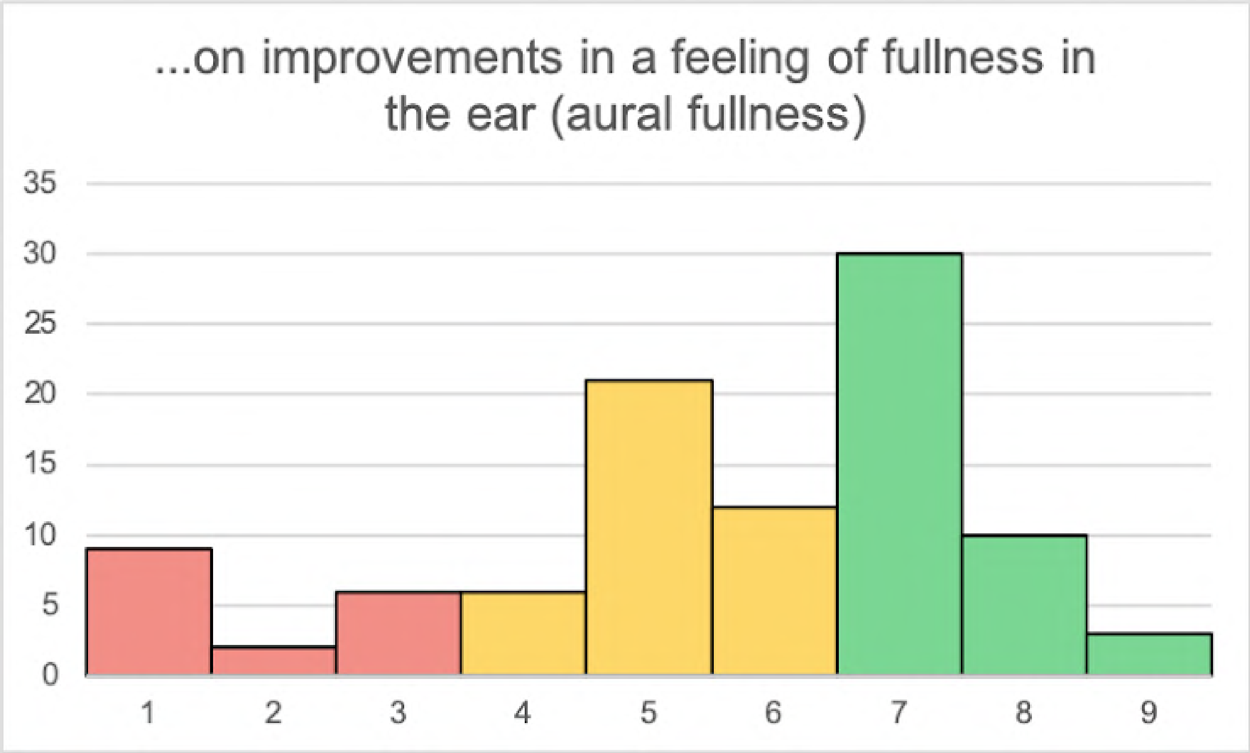

37. Your answer for round 3: Studies should report ...on improvements in a feeling of fullness in the ear (aural fullness)

Mark only one oval.

	1	2	3	4	5	6	7	8	9	
Lowest importance										Highest importance

38. Unable to score/comments

Patients' responses from round 2: [no consensus]

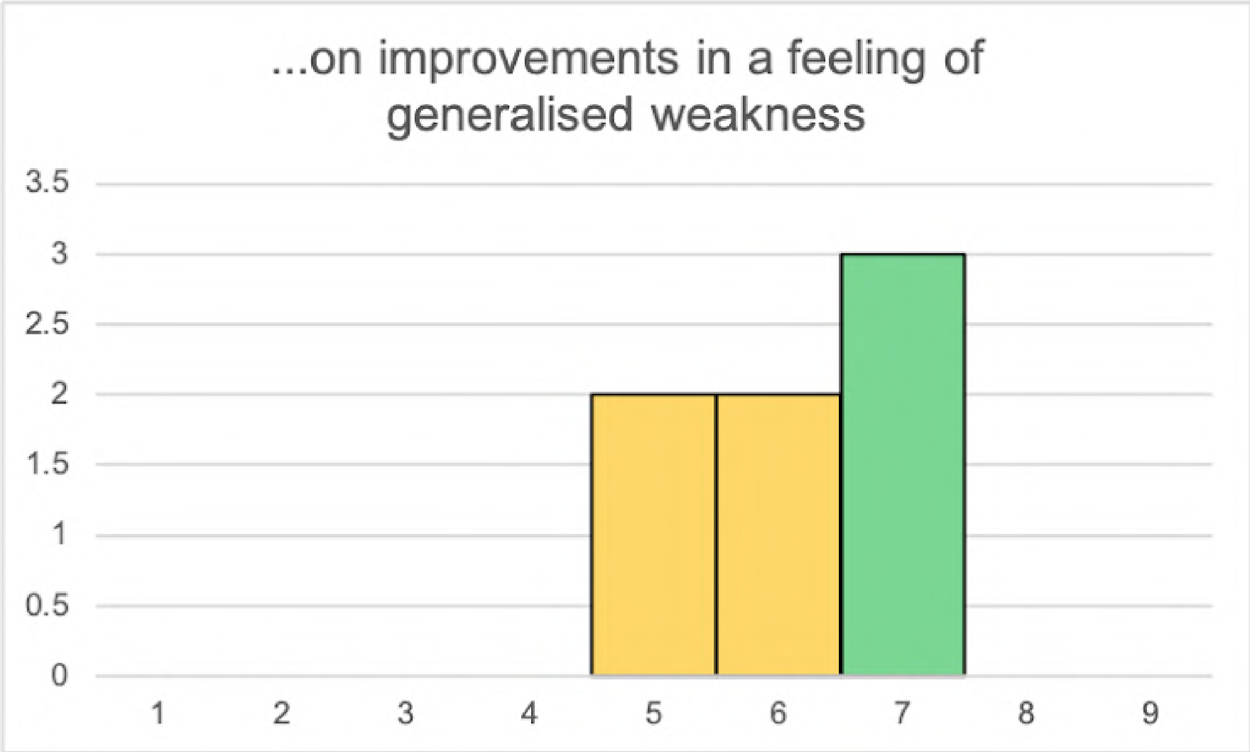

Professionals' responses from round 2: [no consensus]

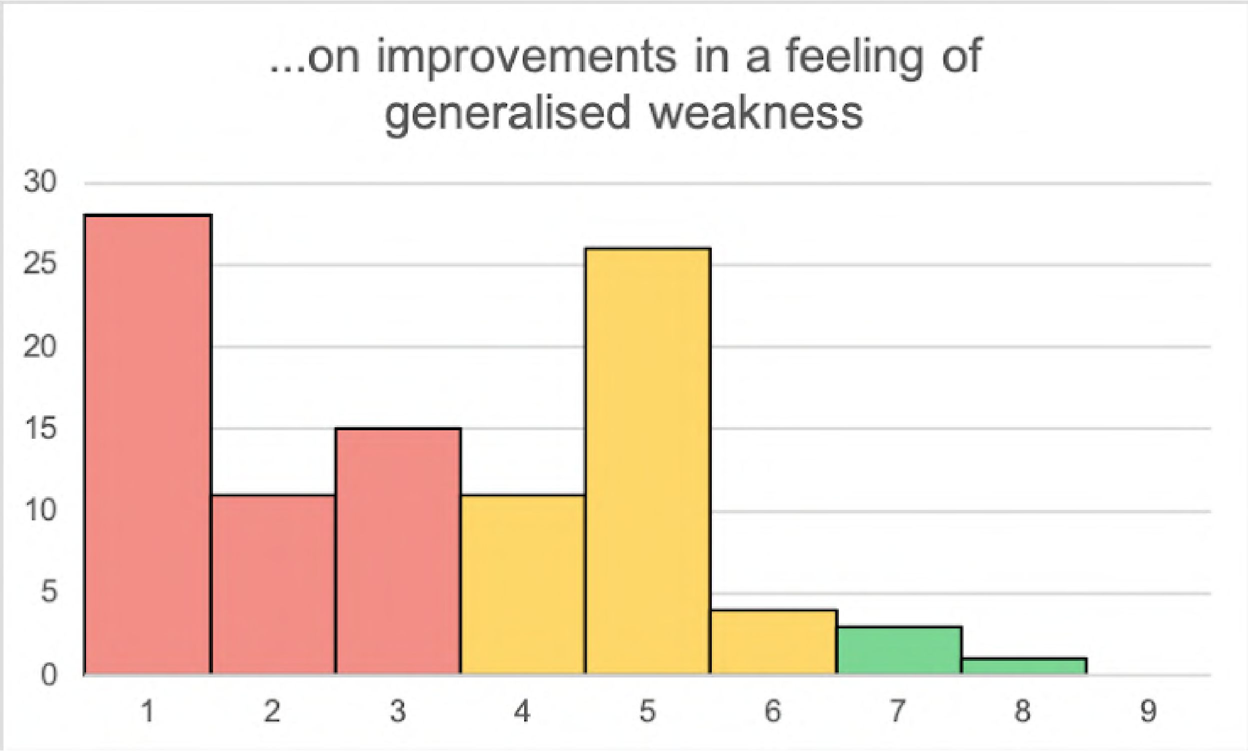

39. Your answer for round 3: Studies should report ...on improvements in a feeling of generalised weakness

Mark only one oval.

	1	2	3	4	5	6	7	8	9	
Lowest importance	⬭	⬭	⬭	⬭	⬭	⬭	⬭	⬭	⬭	Highest importance

40. Unable to score/comments

Patients' responses from round 2: [no consensus]

Professionals' responses from round 2: [no consensus]

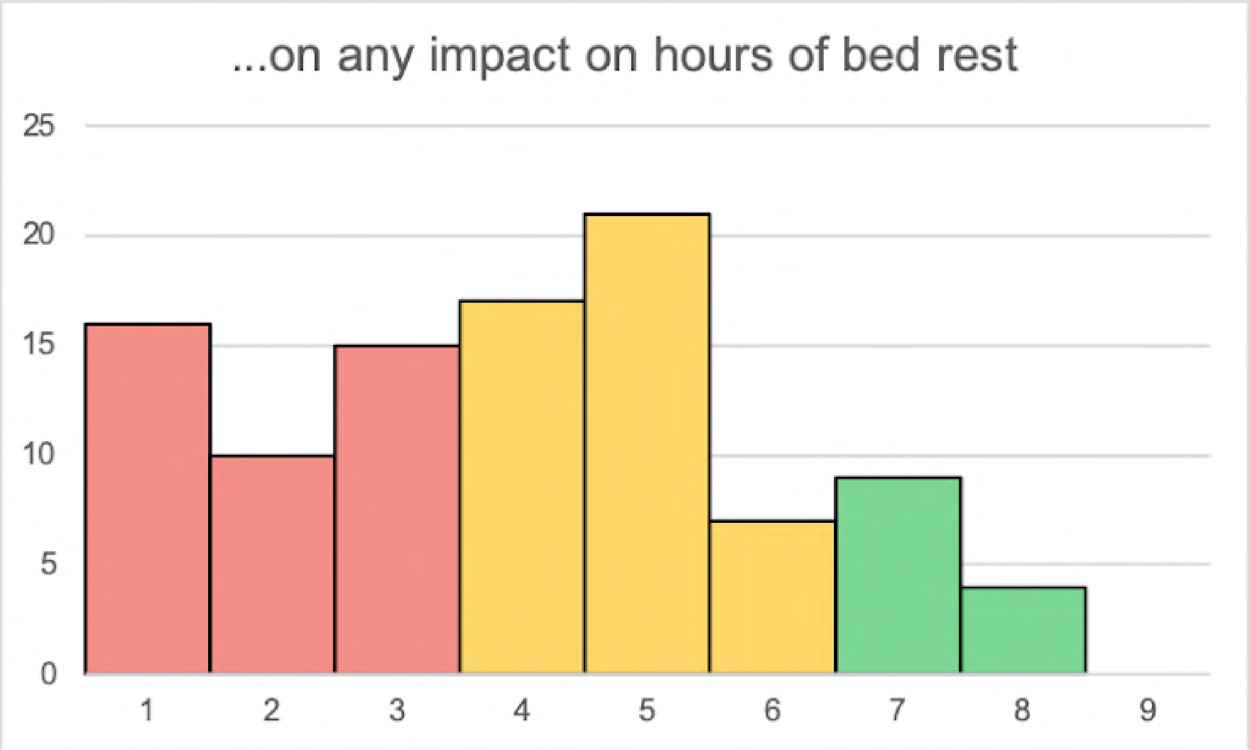

41. Your answer for round 3: Studies should report ...on any impact on hours of bed rest

Mark only one oval.

	1	2	3	4	5	6	7	8	9	
Lowest importance	◯	◯	◯	◯	◯	◯	◯	◯	◯	Highest importance

42. Unable to score/comments

Patients' responses from round 2: [no consensus]

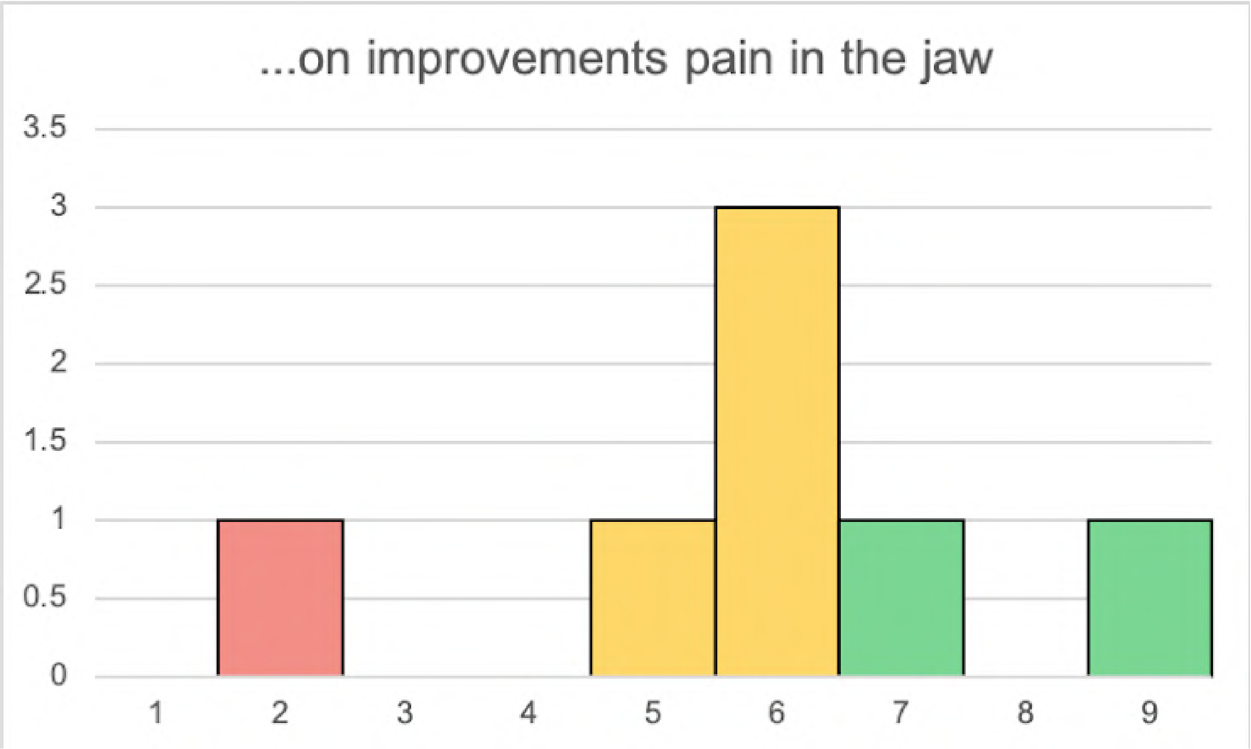

Professionals' responses from round 2: [no consensus]

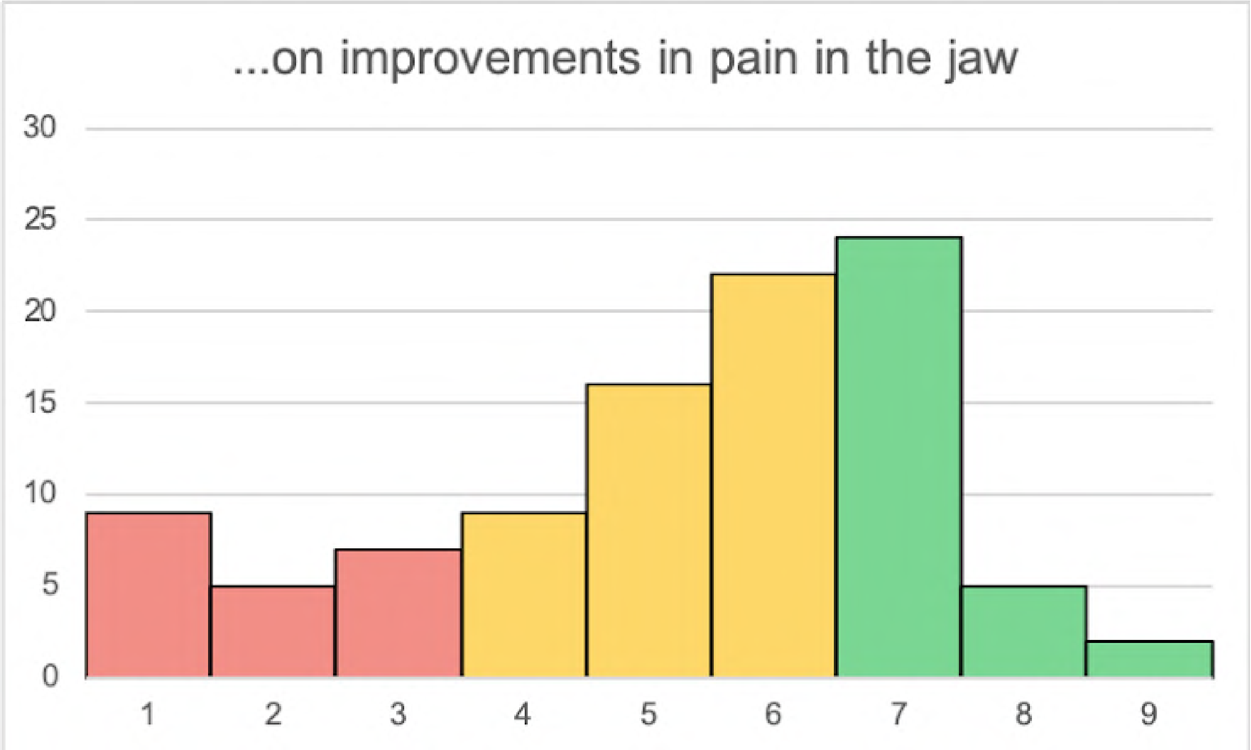

43. Your answer for round 3: Studies should report ...on improvements in pain in the jaw

Mark only one oval.

	1	2	3	4	5	6	7	8	9	
Lowest importance	⬭	⬭	⬭	⬭	⬭	⬭	⬭	⬭	⬭	Highest importance

44. Unable to score/comments

Patients' responses from round 2: [no consensus]

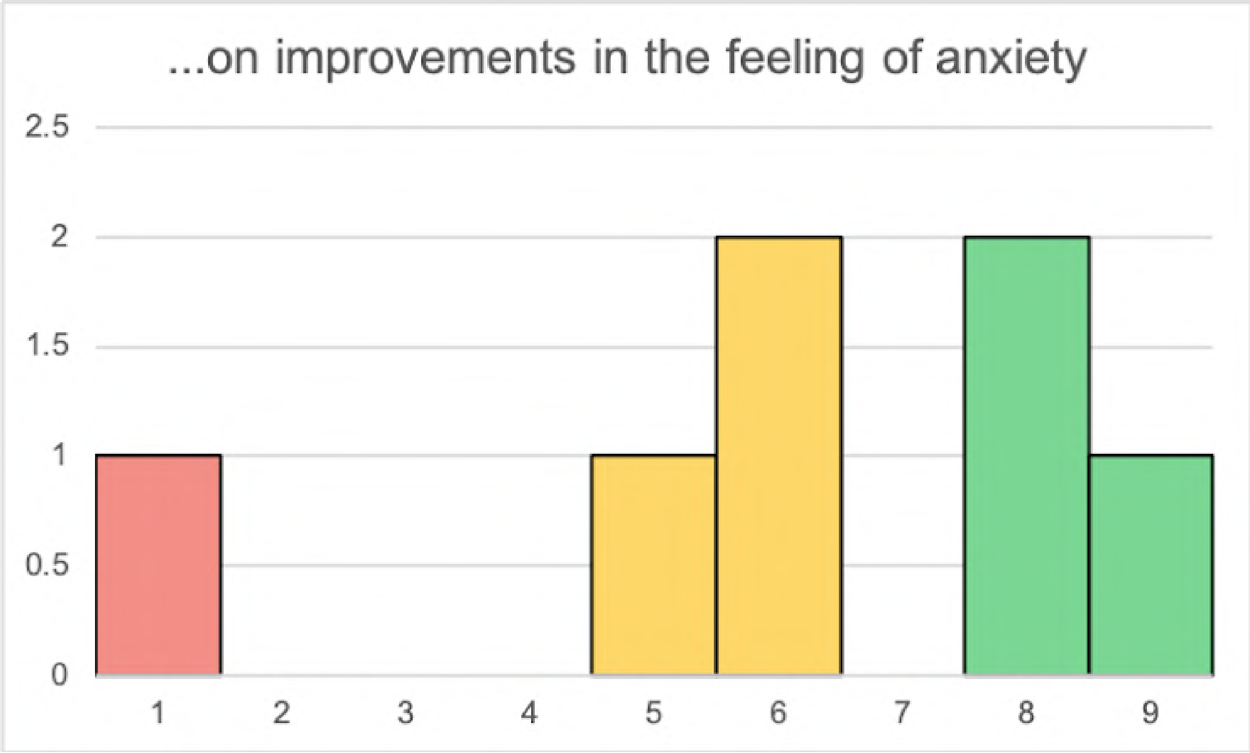

Professionals' responses from round 2: [no consensus]

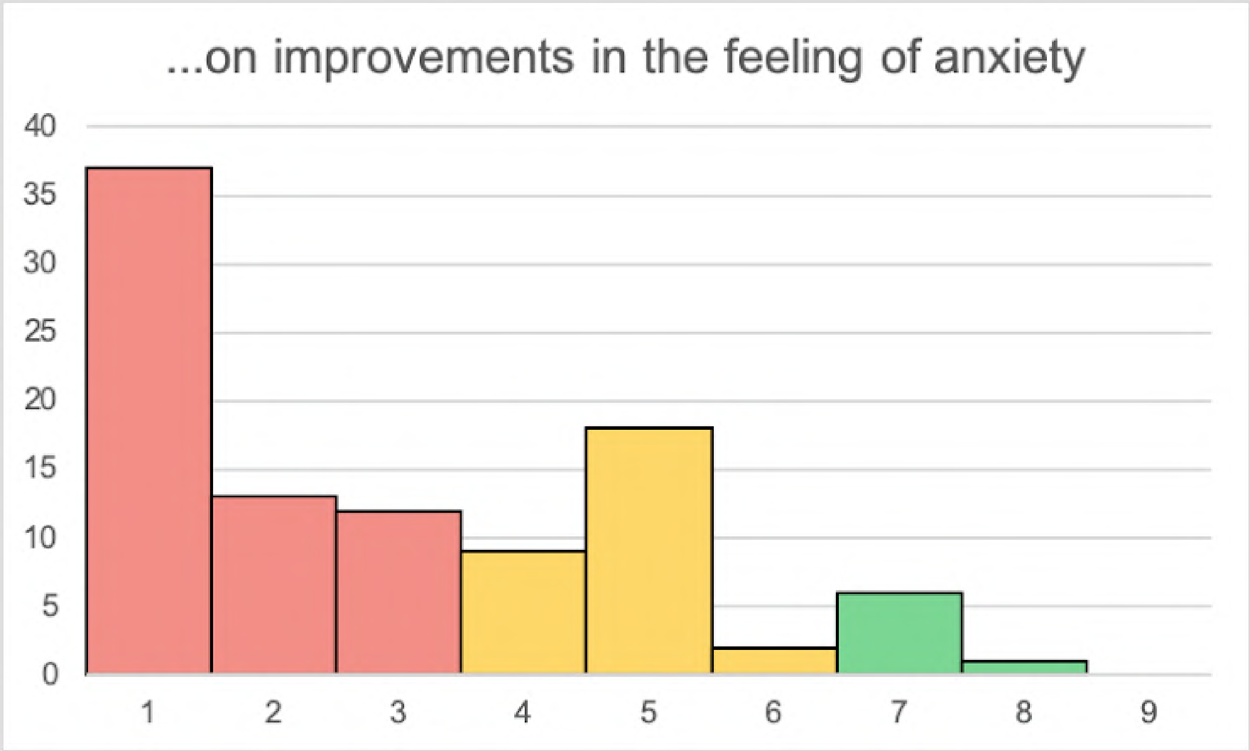

45. Your answer for round 3: Studies should report ...on improvements in the feeling of anxiety

Mark only one oval.

	1	2	3	4	5	6	7	8	9	
Lowest importance	◯	◯	◯	◯	◯	◯	◯	◯	◯	Highest importance

46. Unable to score/comments

Patients' responses from round 2: [included by patients]

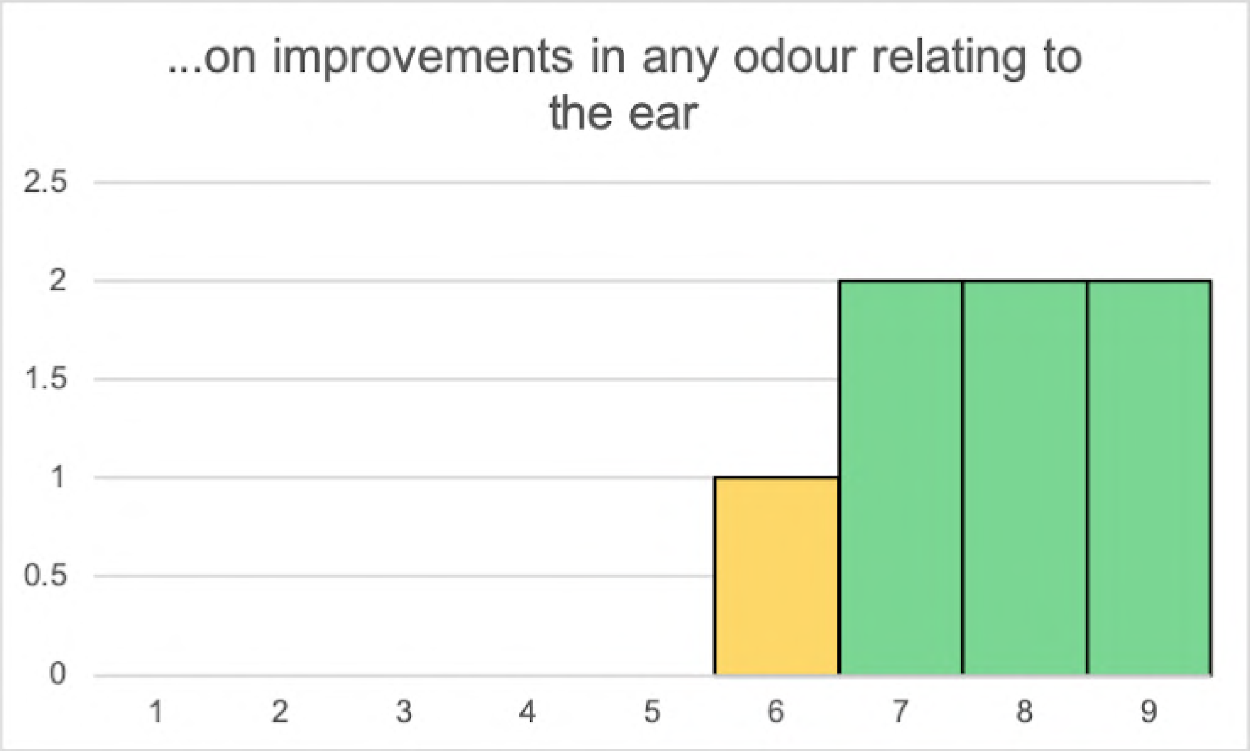

Professionals' responses from round 2: [no consensus]

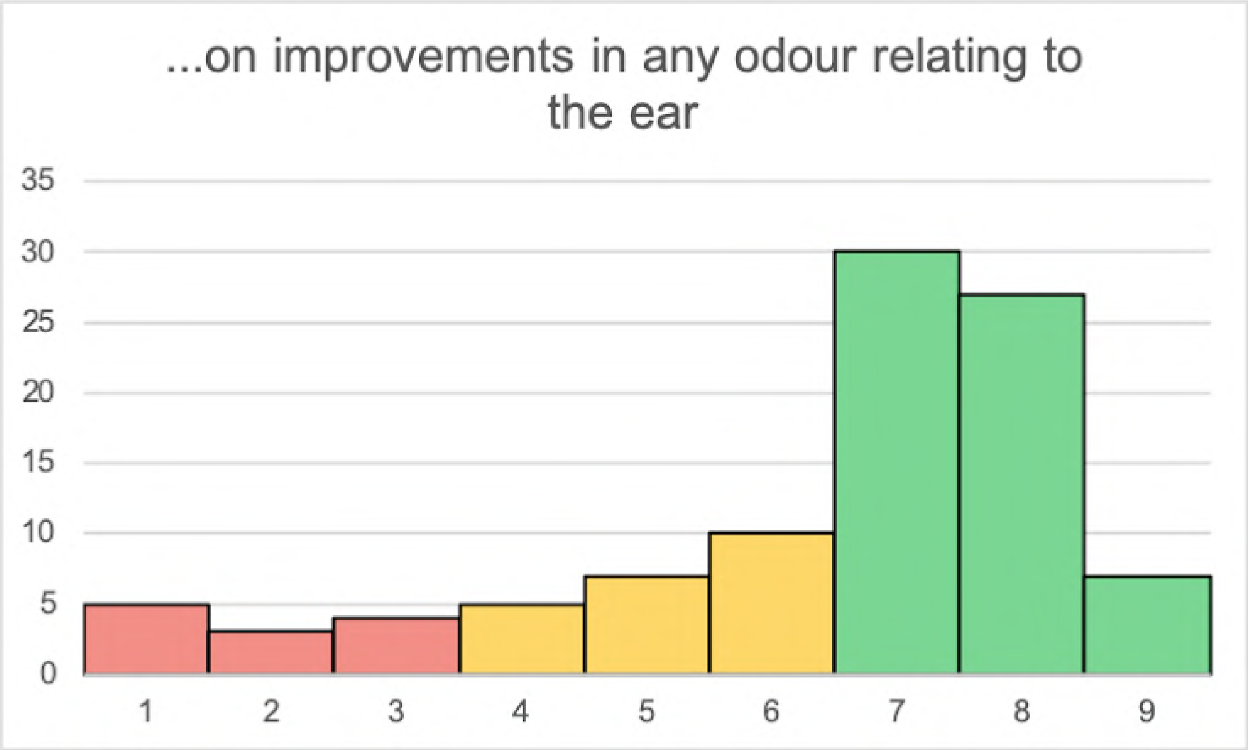

47. Your answer for round 3: Studies should report ...on improvements in any odour relating to the ear

Mark only one oval.

	1	2	3	4	5	6	7	8	9	
Lowest importance	◯	◯	◯	◯	◯	◯	◯	◯	◯	Highest importance

48. Unable to score/comments

Patients' responses from round 1: [included by patients]

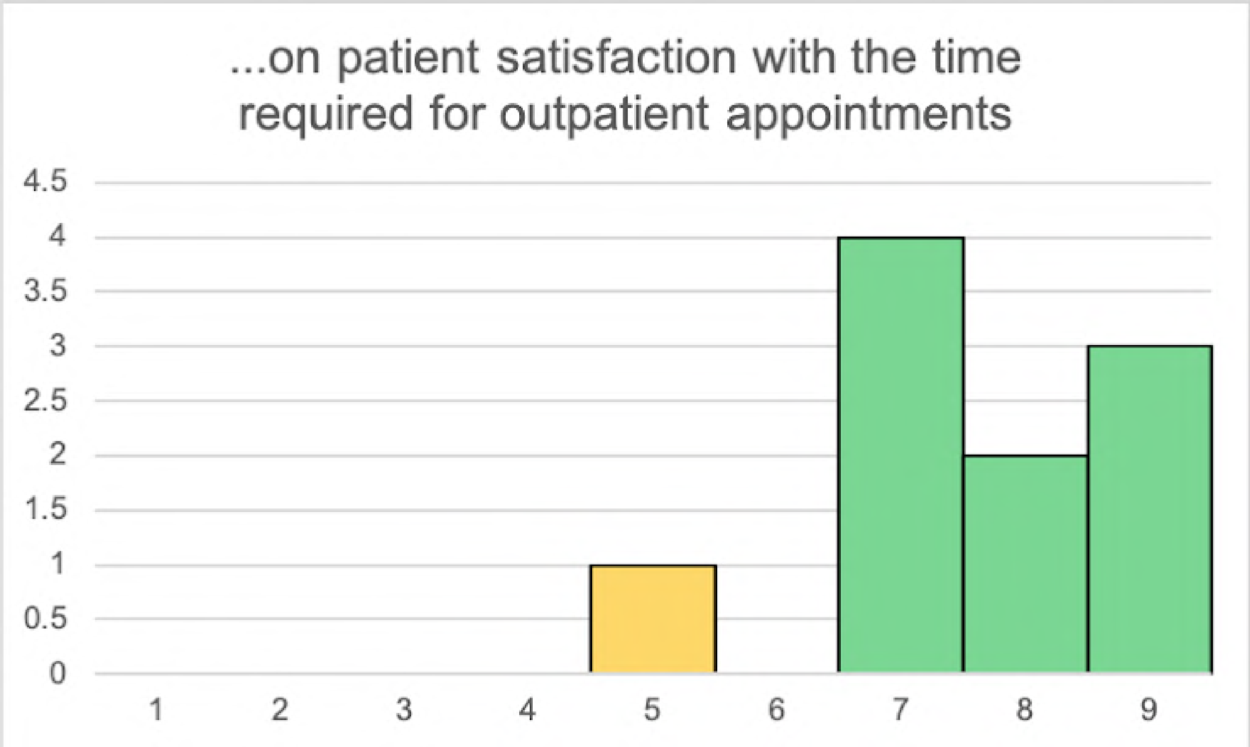

Professionals' responses from round 2: [no consensus]

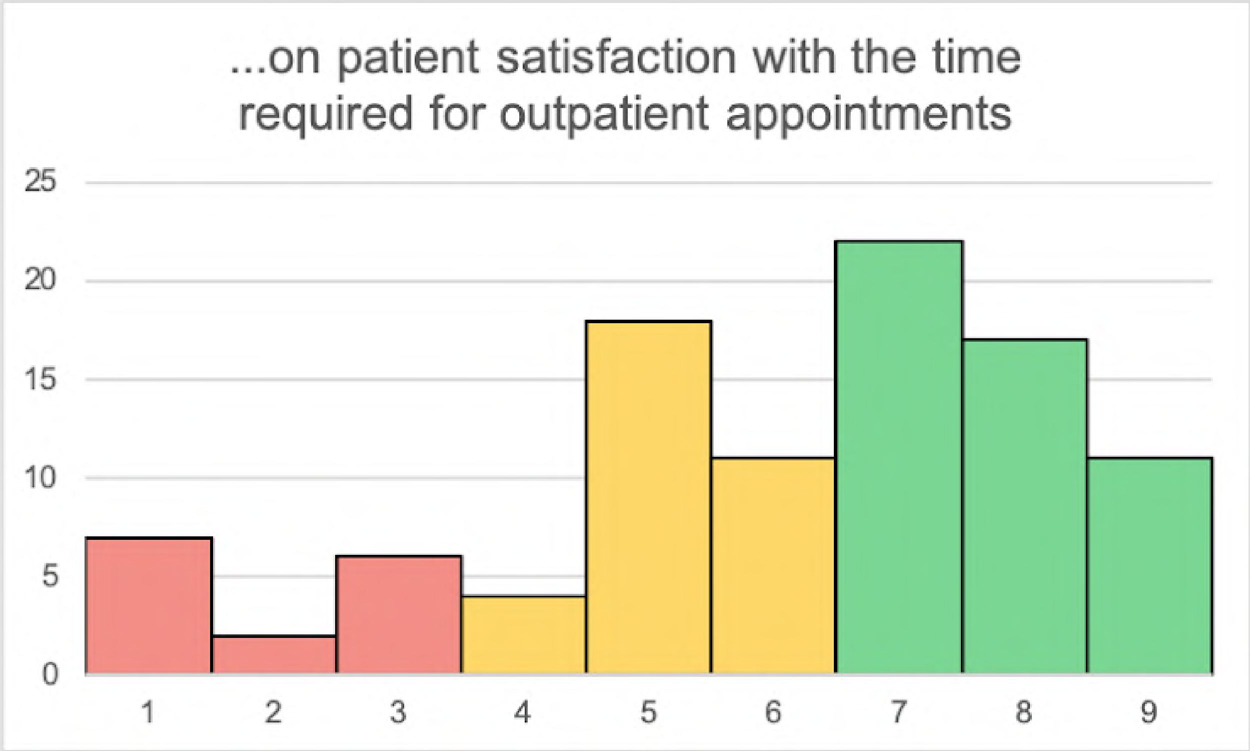

49. Your answer for round 3: Studies should report ...on patient satisfaction with the time required for outpatient appointments

Mark only one oval.

	1	2	3	4	5	6	7	8	9	
Lowest importance	⬭	⬭	⬭	⬭	⬭	⬭	⬭	⬭	⬭	Highest importance

50. Unable to score/comments

Patients' responses from round 2: [no consensus]

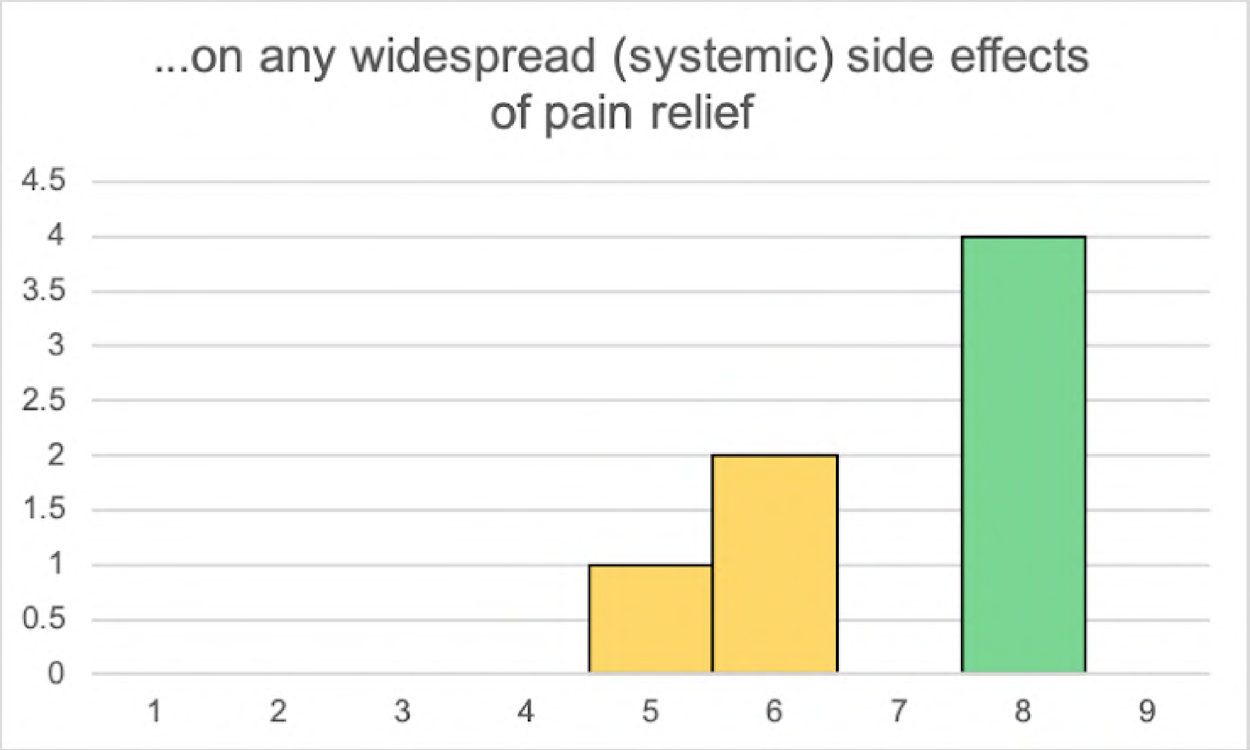

Professionals' responses from round 2: [no consensus]

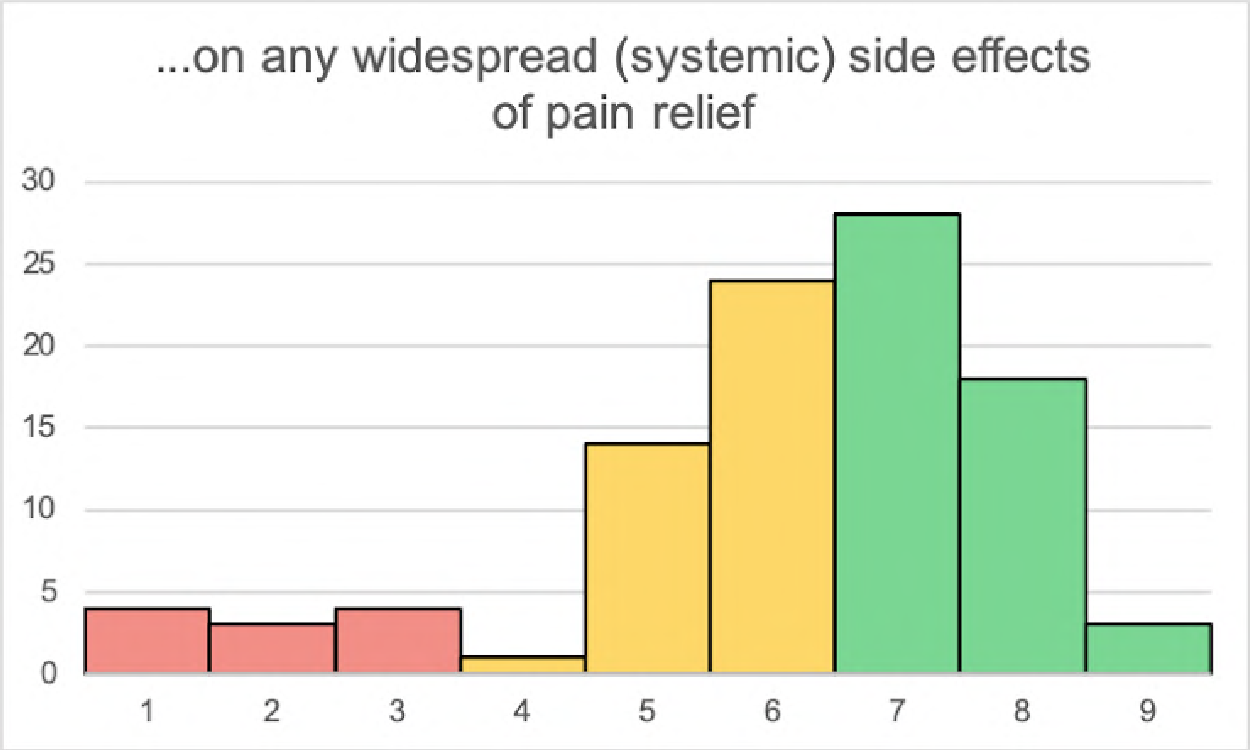

51. Your answer for round 3: Studies should report ...on any widespread (systemic) side effects of pain relief

Mark only one oval.

	1	2	3	4	5	6	7	8	9	
Lowest importance	⬭	⬭	⬭	⬭	⬭	⬭	⬭	⬭	⬭	Highest importance

52. Unable to score/comments

***** THANK YOU *****

That's it!

Thank you for taking part. Your responses will help create the diagnostic criteria and a core outcome set for acute otitis externa. This is round 3 of the process, so you've done everything we've asked of you!

Once you're happy with your responses, please hit submit to record them.

Thank you once again for taking part. If you have any problems at any stage, please email info@entintegrate.org

INTEGRATE DECODE steering committee

This content is neither created nor endorsed by Google.

Google Forms
