# Supplementary material for: Acute otitis externa: Consensus definition, diagnostic criteria and core outcome set development
Source: PLoS One. 2021 May 14;16(5):e0251395. doi: 10.1371/journal.pone.0251395 (PMC8121300; doi:10.1371/journal.pone.0251395)
Supplement: S2 Table — White = no consensus, grey = consensus (+ indicates to include,—to exclude), black = omitted from round having met consensus. (PDF) [file pone.0251395.s004.pdf]

| Candidate outcomes | Round 1 | | Round 2 | | Round 3 | | Committee |
|---|---|---|---|---|---|---|---|
| | Patient | Professional | Patient | Professional | Patient | Professional | |
| The number/frequency of visits to ENT or the GP | + | + | | | | | + |
| The need for pain relief | + | + | | | | | + |
| The need for antibiotics | + | + | | | | | + |
| Improvements in itchiness in the ear | + | + | | | | | + |
| Improvements in hearing impairment | + | + | | | | | + |
| Improvements in ear pain (otalgia) | + | + | | | | | + |
| Improvements in ear canal swelling (oedema) | + | + | | | | | + |
| Improvements in ear canal redness (erythema) | + | + | | | | | + |
| Improvements in discharge from the ear (otorrhoea) | + | + | | | | | + |
| Spread of infection beyond the ear canal | + | + | | | | | + |
| If patients were compliant with the treatment | + | + | | | | | + |
| If patients completed the course of treatment | + | + | | | | | + |
| The number of times the treatment is administered | + | + | | | | | + |
| The need for overnight or in-hospital care | ? | + | + | | | | + |
| The ease of applying the treatment | ? | ? | + | + | | | + |
| Results of bacterial or fungal growth on an ear swab | ? | ? | + | + | | | + |
| Patient satisfaction with their access to appropriate care (for diagnosis & treatment) | + | ? | | + | | | + |
| Patient satisfaction with the required frequency of the treatments | + | ? | | + | | | + |
| Patient satisfaction with the length of their symptoms | + | ? | | + | | | + |
| Patient satisfaction with length of treatment, including number of visits to specialist services (ENT) | + | ? | | + | | | + |
| Improvements in any pain on pressing just in front of the ear canal (tragal tenderness) | + | ? | | + | | | + |
| Widespread (systemic) side effects of treatments | ? | + | + | | | | + |
| Time off work | + | ? | | + | | | + |
| Local side effects of treatments | ? | + | + | | | | + |
| Impact on quality of life | + | ? | | + | | | + |
| Time to resolution of symptoms | | | + | + | | | + |
| Improvements in reactive over-healing in the ear canal (granulations) | ? | + | ? | | + | + | + |
| Widespread (systemic) side effects of pain relief | ? | ? | ? | ? | + | + | + |
| Impact on the ability to work | ? | ? | + | ? | + | + | + |
| Impact on sleep | + | ? | | ? | + | + | + |
| Patient satisfaction with the time required for outpatient appointments | + | ? | | ? | ? | ? | - |
| Improvements in the feeling of anxiety | ? | ? | ? | ? | ? | ? | - |
| Improvements in jaw pain | ? | ? | ? | ? | ? | ? | - |
| Improvements in odour relating to the ear | ? | ? | + | ? | ? | + | - |
| Improvements in a feeling of generalised weakness | ? | ? | ? | ? | ? | ? | - |
| Improvements in a feeling of fullness in the ear (aural fullness) | + | ? | | ? | ? | ? | - |
| Improvement in the ability to concentrate | ? | ? | ? | - | ? | - | - |
| Impact on the ability to socialise | ? | ? | + | ? | ? | ? | - |
| Impact on mental health | ? | ? | ? | ? | ? | ? | - |
| Impact on hours of bed rest | ? | ? | ? | ? | ? | ? | - |
| Impact on activities of daily living or performing household tasks | + | ? | | ? | ? | ? | - |
| Impact on ability to care for the family | ? | ? | ? | ? | ? | ? | - |
| Death during treatment | ? | ? | ? | ? | + | ? | - |
