# Supplementary material for: Acute otitis externa: Consensus definition, diagnostic criteria and core outcome set development
Source: PLoS One. 2021 May 14;16(5):e0251395. doi: 10.1371/journal.pone.0251395 (PMC8121300; doi:10.1371/journal.pone.0251395)
Supplement: S3 Table — *based on stakeholder feedback this criterion was adapted to ‘wet debris’. White = no consensus, grey = consensus (+ indicates to include,—to exclude), black = omitted from round having met consensus. (PDF) [file pone.0251395.s005.pdf]

Candidate diagnostic criteria	Round 1	Round 2	Round 3	Committee
Aural fullness	?	?	?	-
External auditory canal erythema	?	+		+
External auditory canal granulations	?	?	?	-
External auditory canal oedema	+			+
Generalised lethargy	?	-		-
Hearing impairment Text	?	?	?	-
Itchiness	?	+		+
Jaw pain	?	?	?	-
Microbiological identification of an organism	?	?	?	-
Odour related to the ear	?	?	?	-
Otalgia (ear pain)	+			+
Otorrhoea (discharge from the ear)	+			+
Squamous/wet debris*		?	?	+
Tragal tenderness	?	+		+
